# Molecular Signatures of *JMJD10/*MINA53 in Gastric Cancer

**DOI:** 10.3390/cancers12051141

**Published:** 2020-05-02

**Authors:** Nur Aziz, Yo Han Hong, Min Kyeong Jo, Jin Kyeong Kim, Kyung-Hee Kim, Hassan Ashktorab, Duane T. Smoot, Hoon Hur, Byong Chul Yoo, Jae Youl Cho

**Affiliations:** 1 Department of Integrative Biotechnology, Sungkyunkwan University, and Biomedical Institute for Convergence at SKKU (BICS), Suwon 16419, Korea; nuraziz@skku.edu (N.A.); ghddygks13@naver.com (Y.H.H.); whalsrud1017@naver.com (M.K.J.); rosekim95@naver.com (J.K.K.); 2 Proteomic Analysis Team, Research Institute, National Cancer Center, Goyang 10408, Korea; kyunghee@ncc.re.kr; 3 Department of Medicine, Howard University, Washington, DC 20060, USA; hashktorab@howard.edu; 4 Department of Medicine, Meharry Medical Center, Nashville, TN 37208, USA; dsmoot@mmc.edu; 5 Department of Surgery, Ajou University School of Medicine, and Department of Biomedical Sciences, Ajou University Graduate School of Medicine, 164 Worldcup-ro, Yeongtong-gu, Suwon-si, Gyeonggi-do 16499, Korea; hhcmc75@ajou.ac.kr; 6 Division of Translational Science, Research Institute, National Cancer Center, Goyang 10408, Korea

**Keywords:** *JMJD10*/MINA53, gastric cancer, histone demethylase, KDM

## Abstract

The *JMJD10* gene and its encoded protein MYC-induced nuclear antigen (MINA53) are associated with multiple cancers. Besides having both an oncogenic and tumor suppressor function, the intricate role of *JMJD10* in cancer is complex as it depends on the cancer type. In particular, the functional role of *JMJD10*/MINA53 in gastric cancer has been poorly understood. In this study, we have unraveled the molecular signatures and functional roles of *JMJD10*/MINA53 in gastric cancer by multiple approaches, i.e., multi-omics bioinformatics study, analysis of human gastric cancer tissues, and studies in vitro using knockdown or overexpression strategies in gastric cancer cell lines. The results indicated that the *JMJD10* gene and MINA53 protein are commonly overexpressed in cancer patients. JMJD10/MINA53 is involved in the regulation of proliferation and survival of gastric cancer by controlling cell cycle gene expression. These processes are highly associated with MINA53 enzymatic activity in the regulation of H3K9me3 methylation status and controlling activation of AP-1 signaling pathways. This highlights the oncogenic role of *JMJD10*/MINA53 in gastric cancer and opens the opportunity to develop therapeutic targeting of *JMJD10*/MINA53 in gastric cancer.

## 1. Introduction

The mineral dust-induced gene (*MDIG*, also known as *RIOX2*, *MINA*, or *JMJD10*) is the protein-coding gene for MYC-induced nuclear antigen (MINA53, also known as JMJD10 or RIOX2). MINA53 is a member of the Jumonji-C (JmjC) domain-containing (JMJD) proteins, which are known mostly for epigenetic roles in the regulation of gene expression via histone post-translational modifications (PTMs) [1,2]. Eighteen out of 30 members of the JMJD family possess demethylase activity at multiple lysine residues of histones such as histone 3 lysine 4 (H3K4), H3K9, H3K27, and H4K20 [2]. The involvement of the JMJD-type demethylases in human physiology and diseases has been well documented, for instance, in the development, metabolic diseases, inflammation, and various types of cancer [3,4].

*JMJD10*/MINA53 belongs to a phylogenetically distinct group, known as the “JmjC-only” 2-oxoglutarate (2OG)-oxygenase subfamily, as it has a JmjC domain, which is capable of catalyzing protein hydroxylation (and demethylation via hydroxylation reaction) in a 2OG-dependent oxygenase manner and has no other immediate functional domains [5]. An investigation of biochemical activity revealed that JMJD10 has hydroxylation activity toward Rpl27a [6]. Increasing evidence also suggests that JMJD10 has lysine-(K) demethylase (KDM) activity in various experimental conditions. In particular, studies have reported the involvement of *JMJD10*/MINA53 in histone methylation by antagonizing trimethyl lysine 9 on histone H3 (H3K9me3) [7,8,9,10,11,12,13,14,15]. Later studies indicated that *JMJD10*/MINA53 was also involved in the repression of H3K36me3 [16], H3K27me3, and H4K20me3 [17]. 

The physiological and pathological involvement of *JMJD10*/MINA53 has also been documented, particularly in relation to the immune system and cancer. Several reports suggest the overexpression of *JMJD10*/MINA53 in multiple cancers such as colorectal, lung, esophageal, glioblastoma, lymphoma, cholangiocarcinoma, gingival squamous cell carcinoma, neuroblastoma, liver, pancreatic, multiple myeloma, breast, and gastric cancer [5,10,18,19,20,21,22,23,24,25,26]. An intricate role has also been described for *JMJD10*/MINA53, as overexpression may not always correlate with poor patient prognosis [5]. Supposedly, *JMJD10*/MINA53 might have opposing roles at different stages of tumorigenesis as it exhibits a growth-promoting role in early cancer but a tumor suppressor role in the later stages of the disease [5]. Hence, the molecular signatures and mechanisms of *JMJD10*/MINA53 in cancer largely remain to be elucidated, in particular, the molecular signatures in gastric cancer. To our knowledge, there is only one study of *JMJD10*/MINA53 in gastric cancer [12], which was limited to the determination of expression and its correlation with clinicopathological features.

In 2018, gastric cancer was found to be the fifth most frequently diagnosed cancer and the third leading cause of cancer death worldwide [27]. The mechanisms underlying gastric cancer pathogenesis and progression remain to be investigated extensively. However, the development of targeted therapy has emerged and several treatments have reached the first stages of clinical trials in cancer therapy, including inhibitors of histone methylation modifying enzymes such as disruptor of telomeric silencing 1-like (DOT1L) methyltransferases, enhancer of zeste homolog 2 (EZH2) methyltransferases, and lysine-specific demethylase 1 (LSD1) [28]. The molecular signature and functional role of particular enzymes in specific diseases must be fully understood before developing a therapeutic agent. The molecular signatures of *JMJD10*/MINA53 are largely unknown and remain to be elucidated. To obtain a greater understanding of the role of *JMJD10*/MINA53 in gastric cancer, we have analyzed the *JMJD10* molecular signature in gastric cancer using bioinformatics tools, compared the expression of JMJD10 protein expression in normal and tumor-paired gastric tumor samples, and used silencing and overexpression strategies in vitro in multiple gastric cancer cell lines. Here we have demonstrated the oncogenic property of *JMJD10*/MINA53 in gastric cancer by their ability to control proliferation and survival in cell cycle gene expression. The gene expression is tuned by MINA53 enzymatic activity via the regulation of H3K9me3 methylation status and governs AP-1 signaling activation.

## 2. Results

### 2.1. Oncomine and TCGA Data Indicate that JMJD10 is Overexpressed in Multiple Cancers Including Gastric Cancer

**Figure 1:**
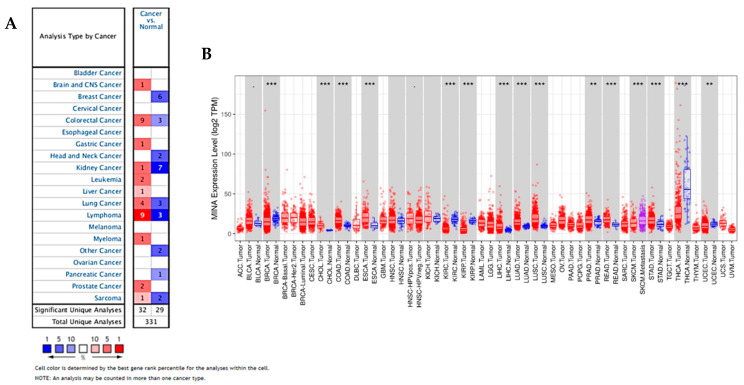
*JMJD10* gene expression is upregulated in diverse types of cancer. (**A**) Expression of the *JMJD10* gene in common cancers vs. normal tissues using the Oncomine database. The graph, generated by Oncomine, represents the number of statistically significant datasets (*p* < 0.01) overexpressed (red) or underexpressed (blue) in JMJD10 mRNA (cancer vs. corresponding normal tissue). The threshold was designed with the following parameters: *p*-value < 0.01, fold change = 1.5, and gene rank = Top 10%; the numbers in the boxes indicate the number of analyses that met the thresholds. (**B**) Expression of JMJD10 in cancer vs. normal tissues in various TCGA tumors using the DiffExp module in TIMER.

After mining the Oncomine database, we obtained 32 significant results indicating the overexpression of *JMJD10* in cancer compared to normal tissues (Figure 1A). Significance was found in several cancer types such as the brain and central nervous system (CNS), colorectal, gastric, kidney, leukemia, liver, lung, lymphoma, myeloma, prostate, and sarcoma. Using a designated threshold, we also obtained 29 unique *JMJD10* results that were underexpressed in several cancer types, i.e., breast, colorectal, head and neck, kidney, lung, lymphoma, pancreatic, sarcoma, and other cancers including adrenal cortex carcinoma and skin basal cell carcinoma. In several subtypes of cancer, i.e., colorectal, kidney, lung, lymphoma, and sarcoma, *JMJD10* expression was both overexpressed and underexpressed because it had exceeded the defined threshold. After considering the number of unique results within each cancer type, we found that *JMJD10* was overexpressed in colorectal cancer and lymphoma while it was underexpressed in kidney cancer and sarcoma. The details of cancer subtypes with *JMJD10* overexpression are provided in Table S1. With regard to gastric cancer type in the Chen Gastric dataset (Table S1), it is evident that *JMJD10*is overexpressed significantly in intestinal-type gastric cancer with a 1.742-fold change compared to normal tissues.

We also explored the expression of *JMJD10* in various tumors in TCGA by using TIMER. As shown in Figure 1B and based on p-value in Table S2, results illustrated that JMJD10 expression was significantly higher in bladder urothelial carcinoma, cholangiocarcinoma, colon adenocarcinoma, esophageal carcinoma, head and neck squamous cell carcinoma, kidney chromophobe, liver hepatocellular carcinoma, lung adenocarcinoma, lung squamous cell carcinoma, prostate adenocarcinoma, rectum adenocarcinoma, and STAD. In addition, *JMJD10* mRNA expression was significantly lower compared to normal patients in breast invasive carcinoma, kidney renal clear cell carcinoma and kidney renal papillary cell carcinoma, skin cutaneous melanoma, thyroid carcinoma, and uterine corpus endometrial carcinoma. Data mining from both the Oncomine and TCGA database suggest that *JMJD10* gene expression was predominantly higher in cancer, including gastric cancer, compared to normal tissues.

### 2.2. JMJD10 Expression in a Microarray of Gastric Cancer Patients from the ACRG Cohort

**Figure 2:**
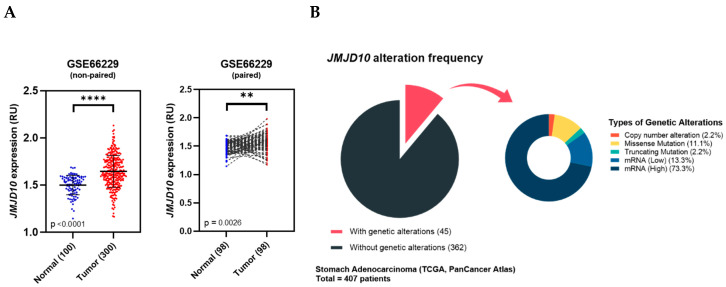
*JMJD10* gene expression in gastric tumor microarray GSE66229 and genetic alteration profiling of *JMJD10* in stomach adenocarcinoma TCGA PanCancer Atlas 2018. (**A**) Pairwise and non-pairwise comparison of JMJD10 expression between normal vs. tumor from dataset GSE66229 (RU: Relative Unit). (**B**) Alteration frequency analysis of the *JMJ10* gene in stomach adenocarcinoma patients from TCGA PanCancer Atlas database extracted from the cBioPortal.

To obtain more focused data of *JMJD10* expression in gastric cancer, we analyzed publicly available microarray datasets from the GEO dataset GSE66229. The analysis was performed using the GEO2R web tool by distinguishing the normal and gastric tumor group followed by retrieving the sample values to be analyzed and plotting the data using GraphPad Prism. As shown in Figure 2A, the high expression of *JMJD10*occurred in tumors compared to normal tissues in both pairwise and non-paired sample analysis. 

### 2.3. Genetic Alteration of JMJD10 in the Gastric Cancer TCGA Cohort

We used STAD TCGA PanCancer Atlas cohorts for genetic alteration analysis. As shown in Figure 2B, 45 out of 407 patients (11%) have genetic aberrations in *JMJD10*. The small pie chart indicates the breakdown of each type of genetic alteration in *JMJD10* among the 45 patients. As indicated, the most frequent alteration types were the upregulation of *JMJD10* mRNA, which accounted for 73.3%. In addition, the types and number of mutations were as follows: copy number alteration = 1, missense mutation = 5, truncating mutation = 1, and mRNA low = 6.

### 2.4. Analysis of MINA53 Protein Expression in Human Gastric Cancer Tissues

**Figure 3:**
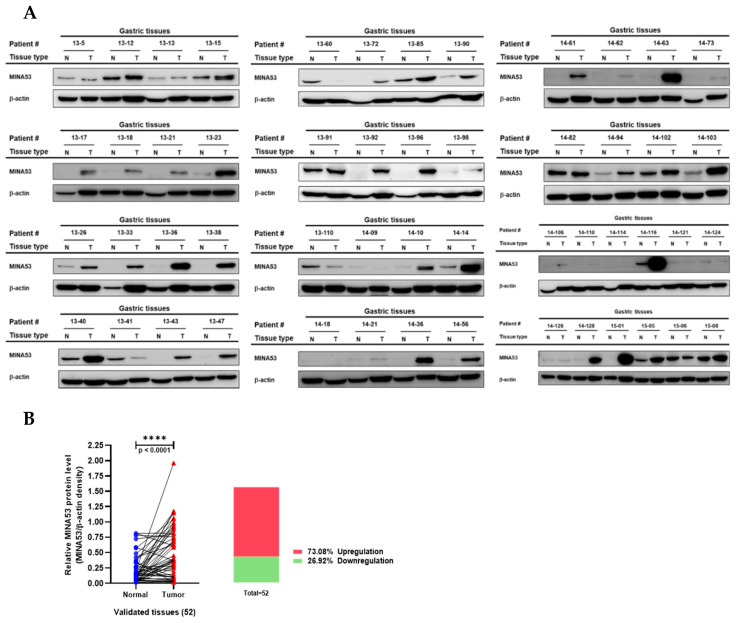
MINA53 protein expression analysis in human gastric cancer tissues. (**A**) Immunoblot results of human gastric cancer and adjacent normal tissues. (**B**) Pairwise analysis and box-plot overview of the relative MINA53 protein expression comparison from validated human gastric cancer tissue samples; normalized expression was done by measuring the relative density of MINA53/β-actin immunoblot results. Statistical significance was calculated using the paired t-test. The whole Western blot images please find in Figure S1.

Immunoblot analysis of samples from pairs of tumor and normal human gastric cancer tissue is presented in Figure 3A. A pairwise analysis and box-plot graph are used to visualize normalized calculated MINA53 expression differences between normal and tumor tissue (Figure 3B). Using median cut-off as shown in the bar-plot of Figure 3B, the MINA53 protein was overexpressed in the tumor tissue of about 73.08% of patients. These results indicate that levels of both *JMJD10* gene expression and its encoded protein, MINA53, is relatively high in gastric cancer compared to normal tissues. The characteristics of patients with gastric cancer used in this study are provided in Table 1.

### 2.5. MINA53 Silencing Inhibited Proliferation Rate and Survival Ability of Gastric Cancer Cell Lines

**Figure 4:**
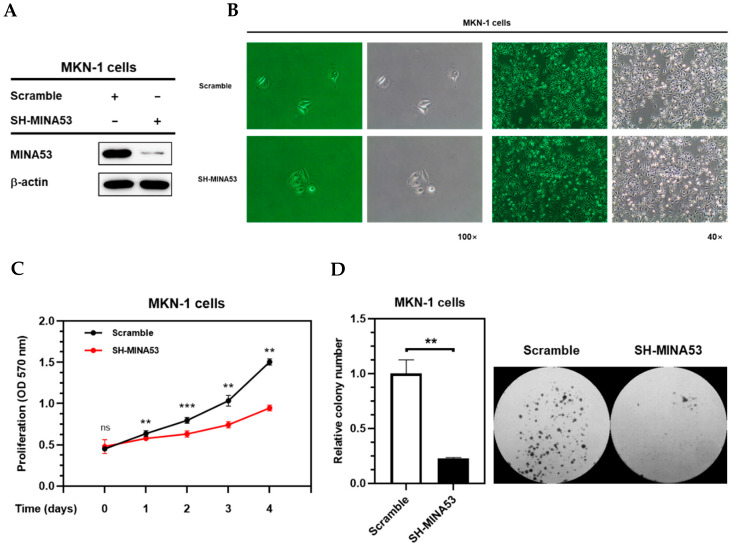
The silencing of MINA53 inhibited proliferation and survival in gastric cancer cell lines. (**A**) Knockdown level of MINA-53 silenced MKN-1 cells. (**B**) Morphology of MINA53-silenced MKN-1 cells compared to scramble MKN-1 cells. (**C**) Proliferation assay of MINA53-silenced MKN-1 cells using MTT assay. (**D**) Colony formation of MINA53-silenced MKN-1 cells after 8 days. The whole Western blot images please find in Figure S1.

The silencing of MINA53 was done in MKN-1 and MKN-45 cells. The knockdown level of MINA53-silenced MKN-1 cells was confirmed by immunoblotting (Figure 4A). The cell morphology was captured under 40 and 100 × magnification using a microscope (Olympus, Tokyo, Japan) (Figure 4B). The proliferation rate was compared using an MTT assay, as shown in Figure 4C. The silencing of MINA53 inhibited the proliferation of MKN-1 cells. The in vitro survival assay was performed using colony formation assay. As shown in Figure 4D, knockdown of MINA53 also decreased colony formation in MKN-1 cells. These results indicated the involvement of MINA53 in the proliferation and survival of gastric cancer cells.

### 2.6. MINA53 is Involved in the Regulation of Cell Cycle-Related Gene Expression in Gastric Cancer Cell Lines

**Figure 5:**
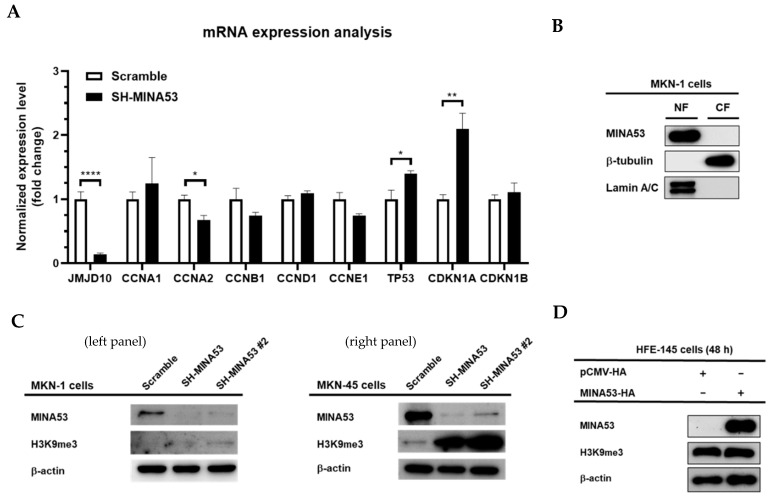
Molecular signature of MINA53 in gastric cancer cell lines. (**A**) mRNA expression analysis of JMJD10 in MINA53-silenced MKN-1 cells. (**B**) MINA53 mainly localized in the nuclear fraction; Lamin A/C and β-tubulin were used as loading controls for nuclear and cytosolic fractions, respectively. (**C**) Analysis of H3K9me3 level in MINA53-silenced MKN-1 cells (left panel) and MKN-45 cells (right panel). (**D**) Overexpression of MINA53 in human gastric normal cell lines (HFE-145) does not affect the H3K9me3 level. The whole Western blot images please find in Figure S1.

Analysis at the molecular level was initially performed by mRNA expression analysis in MINA53-silenced MKN-1 cells using real-time PCR. As shown in Figure 5A, the mRNA expression of *JMJD10* was significantly decreased by up to 88%, and some cell cycle genes were also significantly decreased, such as Cyclin A2 (*CCNA2*), p53 (*TP53*), and p21 (*CDKN1A*). These results emphasized the involvement of *JMJD10* in the regulation of cell cycle gene expression.

### 2.7. MINA53 Regulation of Histone 3 Lysine 9 Methylation in Gastric Cancer Cell Lines

To obtain a greater understanding of the regulatory role of MINA53 in gastric cancer, we performed nuclear fractionation to search for the major localization of this protein in gastric cancer cell line MKN-1. As shown in Figure 5B, the main localization of MINA53 is in the nucleus. Since MINA53 mainly localized in the nuclear fraction, we posited that MINA53 might also have histone modification activity similar to that reported previously in different types of cancer cells. To confirm this, we made 2 different knockdown cell lines, MKN-1 and MKN-45 cells. The knockdown level was confirmed using immunoblot as shown in Figure 5C. Furthermore, we analyzed the histone 3 lysine 9 tri-methyl (H3K9me3) level using these lysates and the results indicated an increased level of H3K9me3 in knockdown cell lines in both MKN-1 and MKN-45 cells. In addition, we overexpressed MINA53 in the normal gastric cell line, HFE-145, and found no difference compared to the control (Figure 5D). Taken together, these results suggest the regulatory function of MINA53 in the H3K9me3 level is possibly due to demethylation activity. The regulation on cell cycle gene expression might be a direct result of the epigenetic role of MINA53 on H3K9 methylation status.

### 2.8. Functional Enrichment Analysis of JMJD10 in Stomach Adenocarcinoma 

**Figure 6:**
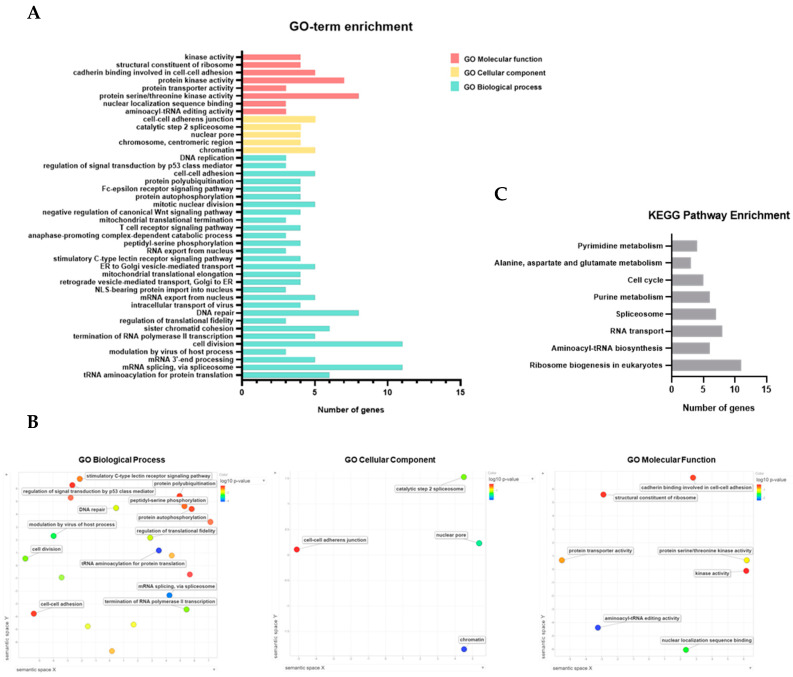
Functional enrichment analysis of *JMJD10* in stomach adenocarcinoma. (**A**) Box plot representing enriched GO terms includes GO molecular function, cellular component, and biological process enrichment. (**B**) Semantic similarity-based scatterplots of GO-term enrichment results; the axes have no intrinsic meaning; semantically similar GO terms are closer together in the plot. (**C**) Box plot representing KEGG pathway enrichment results.

To obtain a better understanding of the functional role of *JMJD10* in gastric cancer, we also performed functional enrichment analysis of gene sets co-expressed with *JMJD10* in STAD tumor TCGA. The list of co-expressed genes is provided in File S3: GO term and KEGG enrichment. Functional annotation clustering within GO biological processes, cellular components, and molecular function, and KEGG pathway are also provided in File S3: GO term and KEGG enrichment. GO-term gene enrichment results are presented in Figure 6A. Within the enrichment threshold *p* < 0.05, the following biological processes were enriched for the genes co-expressed with *JMJD10* in STAD tumor: GO:0006418 tRNA aminoacylation for protein translation, GO:0000398 mRNA splicing via the spliceosome, GO:0031124 mRNA 3′-end processing, GO:0019054 modulation by virus of host process, GO:0051301 cell division, GO:0006369 termination of RNA polymerase II transcription, GO:0007062 sister chromatid cohesion, GO:0006450 regulation of translational fidelity, GO:0006281 DNA repair, GO:0075733 intracellular transport of virus, GO:0006406 mRNA export from the nucleus, and GO:0006607 nuclear localization signal (NLS)-bearing protein import into the nucleus. The following were GO cellular components enriched within the threshold: GO:0000785 chromatin, GO:0000775 chromosome, centromeric region, and GO:0005643 nuclear pore. The following were GO molecular functions enriched within the threshold: GO:0008139 aminoacyl-tRNA editing activity and GO:0008139nuclear localization sequence binding. As shown in Figure 6A, GO terms enriched with more than 5 genes include GO:0000398 mRNA splicing via the spliceosome, GO:0051301 cell division, GO:0006281 DNA repair, GO:0004674 protein serine/threonine kinase activity, GO:0004672 protein kinase activity, GO:0006418tRNA aminoacylation for protein translation, and GO:0007062sister chromatid cohesion. 

Semantic similarity-based scatterplots of GO terms enrichment results were made in REVIGO (Figure 6B). The figure represents the semantic similarity after removing redundant GO-term enrichment results. The closeness between nodes represents the similarity between enriched GO terms. As depicted in Figure 6B, the *JMJD10* co-expressed gene set was enriched in GO biological processes, such as regulation of signal transduction by p53 class mediator and stimulatory C-type lectin receptor signaling pathway, which are closely coordinated in the plot. Peptidyl-serine phosphorylation, protein autophosphorylation, and protein polyubiquitination were also enriched. KEGG pathway enrichment results are shown in Figure 6C. The following were KEGG pathway terms enriched within the threshold: hsa03008 ribosome biogenesis in eukaryotes, hsa00970 aminoacyl-tRNA biosynthesis hsa03013 RNA transport, hsa03040 spliceosome, hsa00230 purine metabolism, and hsa04110 cell cycle. KEGG pathway enriched results indicated that hsa03008 ribosome biogenesis in eukaryotes is the pathway most related to *JMJD10* co-expressed gene sets (Figure 6C).

The functional clustering of the *JMJD10* co-expressed gene set indicates the related biological theme. As presented in supplementary Files, the enriched GO-term and KEGG combined clustering produced 12 clusters with a general biological theme from the highest enrichment score as follows: cluster 1, tRNA regulation of protein translation; cluster 2, spliceosome; cluster 3, cell division regulation; cluster 4, mRNA processing; cluster 5, intracellular transport of virus and protein transporter activity; cluster 6, DNA repair and regulation of signal transduction by p53 class mediator; cluster 7, ER and Golgi vesicle-mediated transport; cluster 8, T-cell/C-type lectin/Fc-epsilon receptor signaling pathway; cluster 9, protein kinase activity, cell cycle, and negative regulation of apoptotic processes; cluster 10, structural constituent of ribosomes and mitochondrial translational elongation/termination; cluster 11, anaphase-promoting complex-dependent catabolic process; and cluster 12, cell-cell adhesion. Taken together, enrichment results highlight the function of *JMJD10* co-expressed genes in regulating the cell division and cell cycle, which might justify the lower proliferation rates and decreased colony formation of MINA53-silenced MKN-1 cells found in vitro.

### 2.9. The Regulatory Function of MINA53 in the AP-1 Signaling Pathway in Gastric Cancer Cell Lines 

**Figure 7:**
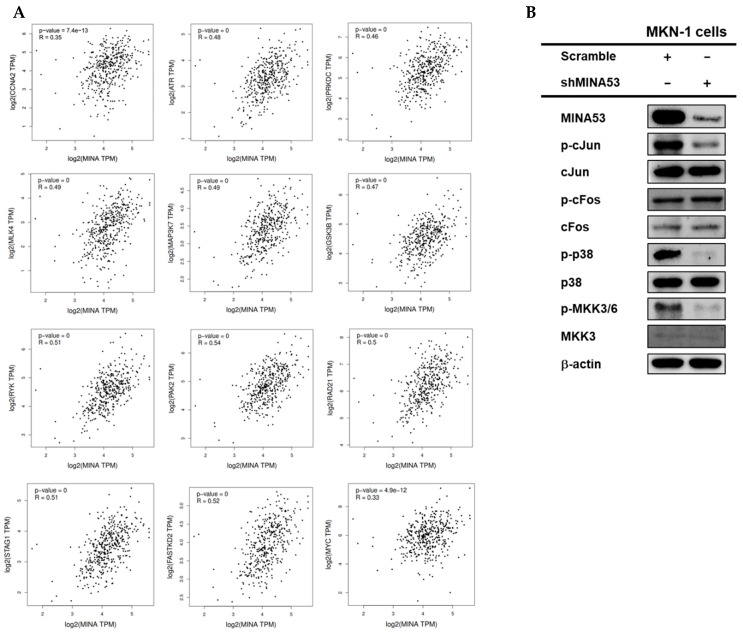
Regulatory role of MINA53 in AP-1 signaling in gastric cancer cell lines. (**A**) Gene expression correlation with *JMJD10* in the STAD tumor dataset using GEPIA. (**B**) Immunoblot analysis of AP-1 signaling activation in MINA53-silenced MKN-1 cells. The whole Western blot images please find in Figure S1.

Functional clustering indicates the presence of cell cycle, kinase activity, and MAPK cascade enriched genes in clusters 9 and 11. We sought to determine the regulatory function of MINA53 in the AP-1 signaling pathway since this pathway has also been associated with the regulation of the cell cycle and cell survival. Figure 7A represents enriched genes within clusters of cell cycle and kinase activity as follows: ATR serine/threonine kinase (ATR), glycogen synthase kinase 3 beta (GSK3B), p21 (RAC1) activated kinase 2 (PAK2), protein kinase, DNA-activated, catalytic polypeptide (PRKDC), receptor-like tyrosine kinase (RYK), FAST kinase domains 2 (FASTKD2), mitogen-activated protein kinase kinase kinase 7 (MAP3K7), RAD21 cohesin complex component (RAD21), stromal antigen 1 (STAG1), and mixed lineage kinase 4 (MLK4). All of these genes have a positive correlation with JMJD10 expression. In addition, the positive correlation of CCNA2 with JMJD10 was also considered significant in PCC = 0.35, which is in agreement with the real-time PCR results in MKN-1 cells. JMJD10 has been reported to be tightly associated with the MYC oncogene, the results also indicated a significant positive correlation with PCC = 0.33.

Immunoblot analysis of the AP-1 signaling pathway in Figure 7B shows the inhibition of AP-1 signaling activation as indicated by the decrease of phospho-kinase activation in the MINA53 knockdown cell lines. This includes a decrease in the phosphorylation of cJun, p-38, and MKK3/6. These results also support the positive correlation of JMJD10 with 2 members of the MAPK/AP1 pathway, MAP3K7 and MLK4, which was determined from the functional clustering results.

## 3. Discussion

The expression of *JMJD10/*MINA53 in various types of cancer has been well documented. However, the molecular mechanism of *JMJD10*/MINA53 regulation in cancer is poorly understood. A recent review discussed the intricate mechanism of *JMJD10*/MINA53 in tumorigenesis, where it can act as both a tumor suppressor and tumor promoter gene [5]. A more complex mechanism has also been associated with types of cancers. As discussed by Bundred et al [5], *JMJD10* can act as a tumor promoter gene by promoting cancer cell growth and decreasing patient prognosis and might also act as a tumor suppressor by regulating cancer invasion and metastasis, which depends on cancer type. In particular, the functional and regulatory role of *JMJD10/*MINA53 in gastric cancer is poorly understood. To obtain a greater understanding of this matter, we studied the functional and regulatory role of *JMJD10/*MINA53 in gastric cancer by exploring their molecular signatures using multiple approaches, i.e., bioinformatics tools, clinical samples analysis, and in vitro experiments using gastric cancer cell lines.

Data mining of publicly available cancer databases (Oncomine and TCGA) suggests that the *JMJD10* gene is overexpressed in a majority of cancer types, including gastric cancer as shown in Figure 1. However, our analysis also highlighted that in several types of cancer, *JMJD10* gene expression is significantly decreased. Furthermore, the pairwise and non-paired analysis of gastric tumors in microarray data from the ACRG dataset also indicated significant increases in tumors compared to normal tissues (Figure 2A). Genetic aberration analysis from the STAD (TCGA PanCancer) Atlas showed a variety of genetic alterations in *JMJD10* found in 45 out of 407 patients. These alterations include copy number alteration (denoted as amplification), missense and truncating mutations with unknown significance, and high and low mRNA. The relevance and significance of these mutations in gastric cancer have not been studied. In vitro mutation studies might be useful in identifying the function and significance of this mutation in gastric cancer. 

*JMJD10* encoded protein, MINA53, expression in gastric cancer was also determined in this study. Among 52 patients, 73.08% showed an upregulation of MINA53 expression while 26% showed a downregulation (Figure 2). These results suggest that both the *JMJD10* gene and protein (MINA53) were upregulated in gastric cancer compared to normal tissues. The knockdown of MINA53 in gastric cancer cell lines resulted in a decreased proliferation rate and colony formation compared to scrambled cell lines (Figure 3). The results implied the involvement of MINA53 in the proliferation and survival of gastric cancer. Molecular elucidation by real-time PCR showed involvement of MINA53 in the regulation of cell cycle-related genes, i.e., Cyclin A (*CCNA2*), p53 (*TP53*), and p21 (*CDKN1A*). These genes have been associated with proliferation and survival in cells by regulating cell cycle progression. Cyclin A has been denoted as a positive regulator of cell cycle progression while p53 and p21 are negative regulators [29]. When MINA53 is knocked down, the expression of cell cyclin A genes was decreased while the negative regulation of cell cycle progression by p53 and p21 expression was increased. 

Since MINA53 mainly localized in the nucleus (Figure 3B), it raises the possibility that the regulation of gene expression might involve the epigenetic activity of MINA53, which has been reported in certain types of cancer but is unknown in gastric cancer. Here we have provided evidence that knockdown of MINA53 in gastric cancer cell lines also affected H3K9 methylation status. MINA53-silenced gastric cell lines showed an increased level of methylation compared to scramble control cells (Figure 3C). This result suggested that the regulation of MINA53 might be due to the demethylase activity of H3K9 methylation. Intriguingly, when we overexpressed MINA53 in human normal gastric cell lines, there was no significant difference in the H3K9 methylation level. This might be interpreted as two possibilities; first, the demethylase activity only occurs in cancer cells, and, second, the demethylase activity is cell line dependent. Further studies must be conducted to obtain a clear understanding of these issues.

To obtain a greater understanding of the regulatory role of JMJD10 in gastric cancer, we performed a co-expression analysis. Co-expression analysis has been used to predict gene function and help to identify the roles of the genes within phenotypic differences [30]. We performed functional enrichment analysis of gene sets co-expressed with JMJD10 in STAD tumor TCGA using functional annotation analysis in DAVID 6.8. GO-term enrichment (Figure 4A,B) and KEGG pathway enrichment (Figure 4C) results indicate the enriched terms in JMJD10 co-expressed gene sets. As described in the Results section, 12 functional clusters have been generated (Supplementary File). The highest functional cluster was related to tRNA regulation for protein translation, which is also correlated with previously known substrates linked to the regulation of ribosomal biogenesis by the hydroxylation of Rpl27a in ribosomes [6,31]. The cluster, on which we are focusing, includes the enriched genes within the cell cycle and kinase activity terms. This result is in agreement with the in vitro results that *JMJD10* is involved in the regulation of cell cycle genes in gastric cancer cell lines as verified using knockdown conditions. Moreover, co-expressed genes were also enriched in cell division terms, which might take part in the regulation of proliferation and survival. 

Each of the enriched genes within the cell cycle terms was then subjected to analysis. The genes include *ATR*, *GSK3B*, *PAK2*, *PRKDC*, *RYK*, *FASTKD2*, *MAP3K7*, *RAD21*, *STAG1*, and *MLK4*. All of these genes are positively correlated with *JMJD10* in gastric cancer patients and predicted to be major co-players with *JMJD10* in cell cycle regulation in gastric cancer. The correlation of each of these genes with *JMJD10* is presented in Figure 7A. In addition, the correlation between *CCNA2* and *MYC* is also included in Figure 7A. The positive correlation of *CCNA2* is linear and obtained from real-time results using MINA53 knockdown cell lines. As shown in Figure 7A, *MYC* gene expression is also positively correlated with *JMJD10* expression. Furthermore, yet interestingly, two of the genes within the enriched cell cycle clusters, namely *MAP3K7* and *MLK4*, are known as players in the MAPK/AP-1 signaling pathway. AP-1 signaling pathways have been widely known to be tightly associated with cancer through the regulation of proliferation and tumorigenesis [32]. As the regulatory function of *JMJD10* on AP-1 signaling has not been elucidated, here we analyzed AP-1 signaling activation in MINA53-silenced MKN-1 cells by immunoblotting. The results showed that the AP-1 signaling activation was suppressed in MINA53 knockdown cells as indicated by the decrease in cJun, p38, MKK3/6, and MLK3 phosphorylation, which are downstream of MAP3K7 and MLK4. These results suggested that MINA53 regulates proliferation and tumorigenesis in part by the regulation of AP-1 signaling activation possibly via controlling gene expression or kinase activity of MAP3K7 and MLK4.

## 4. Materials and Methods

### 4.1. JMJD10 mRNA Expression Analysis in Cancer Using Oncomine and TIMER

*JMJD10* mRNA expression in various cancer types was retrieved from the Oncomine database (https://www.oncomine.org) [33,34]. This database platform is the largest oncogene database containing 715 data sets and 86,733 samples with expertly curated data [33,34]. The mRNA expression of *JMJD10* between cancer tissues and their normal tissue counterparts were calculated using the threshold parameters: *p*-value, 0.01; fold change, 1.5; gene rank, top 10%; data type, mRNA. Details of each cancer subtype analysis within these thresholds are summarized in Table S1. Differential gene expression between tumor and adjacent normal tissues for the *JMJD10* gene across all The Cancer Genome Atlas (TCGA) tumors was analyzed using the DiffExp module in the Tumor IMMune Estimation Resource (TIMER) webserver [35]. The TIMER web server is a comprehensive resource for systematical analysis of immune infiltrates across diverse cancer types equipped with a DiffExp module to explore differential gene expression between tumor and normal tissue [35]. Statistical significance was calculated using the Wilcoxon test. Details of the p-value in each tumor were presented in Table S2. 

### 4.2. JMJD10 Gene Expression in the Asian Cancer Research Group (ACRG) GEO Array Dataset

The GEO2R web tool (http://www.ncbi.nlm.nih.gov/geo/geo2r/; [36]) was used to retrieve the *JMJD10* gene expression value from the GEO dataset. We analyzed GSE66229, a GEO dataset of expression profiling by array, comprising 100 normal gastric tissues and 300 gastric tumor tissues collected by ACRG [37]. Groups were defined manually using GEO2R, sample values were assessed by entering the *JMJD10* probe ID. Pairwise comparison was made by manually distinguishing values of tumors with adjacent normal tissues, followed by statistical analysis and back plotting using GraphPad Prism software. Pairwise and non-paired sample values from the GSE66229 dataset are provided in File S1: expression values of the GSE dataset. 

### 4.3. Frequency of JMJD10 Gene Alteration Analysis

The alteration frequency of *JMJD10* was obtained from the online cBioPortal for Cancer Genomics (http://www.cbioportal.org) [38,39]. The Stomach Adenocarcinoma dataset (TCGA, PanCancer Atlas), comprising 407 total patients, was selected. Genomic profiles include mutation, putative copy number alterations from GISTIC, and mRNA expression z-scores (RNA Seq V2 RSEM) with z-score threshold ± 2 were selected for RIOX2 query genes. Details of gene alteration frequency are presented in File S2: gene alteration frequency. Analyzed results were back plotted using GraphPad Prism software.

### 4.4. Preparation of Gastric Tumor Tissue Lysate and Immunoblotting

A total of 52 pairs of human gastric tumors and adjacent healthy tissues were obtained from patients who underwent surgical resection of gastric tumors at the Ajou University Hospital. Specimens were collected at the Ajou Human Bio-Resource Bank, and then frozen at −80 ℃ until use. Written informed consent for the storage and usage of their specimens was obtained from all patients. The present study was conducted in accordance with the ethics code of the World Medical Association (Declaration of Helsinki) and was approved by the Institutional Review Board of Ajou University Hospital (AJIRB-BMR-KSP-19-059). The clinicopathological assessment of the patients is listed in Table 1. The frozen tissues specimens were ground with liquid nitrogen followed by homogenization in lysis buffer (50 mM Tris-HCl pH 7.5, 20 mM NaF, 25 mM β-glycerolphosphate pH 7.5, 120 mM NaCl, 2% NP-40, 2 μg/mL leupeptin, 2 μg/mL aprotinin, 2 μg/mL pepstatin A, 0.1 mM Na_3_VO_4_, 1 mM benzamide, 0.1 mM PMSF, and 1.6 mM pervanadate). Tissue homogenates were incubated on ice for 10 min with frequent vortexing and centrifuged at 12,000 × g for 10 min at 4 °C. The supernatant was collected and the protein concentration was determined using the Bradford assay [40]. A total of 30 μg of protein for each sample was used in an immunoblotting assay. The immunoblotting assay was performed initially by separating proteins on 10% SDS-polyacrylamide gels and transferring them by electroblotting to polyvinylidene difluoride (PVDF) membranes. Membranes were then blocked for 1 h with 3% BSA (w/v) in 1× TBST at room temperature. The membranes were incubated overnight with primary antibodies at 4 °C, washed 3 times with 1× TBST, and incubated for an additional 2 h with HRP-conjugated secondary antibodies at room temperature. After washing 3 times with 1× TBST, MINA53 protein was visualized using enhanced peroxidase detection (PicoEPD) according to the manufacturer’s manual (Elpis Biotech, Daejeon, Korea). Relative band intensity was calculated using Image J software (NIH, Bethesda, MA, USA).

### 4.5. Cell Culture

MKN-1, MKN-45, and HFE-145 cells were cultured in RPMI 1640 medium (HyClone Laboratories, South Logan, UT, USA) with 10% heat-inactivated fetal bovine serum (Thermo Fisher Scientific, Waltham, MA, USA), 100 U/mL of penicillin, 100 μg/mL of streptomycin, and 2 mM l-glutamine (Thermo Fisher Scientific, Waltham, MA, USA). HEK293T cells were cultured in DMEM (HyClone Laboratories, South Logan, UT, USA) supplemented with 5% heat-inactivated FBS (Thermo Fisher Scientific, Waltham, MA, USA), 100 U/mL of penicillin, 100 μg/mL of streptomycin, and 2 mM l-glutamine (Thermo Fisher Scientific, Waltham, MA, USA). All cells cell lines were grown at 37 °C in the presence of 5% CO_2_ in a humidified incubator.

### 4.6. shRNA-Mediated Silencing of JMJD10 in Gastric Cancer Cell Lines

Construction of hairpin-pLKO.1 vectors for the generation of shRNA-mediated silencing used methods previously described [41]. To generate lentiviral particles, the following shRNA oligonucleotides targeting JMJD10 coding sequences were used: human shMINA53 (5′-GCAACGATTCAGTTTCACCAA-3′) and human shMINA53 #2 (5′-CAAACATGAGACCTCCCTGTT-3′). The sequences were then cloned into a pLKO.1 vector (gift from David Root (Addgene plasmid # 10878; http://n2t.net/addgene:10878; RRID:Addgene_10878)). The lentiviral particles were produced by transfecting HEK293T with the pLKO.1 plasmid containing shRNA targeting sequences together with the lentiviral constructs (psPAX2 and pMD2.G) using Lipofectamine 2000 (Thermo Fisher Scientific, Waltham, MA, USA). Subsequently, media was filtered and collected 24 h post-transfection. Lentiviral infections were done by treating MKN1 and MKN-45 cells with 0.5 mL of media containing a virus followed by overnight incubation (37 °C, 5% CO_2_). The next day, the media containing virus was replaced with fresh medium for an additional 24 h followed by fresh media containing puromycin 4 µg/mL and 6 µg/mL to select a population of MKN-1 and MKN-45 resistant cells, respectively.

### 4.7. Cell Proliferation Assay

Cell proliferation properties were analyzed using an MTT assay as reported previously [42]. Briefly, MKN-1 scramble cells and MKN-1 shMINA53 cells (1.5 × 10^3^ cells/well) were pre-incubated in 96-well plates (SPL Life Sciences, Pocheon, Korea) After 12 h of incubation (denoted as 0 days), 10 μL of MTT solution (10 mg/mL in PBS pH 7.4) was added to the cell culture for 3 h at 37 °C. The reaction was then stopped by adding 100 μL stop solution (15% sodium dodecyl sulfate), followed by overnight incubation (37°C, 5% CO_2_). The absorbance was measured at 570 nm using a Synergy HT Multi-Mode Microplate Reader (BioTek Instruments GmbH, Bad Friedrichshall, Germany). The same steps were repeated after 24, 48, 72, and 96 h time points.

### 4.8. Colony Formation Assay

Survival was analyzed using a colony formation assay. MKN-1 scramble cells and MKN-1 shMINA53 cells (0.5 × 10^3^ cells/well) were pre-incubated in 6-well plates. After 8-days incubation (37 °C, 5% CO_2_), the visible colonies were fixed with 4% paraformaldehyde for 15 min and stained with 0.1% crystal violet (Sigma Aldrich, St. Louis, MO, USA). The number of colonies was captured by using MiniBIS Bio-Imaging systems and counted automatically using Image J software (NIH, Bethesda, MA, USA).

### 4.9. Reverse-Transcriptase Real-Time PCR

Total RNA was isolated from MKN-1 scramble cells and MKN-1 shMINA53 cells using TRIzol reagent according to the manufacturer’s instructions. RNA from each cell was transcribed using a cDNA synthesis kit (Thermo Fisher Scientific, Waltham, MA, USA) according to the manufacturer’s instructions. The analysis of mRNA using real-time PCR was performed as described previously [43]. The primer sequences used in this study are listed in Table 2.

### 4.10. Preparation of Nuclear and Cytosolic Fractions 

MKN-1 cells (5 × 10^6^ cell/mL) were pre-incubated overnight (37 °C, 5% CO_2_). Nuclear fractionation was conducted using a method described previously [44]. Lamin A/C and β-tubulin were used as loading controls for the nuclear fraction and cytosolic fraction, respectively. 

### 4.11. Plasmid DNA Transfection

The plasmid pCMV-HA-MINA53 was constructed by a standard cloning method. The constructs were confirmed by automated DNA sequencing. HFE-145 cells (2 × 10^6^ cells/well) were pre-incubated overnight (37 °C, 5% CO_2_), transfection of the plasmid was performed using Lipofectamine 2000 (Thermo Fisher Scientific) according to manufacturer’s instructions. After 48 h, the cells were then harvested and followed by immunoblotting analysis.

### 4.12. Functional and Pathway Enrichment Analysis of JMJD10 in Gastric Cancer

The identification of genes co-expressed with *JMJD10* in gastric cancer was performed by using GEPIA (http://gepia.cancer-pku.cn/) [45]. The top 200 genes identified with a Pearson correlation coefficient (PCC) > 0.45 in the stomach adenocarcinoma (STAD) tumor dataset were analyzed for gene-annotation enrichment using DAVID tools (david.ncifcrf.gov) [46]. Gene ontology (GO) biological process, cellular component, and molecular function, and KEGG pathways were selected for enrichment annotations. The top 200 genes identified with PCC and functional annotation clustering results are provided in File S3: GO term and KEGG enrichment. Data were visualized using GraphPad Prism and semantic similarity-based scatterplots using REVIGO [31]. GO terms with *p*-values or false discovery rate (FDR) > 0.50 were excluded from the analysis.

## 5. Conclusions

In summary, we demonstrated that the *JMJD10* gene and MINA53 protein expression are overexpressed in gastric cancer. *JMJD10*/MINA53 regulates the proliferation and survival of gastric cancer cell lines. This involves the MINA53 regulation of H3K9me3 methylation status in part by suppression of AP-1 signaling activation. These findings open up an opportunity for the development of *JMJD10*/MINA53-targeted therapy in gastric cancer.

## Figures and Tables

**Table 1:** The characteristics of gastric cancer patients used in this study.

Characteristic	*n* (%)
Age	
Median	60.5
Range	22–86
Sex	
Male	75
Female	25
Stage	
Ib	13.46
II	
III	34.62
IV	51.92
Location	
Upper third	19.23
Mid third	46.15
Lower third	48.08
Lauren Classification	
Intestinal	42.31
Diffuse	44.23
Mixed	9.62
Indeterminate	3.85
Differentiation	
Well-differentiated carcinoma	1.92
Moderately differentiated carcinoma	30.77
Poorly differentiated carcinoma	26.92
Mucinous carcinoma	15.38
Signet ring cell carcinoma	40.38
Others	7.69
Lymphovascular invasion	
Absent	26.92
Present	73.08
Neural/perineural invasion	
Absent	36.54
Present	63.46

**Table 2:** Primer sequences used for PCR.

PCR Type	Genes Name	Sequence (5′-3′)	
**qPCR**	*GAPDH*	Forward	CAATGAATACGGCTACAGCA
		Reverse	AGGGAGATGCTCAGTGTTGG
	*JMJD10*	Forward	TACCGAGGCTGGACAGTGTA
		Reverse	TTCATCCTCTCCTCGGCTCA
	*CCNA1*	Forward	AGAAAGATAACGACGGGAAGAG
		Reverse	CTGGAAGACGAAATCTGGGAG
	*CCNA2*	Forward	TCTGTGTTCTGTGAATAAAGCA
		Reverse	TTCTTGGATGCCAGTCTTAC
	*CCNB1*	Forward	CAACTTGAGGAAGAGCAAGC
		Reverse	TCTCCTGCAACAACCTGAAT
	*CCND1*	Forward	GAAGTTGCAAAGTCCTGGAGC
		Reverse	ATGGTTTCCACTTCGCAGCA
	*CCNE1*	Forward	GATGAAGAAATGGCCAAAATCG
		Reverse	GCACGTTGATGAGTTTGGGTAAA
	*TP53*	Forward	AAGCAGTCACAGCACATGACGGAG
		Reverse	GAGTCTTCCAGTGAGATGATGGT
	*CDKN1A*	Forward	TGCCGAAGTCAGTTCCTTGTG
		Reverse	GTTCTGACATGGCGCCTCCT
	*CDKN1B*	Forward	TCACAAGGCAGTGATGAAGCA
		Reverse	GGTGTTCACAGAGCCGAACT

## Supplementary Materials

The following are available online at https://www.mdpi.com/2072-6694/12/5/1141/s1, Figure S1: the whole Western blot images, Table S1: *JMJD10* expression in various cancers from the Oncomine database, Table S2: Significantly different expression of *JMJD10* in tumor TCGA by TIMER, File S1: expression values GSE dataset, File S2 gene alteration frequency, File S3: GO term and KEGG enrichment.
