# Supplementary material for: Molecular Signatures of JMJD10/MINA53 in Gastric Cancer
Source: Cancers (Basel). 2020 May 2;12(5):1141. doi: 10.3390/cancers12051141 (PMC7281541; doi:10.3390/cancers12051141)
Supplement: Supplementary file 1 [file cancers-12-01141-s001.zip › cancers-773947 supplementary/cancers-773947 supplementary figure and table.docx]

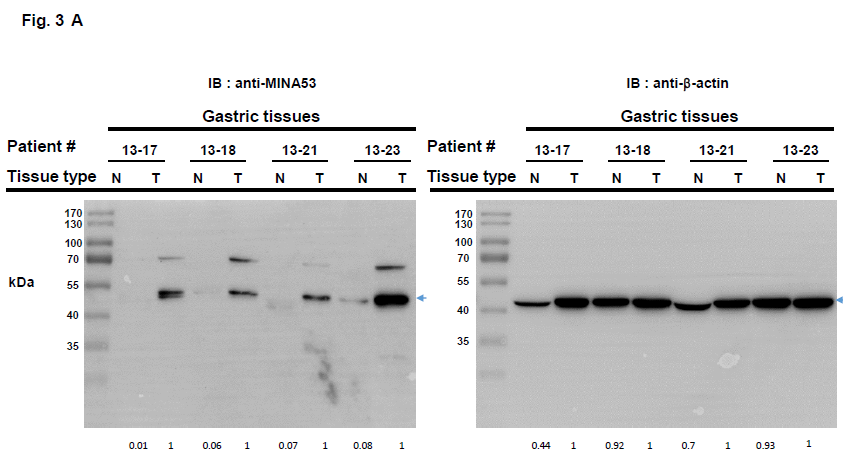


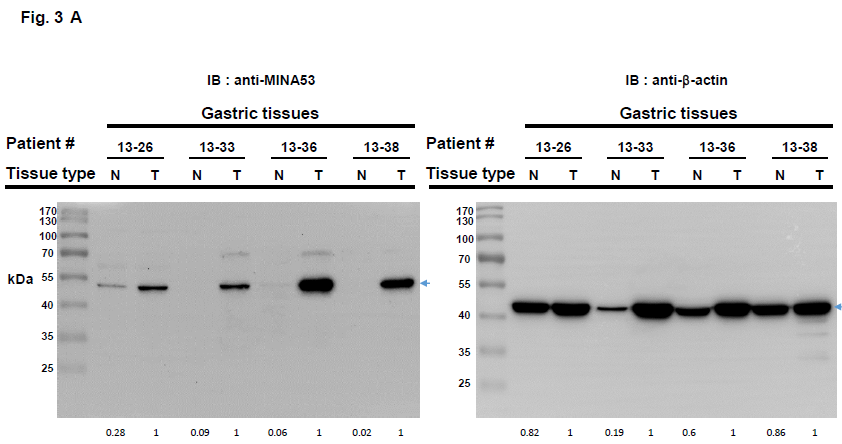


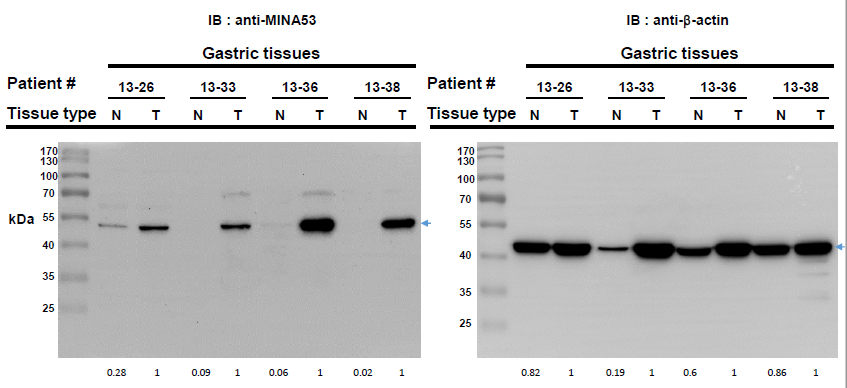


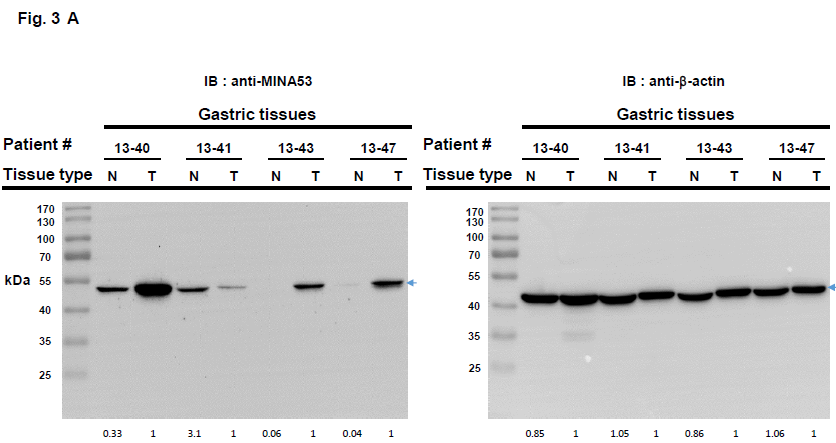


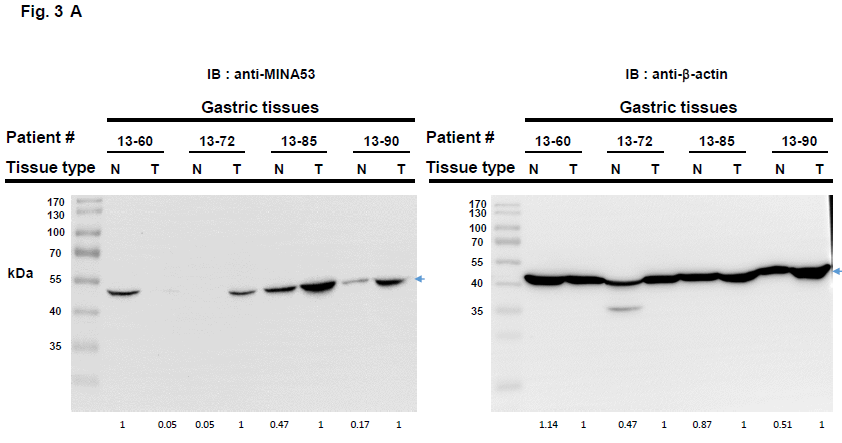


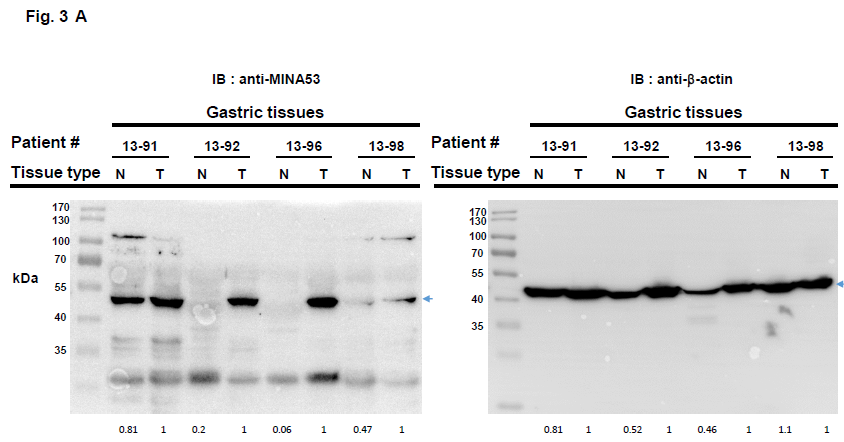


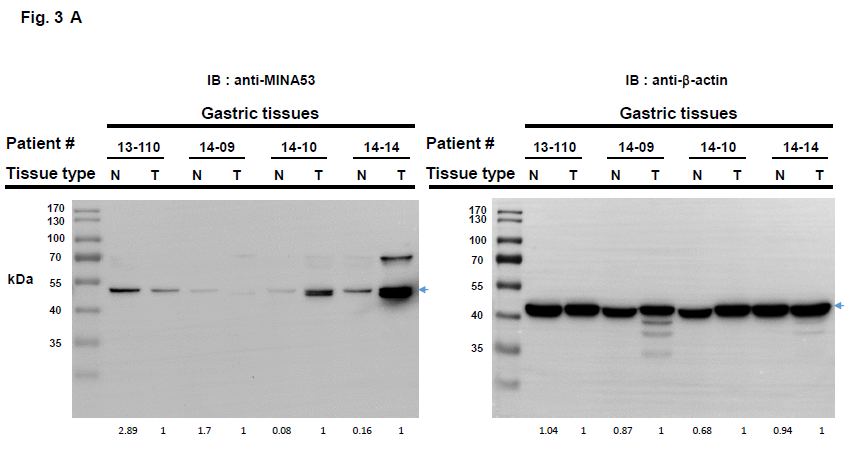


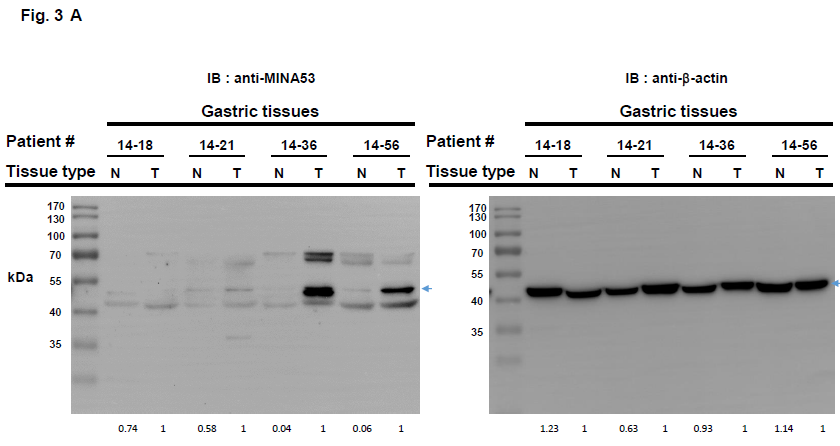


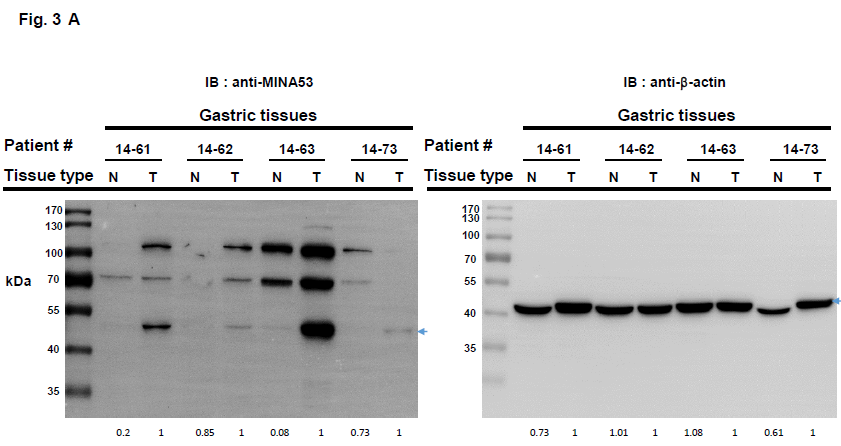


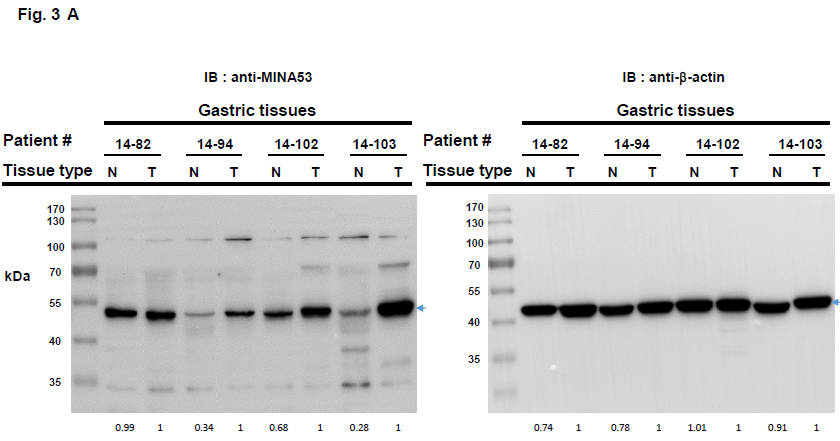


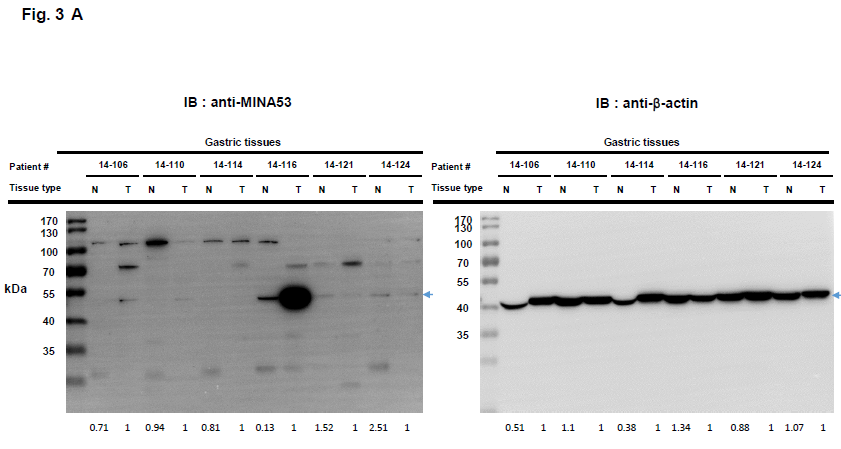


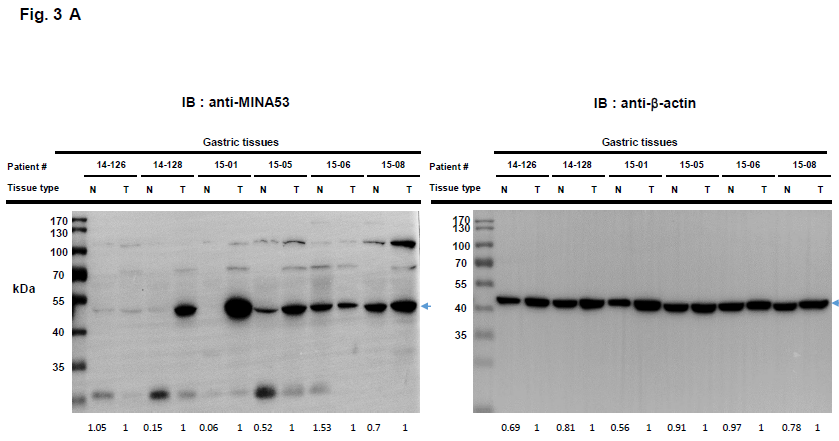


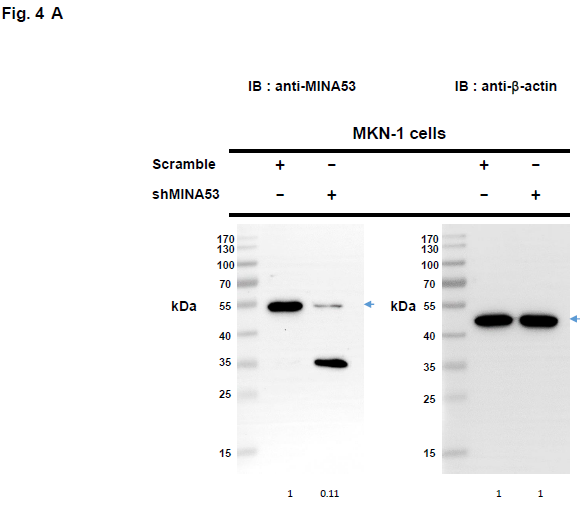


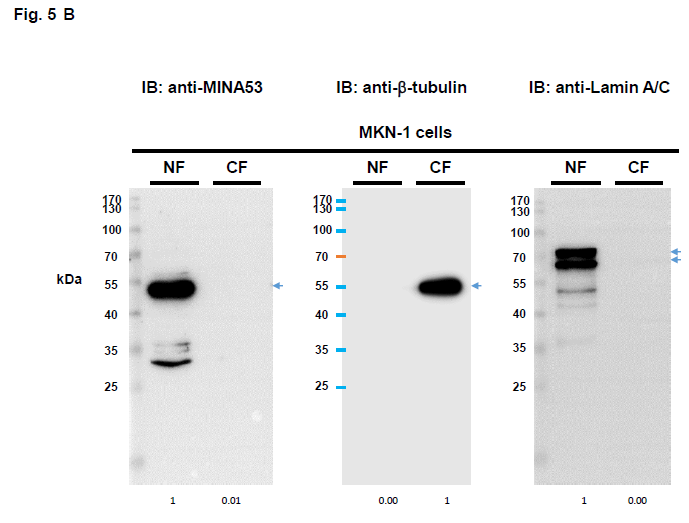


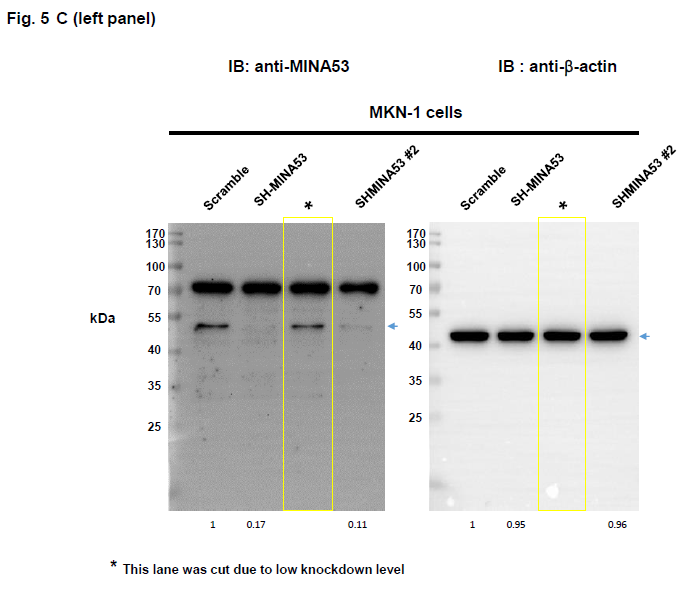


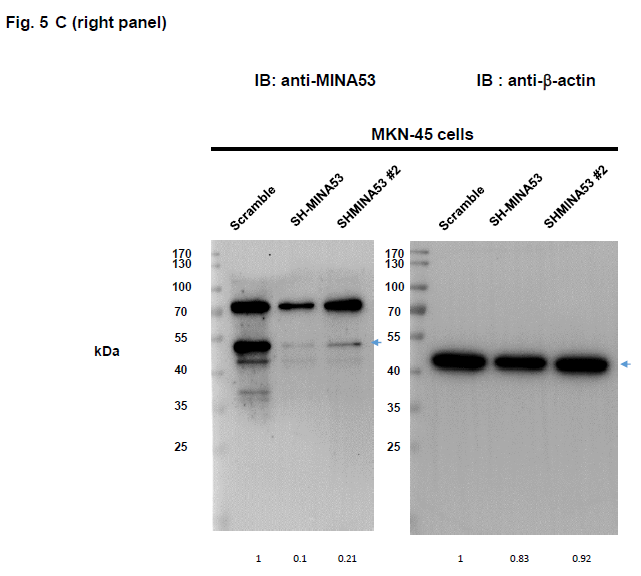


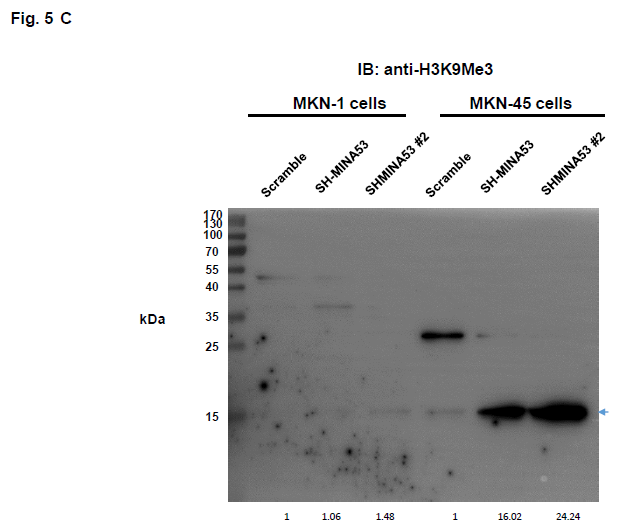


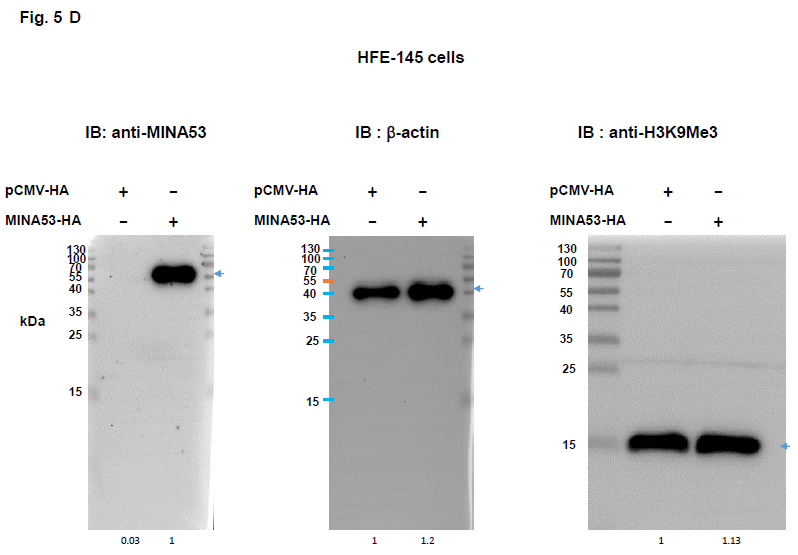


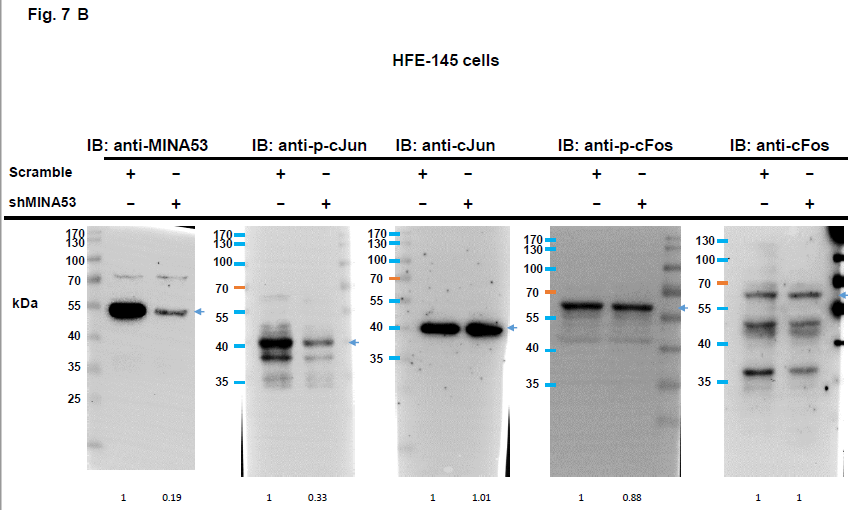


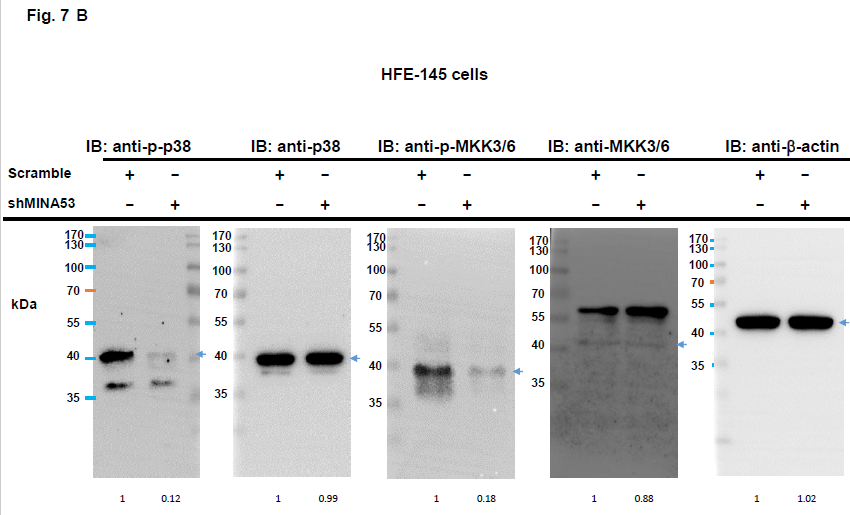


Figure S1. The whole Western blot images.

**Table S1.** *JMJD10* expression in various cancers from the Oncomine database.

| **Cancer** | **Cancer subtype** | **p-value** | **Fold change** | **Rank (%)** | **Sample** | **Reference** |
| --- | --- | --- | --- | --- | --- | --- |
| Brain and CNS | Pilocytic Astrocytoma | 9.74E-4 | 3.863 | 2 | 15 | Gutmann Brain |
| Colorectal | Colon Mucinous Adenocarcinoma | \|  \| 9.20E-12 \| \| --- \| --- \| | 1.770 | 2 | 237 | TCGA Colorectal |
|  | Cecum Adenocarcinoma | 4.02E-11 | 1.835 | 4 | 237 | TCGA Colorectal |
|  | Rectosigmoid Adenocarcinoma | \|  \| 3.76E-4 \| \| --- \| --- \| | 1.807 | 6 | 237 | TCGA Colorectal |
|  | Rectal Adenocarcinoma | 6.73E-14 | 1.868 | 7 | 237 | TCGA Colorectal |
|  | Colon Adenocarcinoma | \|  \| 1.22E-12 \| \| --- \| --- \| | 1.875 | 9 | 237 | TCGA Colorectal |
|  | Rectal Adenoma | \|  \| 1.85E-5 \| \| --- \| --- \| | 2.014 | 7 | 64 | Sabates-Bellver Colon |
|  | Colon Adenoma | \|  \| 5.23E-9 \| \| --- \| --- \| | 1.694 | 10 | 64 | Sabates-Bellver Colon |
|  | Cecum Adenocarcinoma | 3.67E-5 | 1.510 | 9 | 105 | Kaiser Colon |
|  | Colon Mucinous Adenocarcinoma | \|  \| 4.85E-5 \| \| --- \| --- \| | 1.518 | 9 | 105 | Kaiser Colon |
| Gastric | Gastric Intestinal Type Adenocarcinoma | 6.35E-11 | 1.742 | 4 | 132 | Chen Gastric |
| Kidney | Renal Oncocytoma | \|  \| 2.52E-4 \| \| --- \| --- \| | 2.371 | 3 | 67 | Yusenko Renal |
| Leukaemia | Hairy Cell Leukemia | 8.28E-11 | 3.708 | 2 | 336 | Basso Lymphoma |
|  | T-Cell Acute Lymphoblastic Leukemia | \|  \| 5.30E-7 \| \| --- \| --- \| | 2.927 | 5 | 127 | Andersson Leukimia |
| Liver | Liver Cell Dysplasia   \|  \| 0.007 \| \| --- \| --- \| | \|  \| 0.007 \| \| --- \| --- \| | 1.566 | 7 | 75 | Wurmbach Liver |
| Lung | Lung Adenocarcinoma | \|  \| 1.61E-4 \| \| --- \| --- \| | 1.748 | 4 | 73 | Garber Lung |
|  | Large Cell Lung Carcinoma | 0.005 | 2.607 | 7 | 73 | Garber Lung |
|  | Squamous Cell Lung Carcinoma | \|  \| 0.002 \| \| --- \| --- \| | 1.743 | 9 | 73 | Garber Lung |
|  | Lung Adenocarcinoma | 0.009 | 1.775 | 5 | 203 | Bhattacharjee Lung |
| Lymphoma | Angioimmunoblastic T-Cell Lymphoma | \|  \| 2.13E-14 \| \| --- \| --- \| | 5.646 | 1 | 60 | Piccaluga Lymphoma |
|  | Unspecified Peripheral T-Cell Lymphoma | 2.98E-15 | 5.036 | 2 | 60 | Piccaluga Lymphoma |
|  | Anaplastic Large Cell Lymphoma | \|  \| 3.59E-6 \| \| --- \| --- \| | 5.579 | 5 | 60 | Piccaluga Lymphoma |
|  | Centroblastic Lymphoma | 1.91E-9 | 3.007 | 6 | 336 | Basso Lymphoma |
|  | Burkitt’s Lymphoma | \|  \| 1.42E-5 \| \| --- \| --- \| | 2.328 | 9 | 336 | Basso Lymphoma |
|  | Primary Effusion Lymphoma | 2.12E-4 | 5.246 | 9 | 336 | Basso Lymphoma |
|  | Diffuse Large B-Cell Lymphoma | \|  \| 2.19E-12 \| \| --- \| --- \| | 2.306 | 7 | 136 | Compagno Lymphoma |
|  | Germinal Center B-Cell-Like Diffuse Large B-Cell Lymphoma | 3.00E-4 | 1.553 | 9 | 136 | Compagno Lymphoma |
|  | Activated B-Cell-Like Diffuse Large B-Cell Lymphoma | 3.91E-7 | 2.281 | 9 | 136 | Compagno Lymphoma |
| Myeloma | Smoldering Myeloma | \|  \| 8.75E-8 \| \| --- \| --- \| | 4.979 | 3 | 78 | Zhan Myeloma 3 |
| Prostate | Prostate Carcinoma Epithelia | 2.16E-6 | 4.672 | 2 | 101 | Tomlins Prostate |
|  | Prostatic Intraepithelial Neoplasia Epithelia | 2.36E-5 | 5.431 | 2 | 101 | Tomlins Prostate |
| Sarcoma | Round Cell Liposarcoma | 0.001 | 1.701 | 7 | 54 | Detwiller Sarcoma |

**Table S2.** Significantly different expression of *JMJD10* in tumor TCGA by TIMER

| **Tumor** | **Normal** | **p-value** |
| --- | --- | --- |
| BLCA.Tumor | BLCA.Normal | 0.070957917 |
| BRCA.Tumor | BRCA.Normal | 1.45126E-09 |
| CHOL.Tumor | CHOL.Normal | 2.46456E-06 |
| COAD.Tumor | COAD.Normal | 1.95966E-15 |
| ESCA.Tumor | ESCA.Normal | 6.60174E-05 |
| HNSC-HPVpos.Tumor | HNSC-HPVneg.Tumor | 0.082712594 |
| HNSC.Tumor | HNSC.Normal | 0.107933669 |
| KICH.Tumor | KICH.Normal | 0.192730529 |
| KIRC.Tumor | KIRC.Normal | 8.88154E-37 |
| KIRP.Tumor | KIRP.Normal | 1.18571E-16 |
| LIHC.Tumor | LIHC.Normal | 4.36499E-11 |
| LUAD.Tumor | LUAD.Normal | 9.82509E-18 |
| LUSC.Tumor | LUSC.Normal | 1.70759E-20 |
| PRAD.Tumor | PRAD.Normal | 0.001306791 |
| READ.Tumor | READ.Normal | 0.000358048 |
| SKCM.Tumor | SKCM.Metastasis | 0.00027265 |
| STAD.Tumor | STAD.Normal | 3.5826E-06 |
| THCA.Tumor | THCA.Normal | 1.51622E-17 |
| UCEC.Tumor | UCEC.Normal | 0.001462337 |
