# Supplementary material for: Molecular Signatures of JMJD10/MINA53 in Gastric Cancer
Source: Cancers (Basel). 2020 May 2;12(5):1141. doi: 10.3390/cancers12051141 (PMC7281541; doi:10.3390/cancers12051141)
Supplement: Supplementary file 1 [file cancers-12-01141-s001.zip › cancers-773947 supplementary/Figure S1-Whole immunoblot results.pdf]

Supplementary Figure

Whole blot images of immunoblotting results

Fig. 3 A

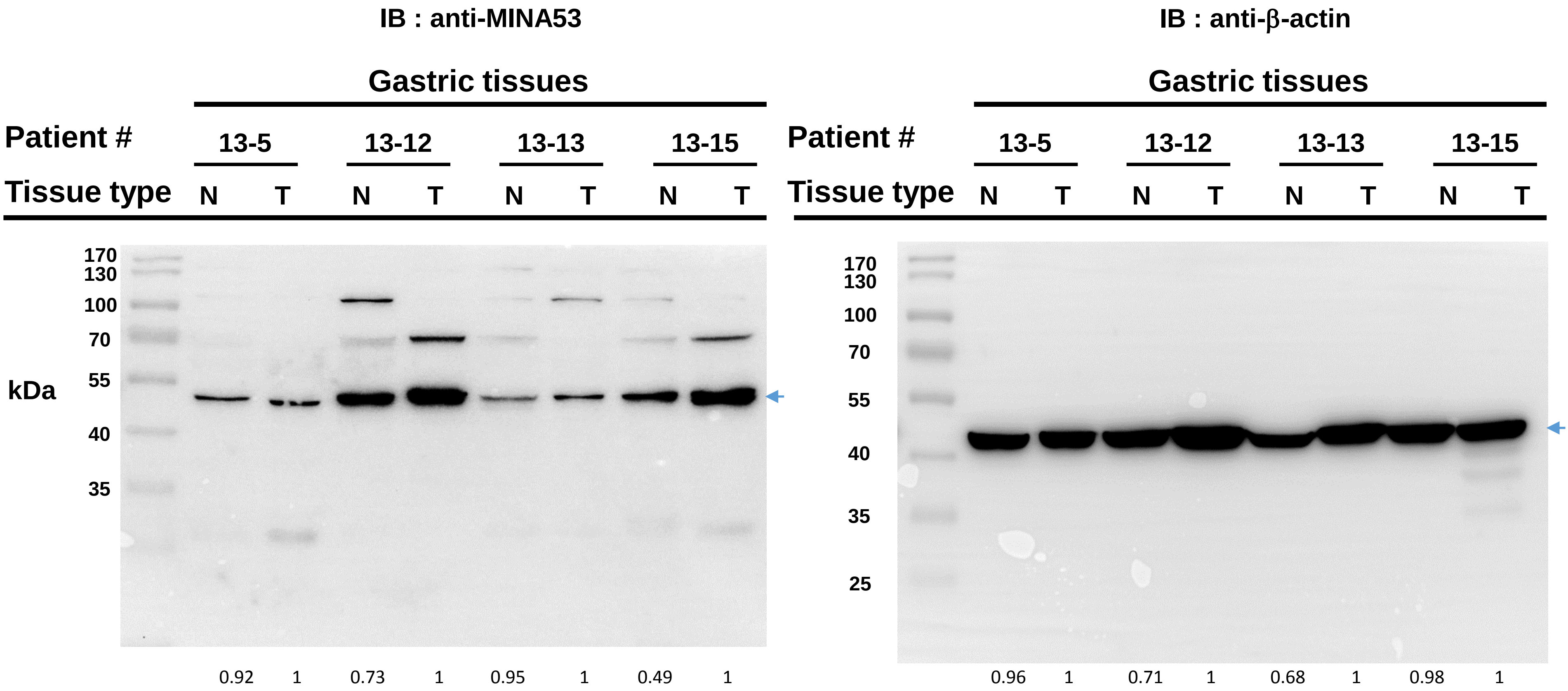
IB : anti-MINA53
Gastric tissues
Patient #
13-5
13-12
13-13
13-15
Tissue type
N T N T N T N T
kDa
170
130
100
70
55
40
35
0.92 1 0.73 1 0.95 1 0.49 1
IB : anti-β-actin
Gastric tissues
Patient #
13-5
13-12
13-13
13-15
Tissue type
N T N T N T N T
170
130
100
70
55
40
35
25
0.96 1 0.71 1 0.68 1 0.98 1

Fig. 3 A

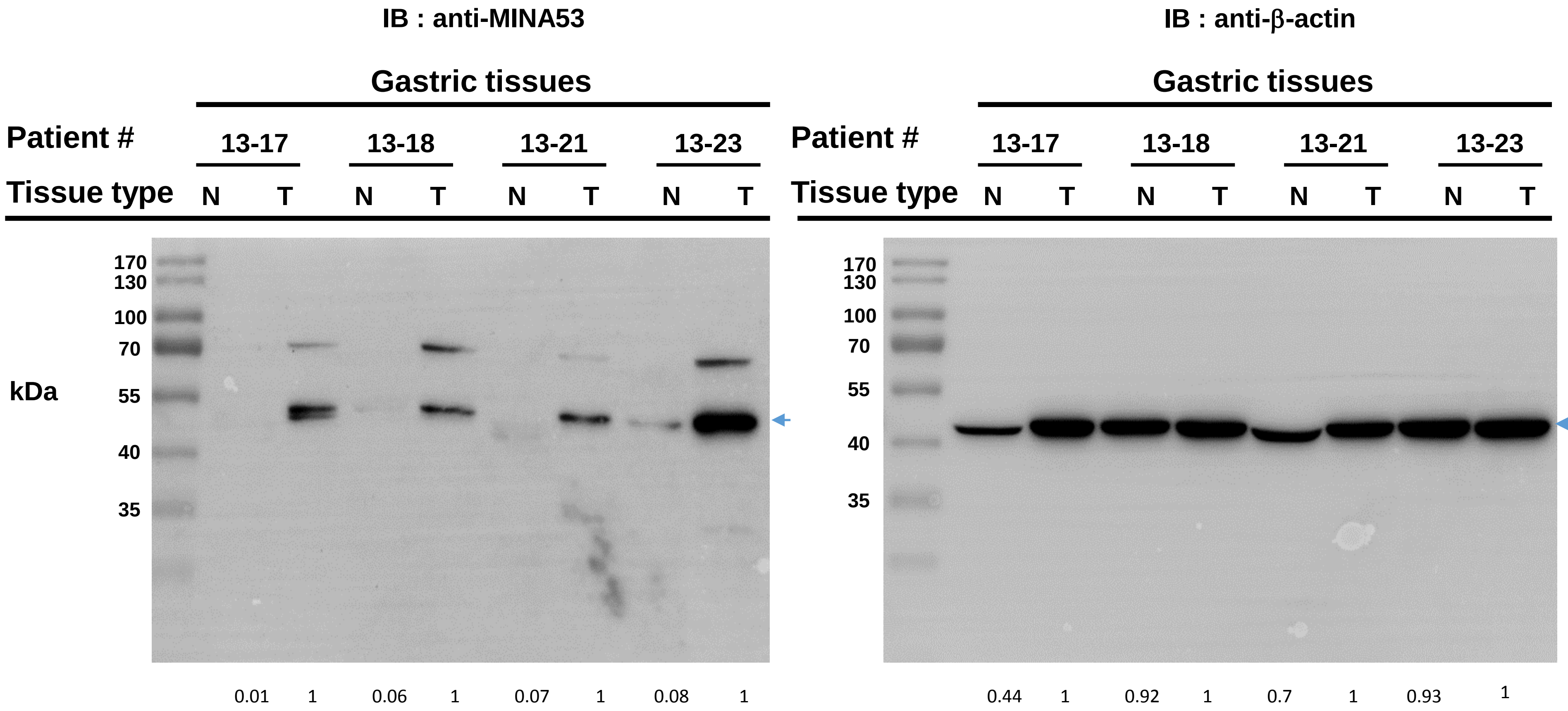
IB : anti-MINA53
Gastric tissues
Patient #
13-17
13-18
13-21
13-23
Tissue type
N T N T N T N T
170
130
100
70
55
40
35
kDa
0.01 1 0.06 1 0.07 1 0.08 1
IB : anti-β-actin
Gastric tissues
Patient #
13-17
13-18
13-21
13-23
Tissue type
N T N T N T N T
170
130
100
70
55
40
35
0.44 1 0.92 1 0.7 1 0.93 1

Fig. 3 A

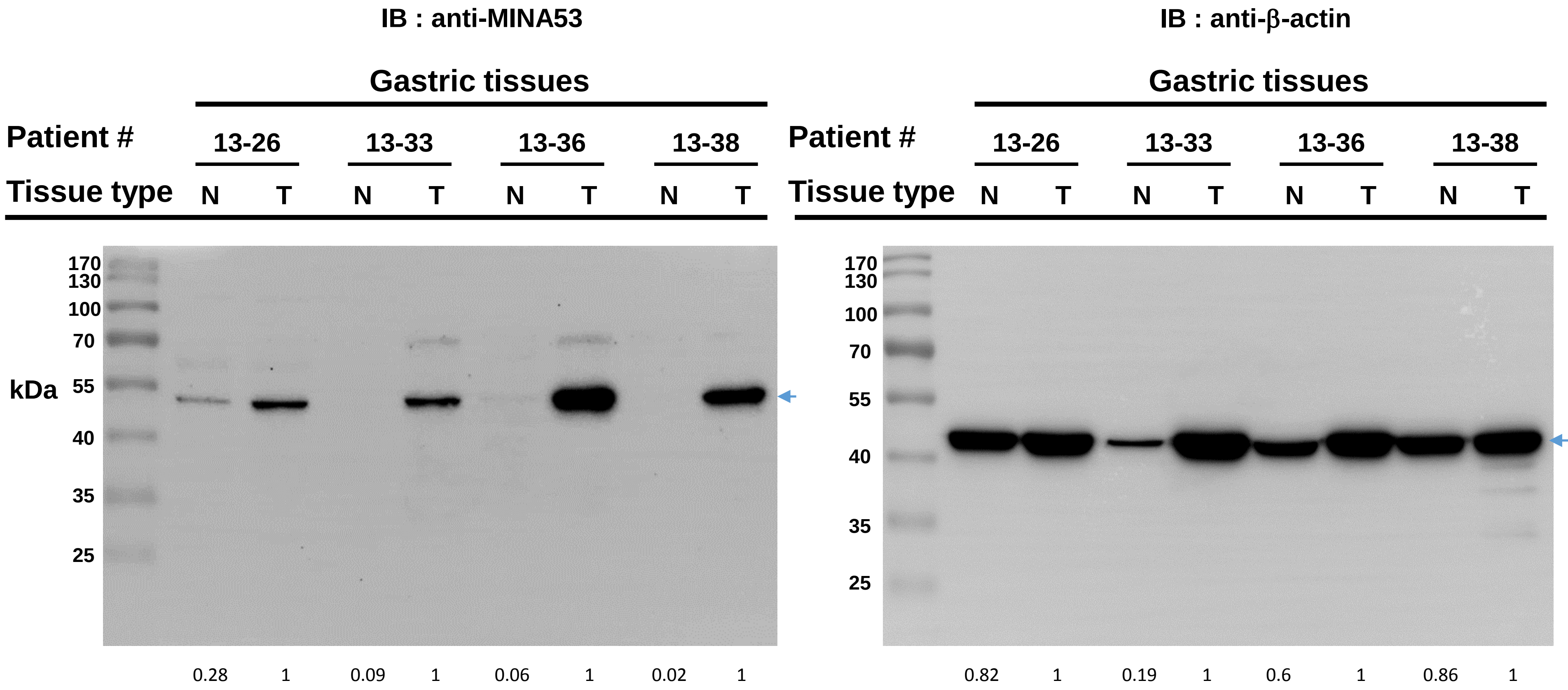

IB : anti-MINA53
Gastric tissues
Patient #
13-26
13-33
13-36
13-38
Tissue type
N T N T N T N T
kDa
170
130
100
70
55
40
35
25
0.28 1 0.09 1 0.06 1 0.02 1
IB : anti-β-actin
Gastric tissues
Patient #
13-26
13-33
13-36
13-38
Tissue type
N T N T N T N T
170
130
100
70
55
40
35
25
0.82 1 0.19 1 0.6 1 0.86 1

Fig. 3 A

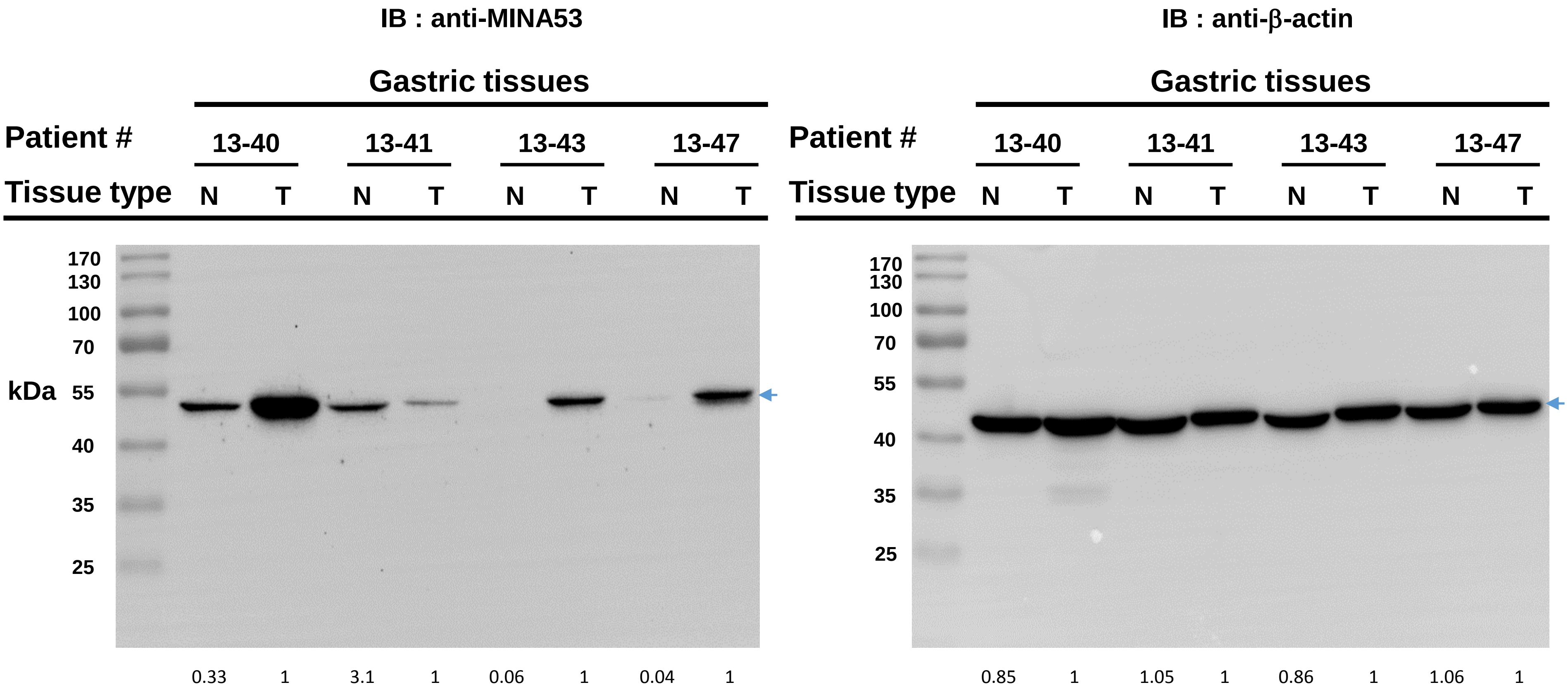
IB : anti-MINA53
Gastric tissues
Patient #
13-40
13-41
13-43
13-47
Tissue type
N T N T N T N T
170
130
100
70
kDa
55
40
35
25
0.33 1 3.1 1 0.06 1 0.04 1
IB : anti-β-actin
Gastric tissues
Patient #
13-40
13-41
13-43
13-47
Tissue type
N T N T N T N T
170
130
100
70
55
40
35
25
0.85 1 1.05 1 0.86 1 1.06 1

Fig. 3 A

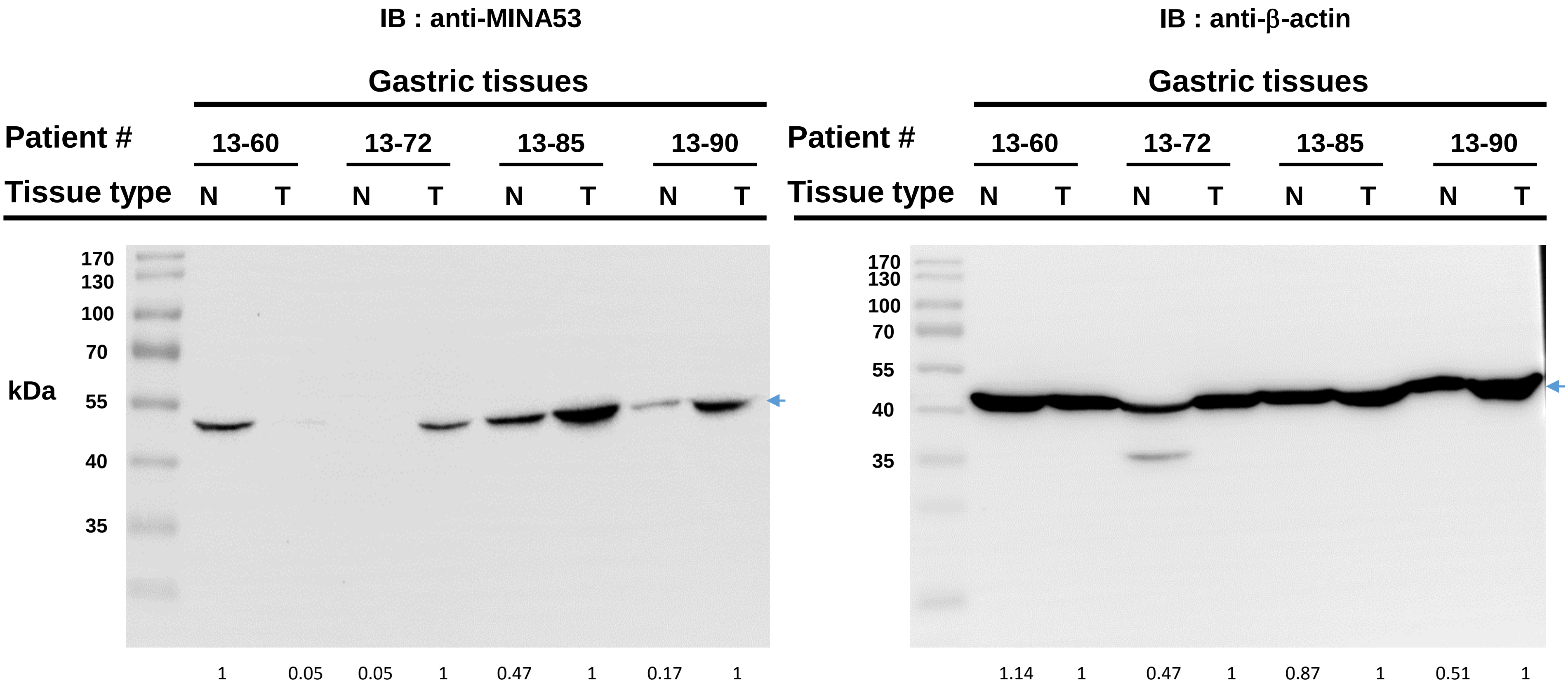
IB : anti-MINA53
Gastric tissues
Patient #
13-60
13-72
13-85
13-90
Tissue type
N T N T N T N T
kDa
170
130
100
70
55
40
35
1 0.05 0.05 1 0.47 1 0.17 1
IB : anti-β-actin
Gastric tissues
Patient #
13-60
13-72
13-85
13-90
Tissue type
N T N T N T N T
170
130
100
70
55
40
35
1.14 1 0.47 1 0.87 1 0.51 1

Fig. 3 A

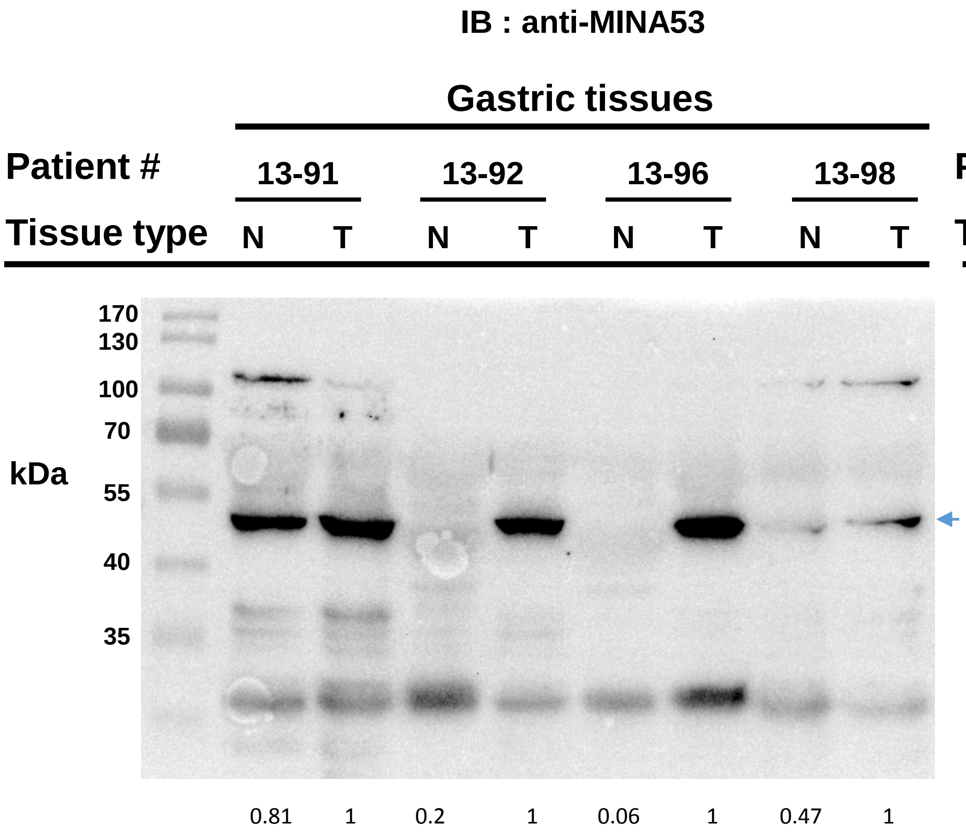

IB : anti-MINA53
Gastric tissues
Patient #
13-91
13-92
13-96
13-98
Tissue type
N T N T N T N T
kDa
170
130
100
70
55
40
35
0.81 1 0.2 1 0.06 1 0.47 1

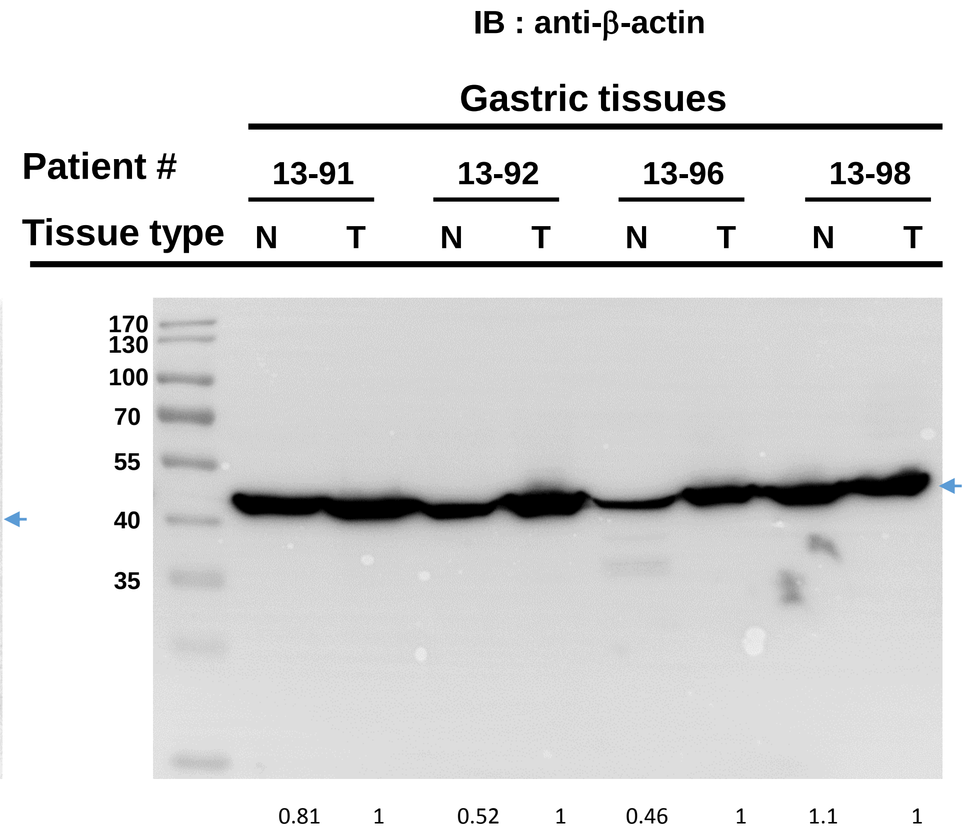

IB : anti-β-actin
Gastric tissues
Patient #
13-91
13-92
13-96
13-98
Tissue type
N T N T N T N T
170
130
100
70
55
40
35
0.81 1 0.52 1 0.46 1 1.1 1

Fig. 3 A

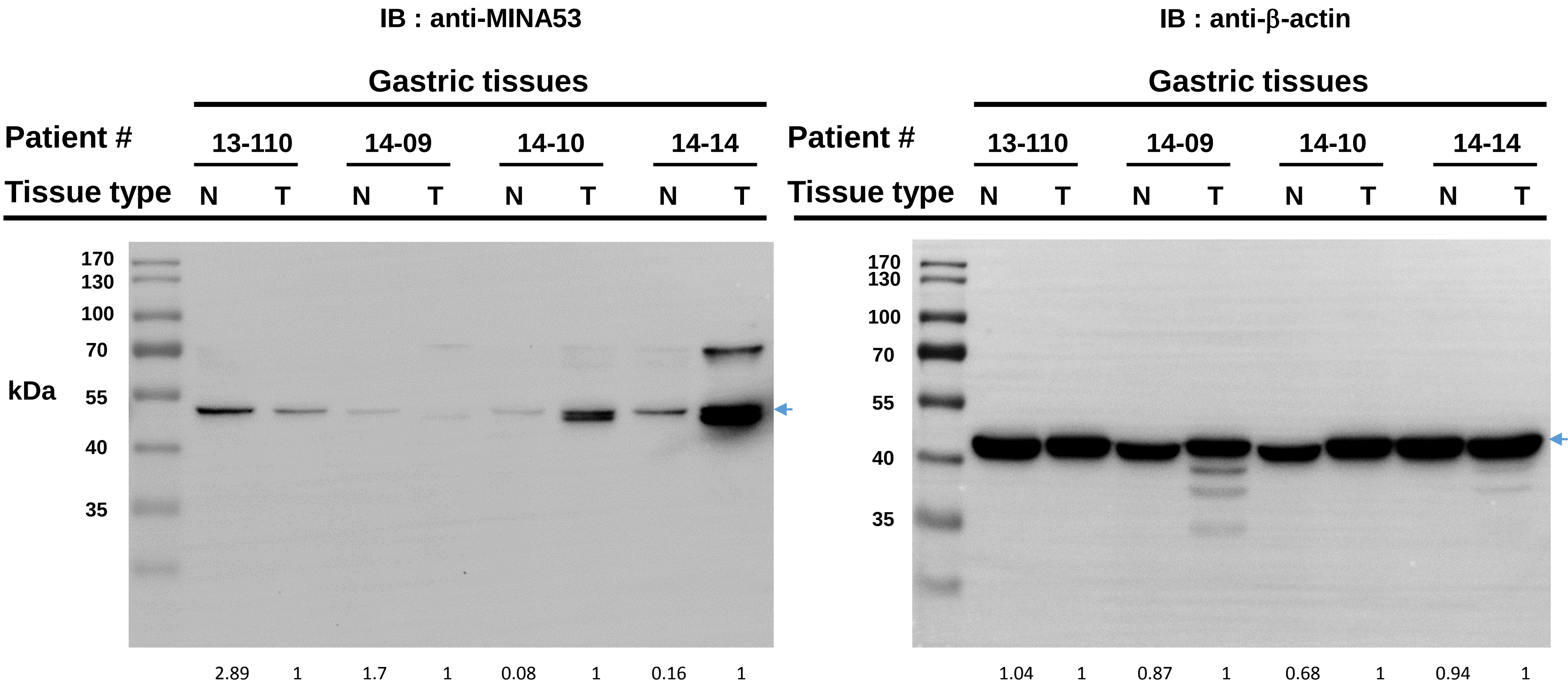
IB : anti-MINA53
Gastric tissues
Patient #
13-110
14-09
14-10
14-14
Tissue type
N T N T N T N T
kDa
170
130
100
70
55
40
35
2.89 1 1.7 1 0.08 1 0.16 1
IB : anti-β-actin
Gastric tissues
Patient #
13-110
14-09
14-10
14-14
Tissue type
N T N T N T N T
170
130
100
70
55
40
35
1.04 1 0.87 1 0.68 1 0.94 1

Fig. 3 A

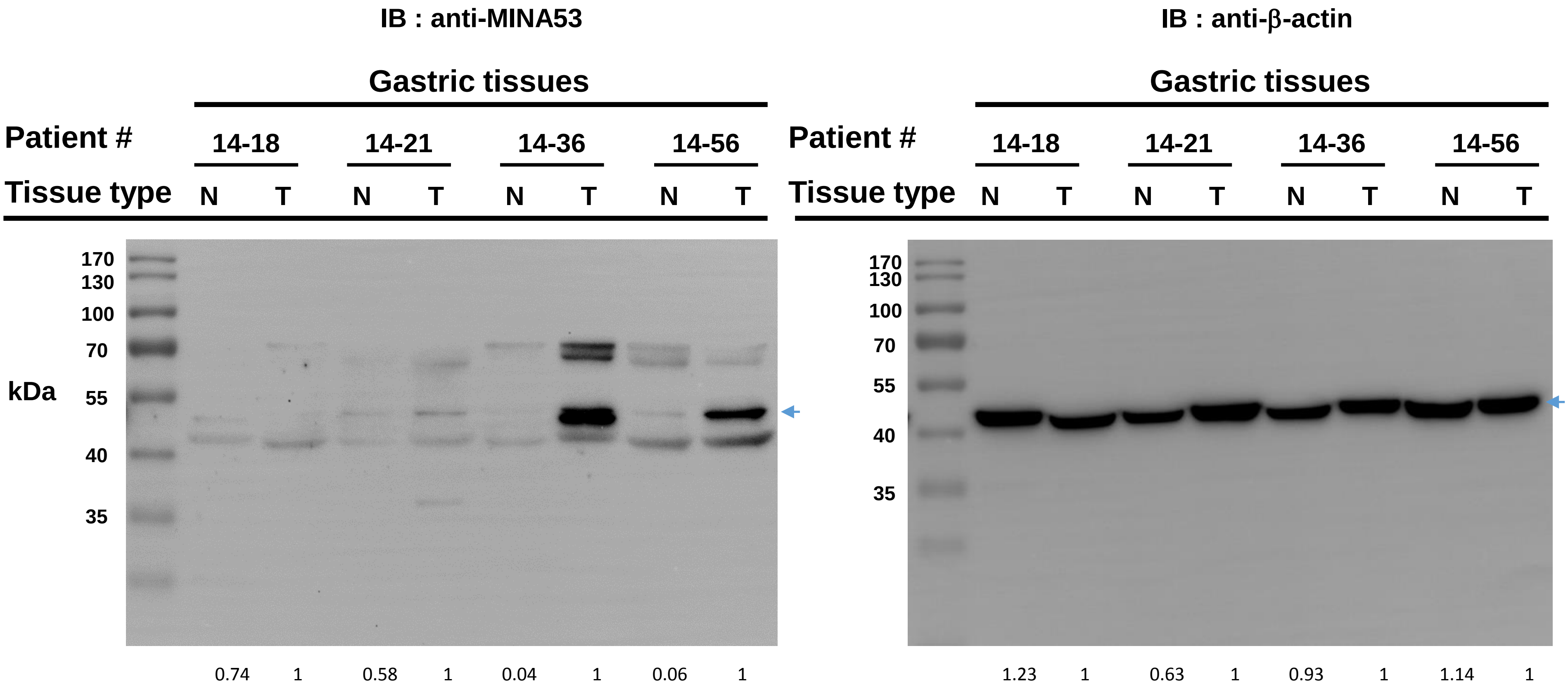

IB : anti-MINA53
Gastric tissues
Patient #
14-18
14-21
14-36
14-56
Tissue type
N T N T N T N T
170
130
100
70
kDa
55
40
35
0.74 1 0.58 1 0.04 1 0.06 1
IB : anti-β-actin
Gastric tissues
Patient #
14-18
14-21
14-36
14-56
Tissue type
N T N T N T N T
170
130
100
70
55
40
35
1.23 1 0.63 1 0.93 1 1.14 1

Fig. 3 A

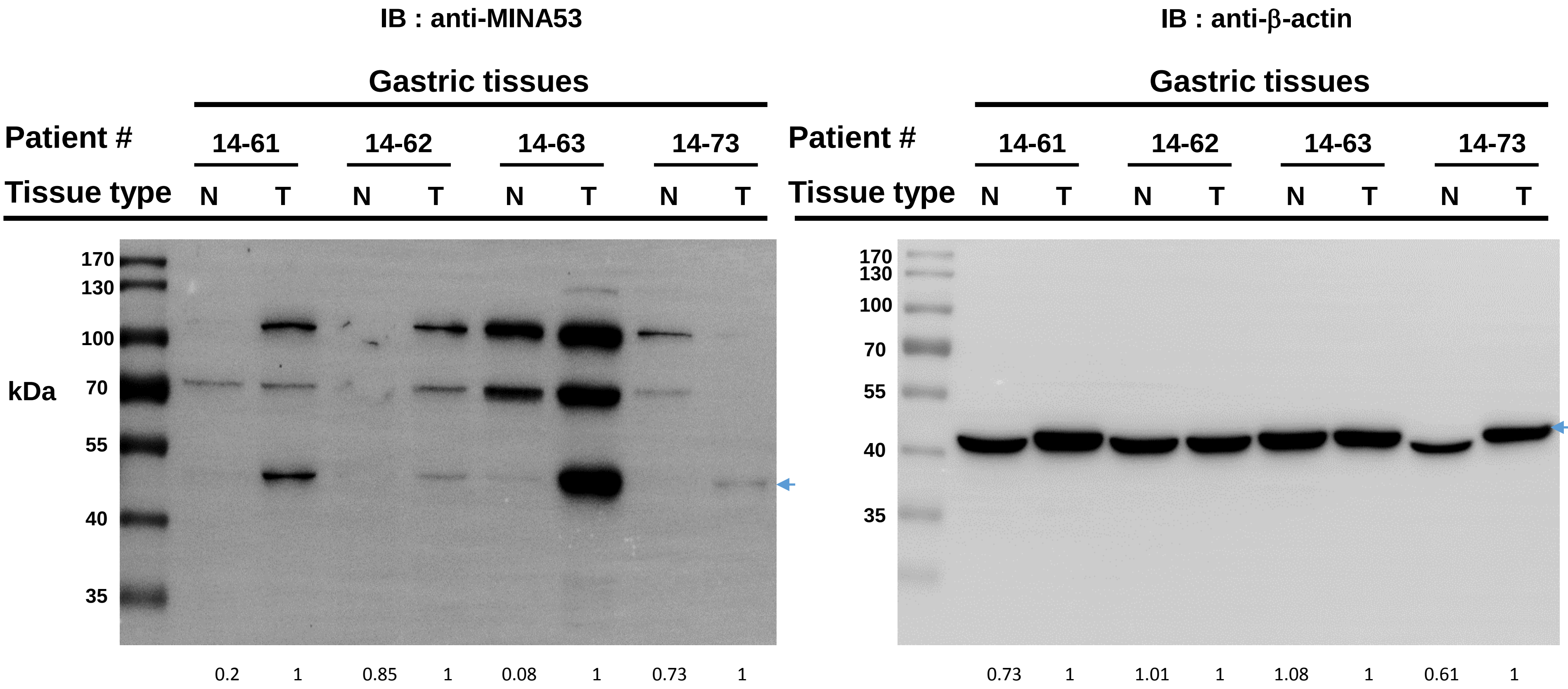
IB : anti-MINA53
Gastric tissues
Patient #
14-61
14-62
14-63
14-73
Tissue type
N
T
N
T
N
T
N
T
kDa
170
130
100
70
55
40
35
0.2
1
0.85
1
0.08
1
0.73
1
IB : anti-β-actin
Gastric tissues
Patient #
14-61
14-62
14-63
14-73
Tissue type
N
T
N
T
N
T
N
T
170
130
100
70
55
40
35
0.73
1
1.01
1
1.08
1
0.61
1

Fig. 3 A

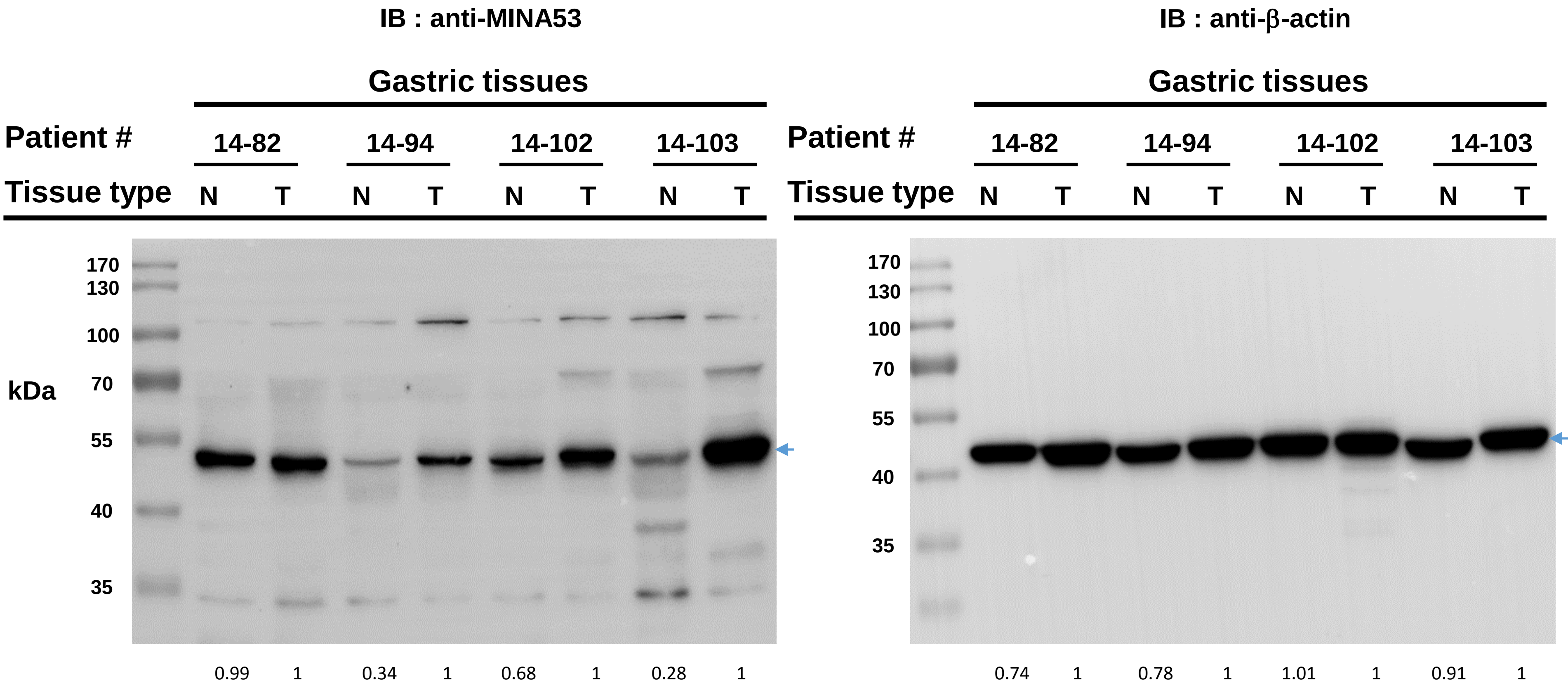
IB : anti-MINA53
Gastric tissues
Patient #
14-82
14-94
14-102
14-103
Tissue type
N
T
N
T
N
T
N
T
kDa
170
130
100
70
55
40
35
0.99
1
0.34
1
0.68
1
0.28
1
IB : anti-β-actin
Gastric tissues
Patient #
14-82
14-94
14-102
14-103
Tissue type
N
T
N
T
N
T
N
T
170
130
100
70
55
40
35
0.74
1
0.78
1
1.01
1
0.91
1

Fig. 3 A

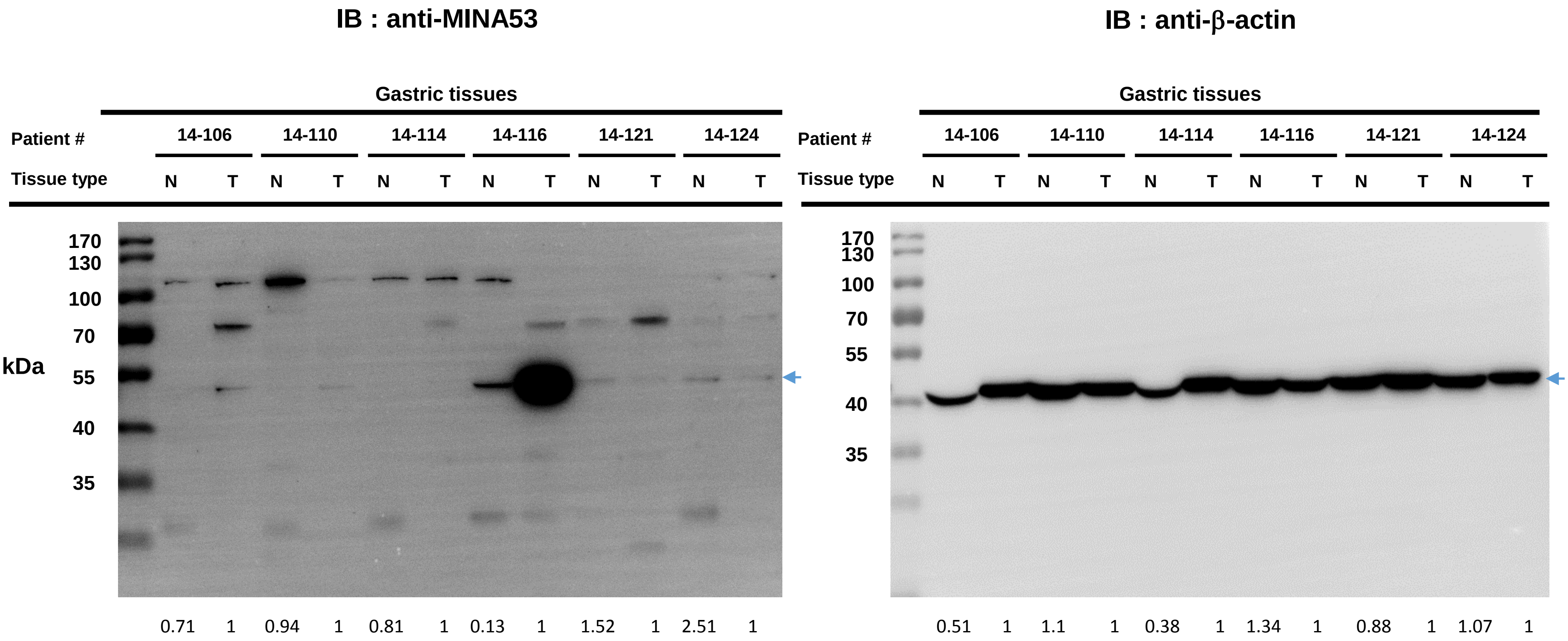
IB : anti-MINA53
IB : anti-β-actin
Gastric tissues
Patient #
14-106
14-110
14-114
14-116
14-121
14-124
Tissue type
N
T
kDa
170
130
100
70
55
40
35
0.71 1 0.94 1 0.81 1 0.13 1 1.52 1 2.51 1
0.51 1 1.1 1 0.38 1 1.34 1 0.88 1 1.07 1

Fig. 3 A

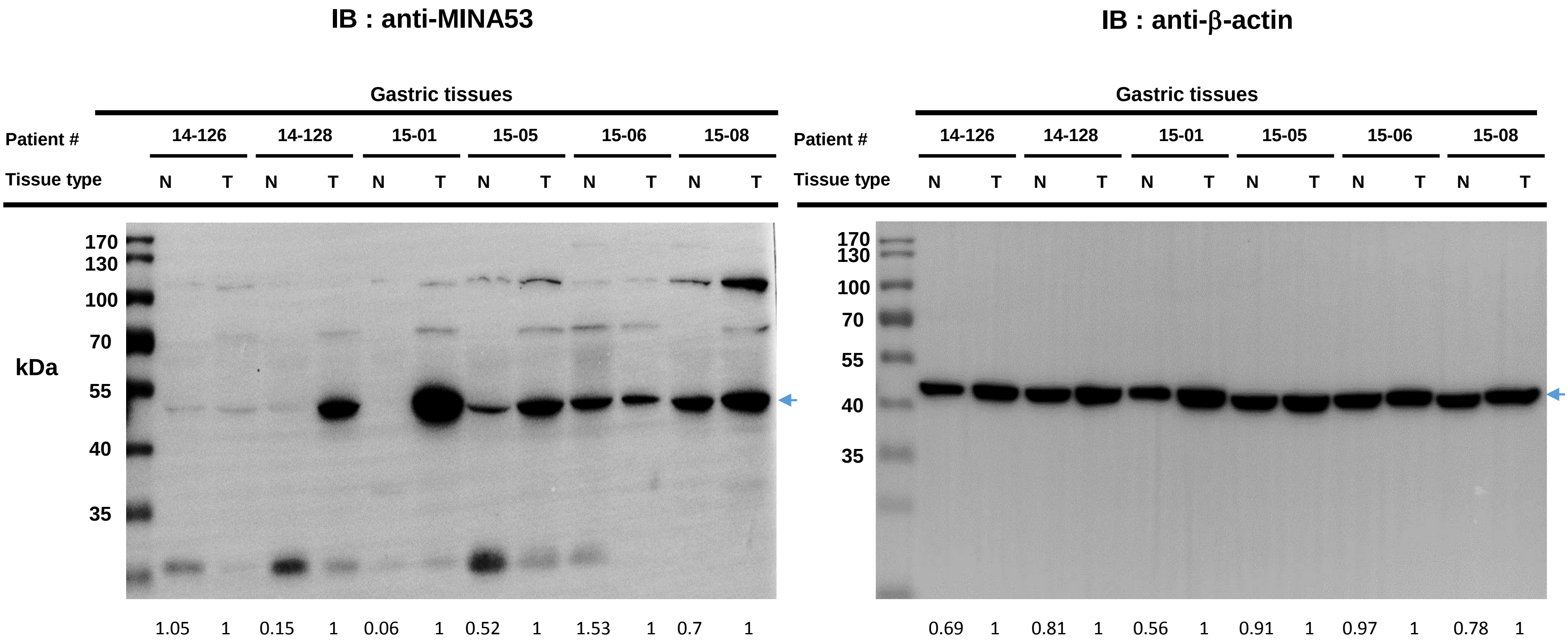
IB : anti-MINA53
IB : anti-β-actin
Gastric tissues
Patient #
14-126
14-128
15-01
15-05
15-06
15-08
Tissue type
N T N T N T N T N T N T
kDa
170
130
100
70
55
40
35
1.05 1 0.15 1 0.06 1 0.52 1 1.53 1 0.7 1
0.69 1 0.81 1 0.56 1 0.91 1 0.97 1 0.78 1

Fig. 4 A

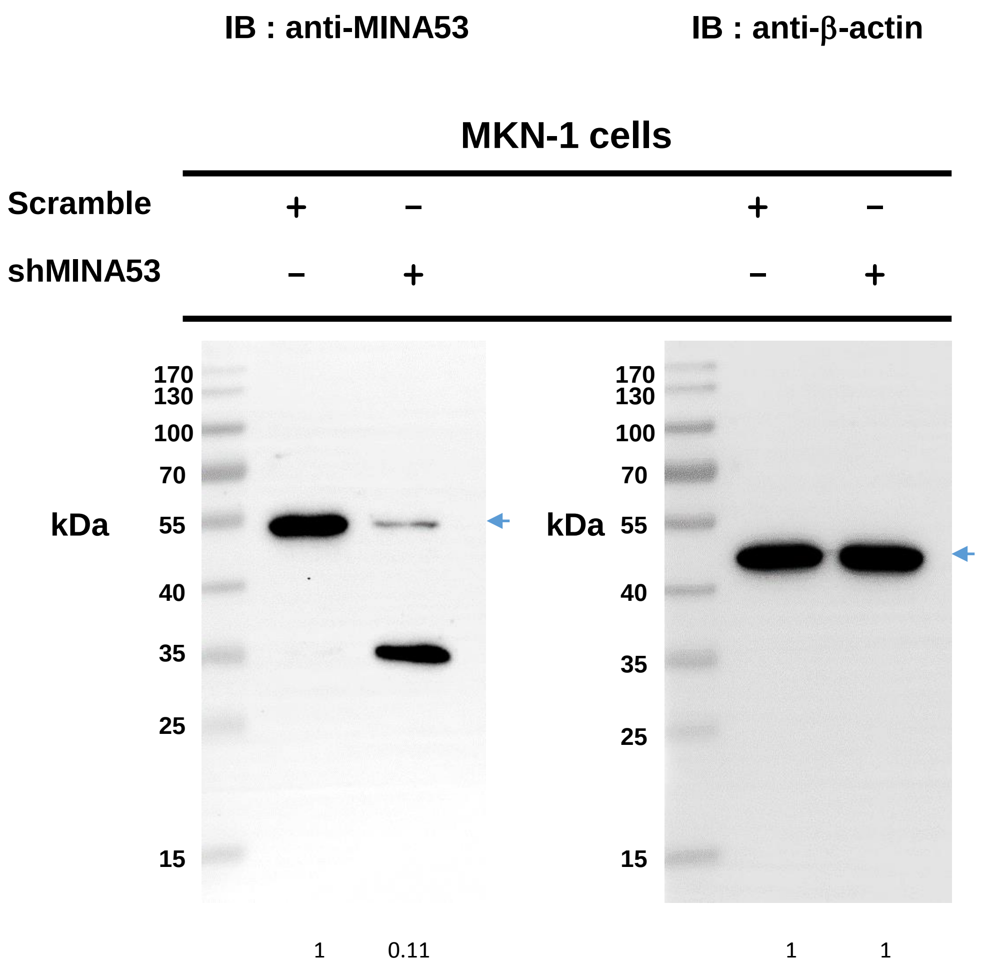

IB : anti-MINA53
IB : anti-β-actin
MKN-1 cells
Scramble
shMINA53
+
–
–
+
+
–
–
+
kDa
170
130
100
70
55
40
35
25
15
kDa
170
130
100
70
55
40
35
25
15
1
0.11
1
1

Fig. 5 B

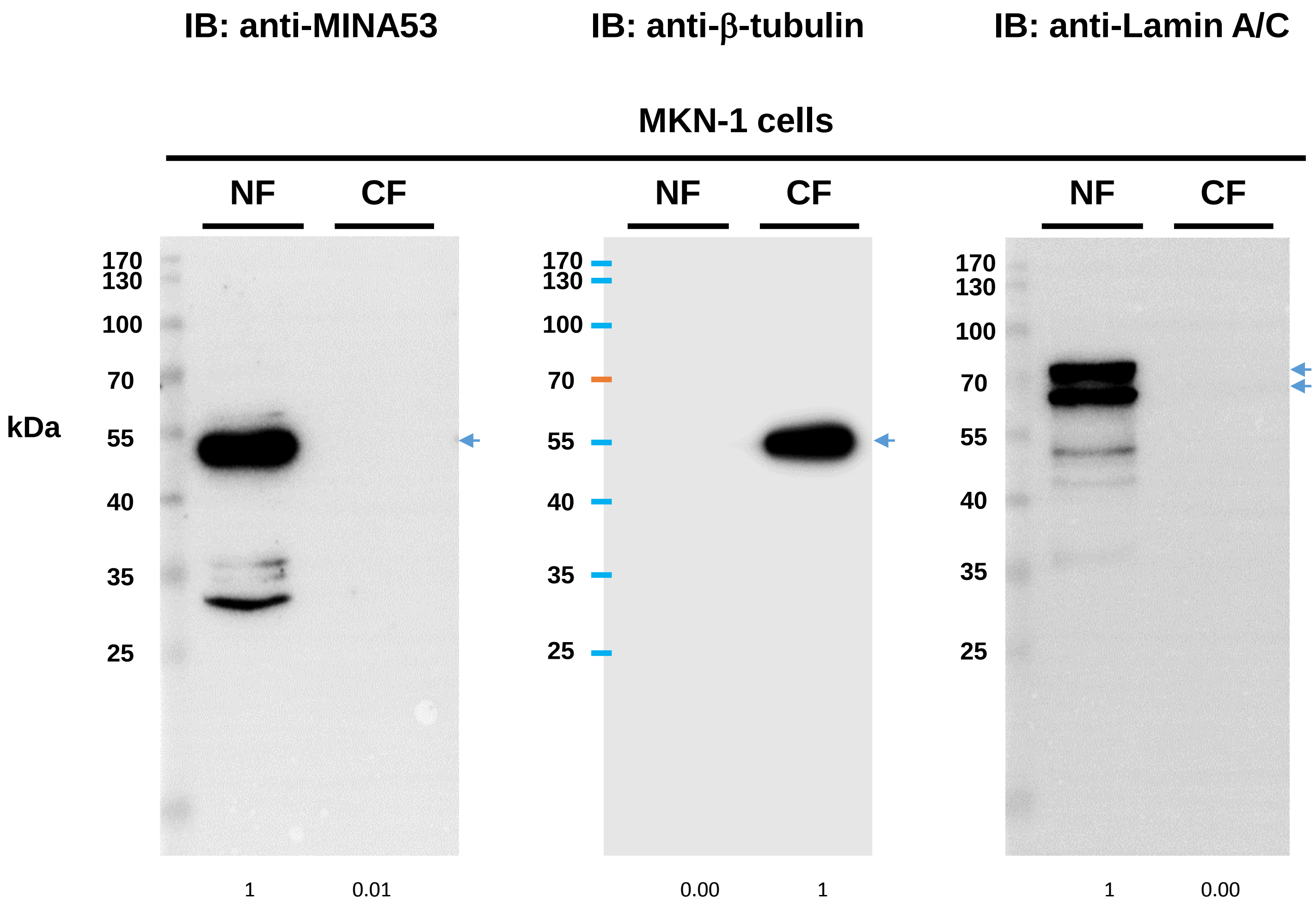
IB: anti-MINA53
IB: anti-β-tubulin
IB: anti-Lamin A/C
MKN-1 cells
NF
CF
NF
CF
NF
CF
kDa
170
130
100
70
55
40
35
25
170
130
100
70
55
40
35
25
170
130
100
70
55
40
35
25
1
0.01
0.00
1
1
0.00

Fig. 5 C (left panel)

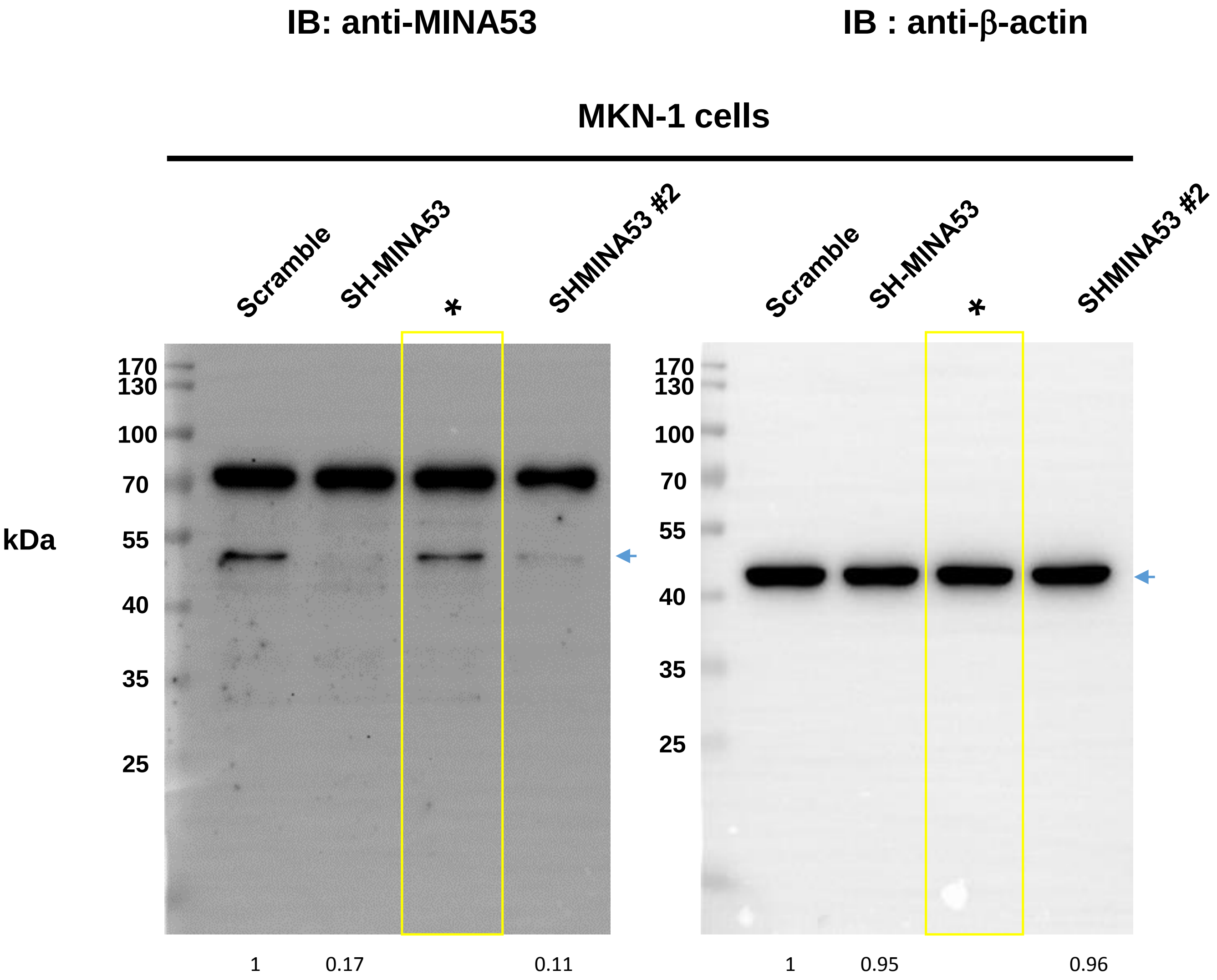

* This lane was cut due to low knockdown level

Fig. 5 C (right panel)

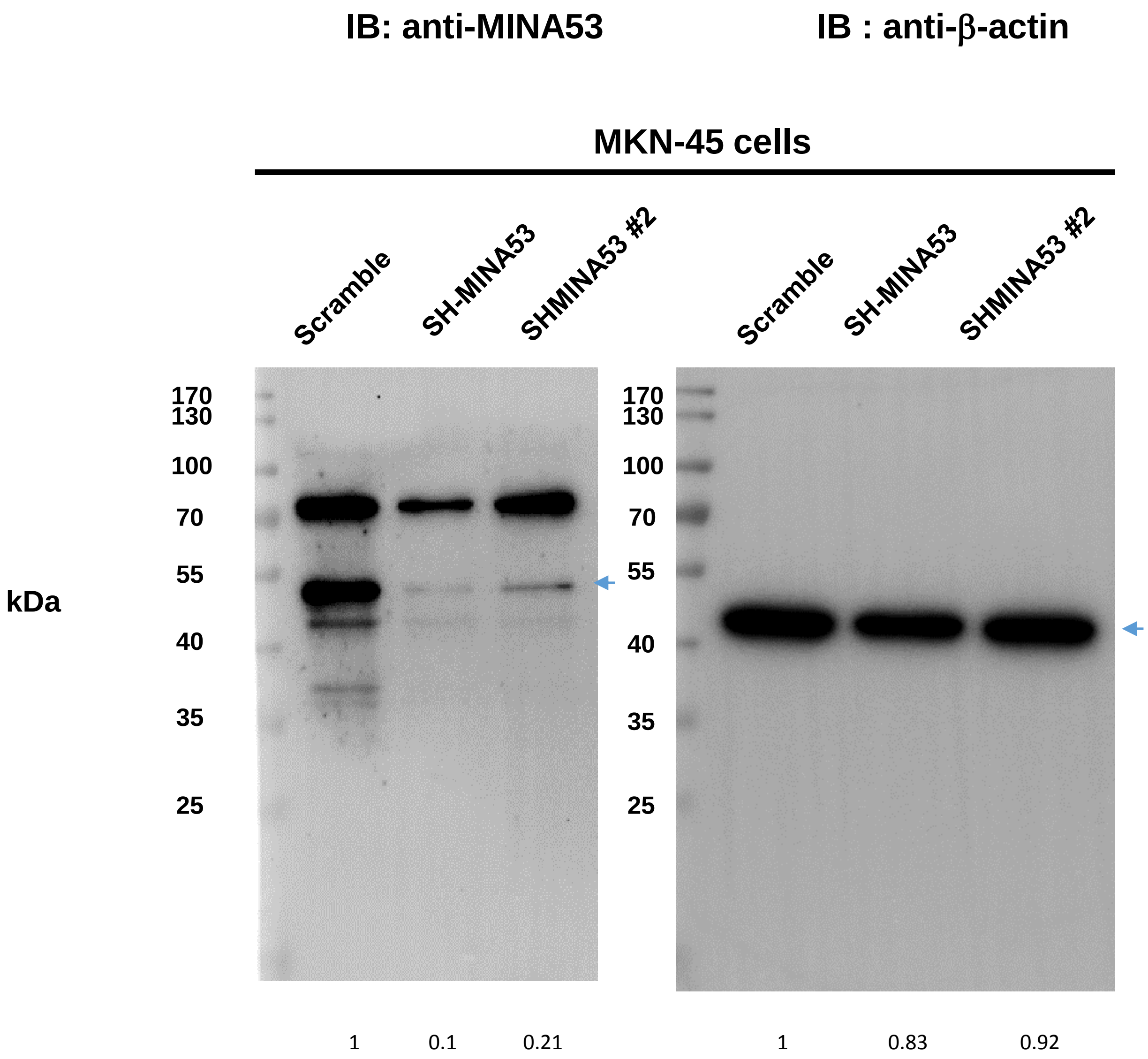

Fig. 5 C

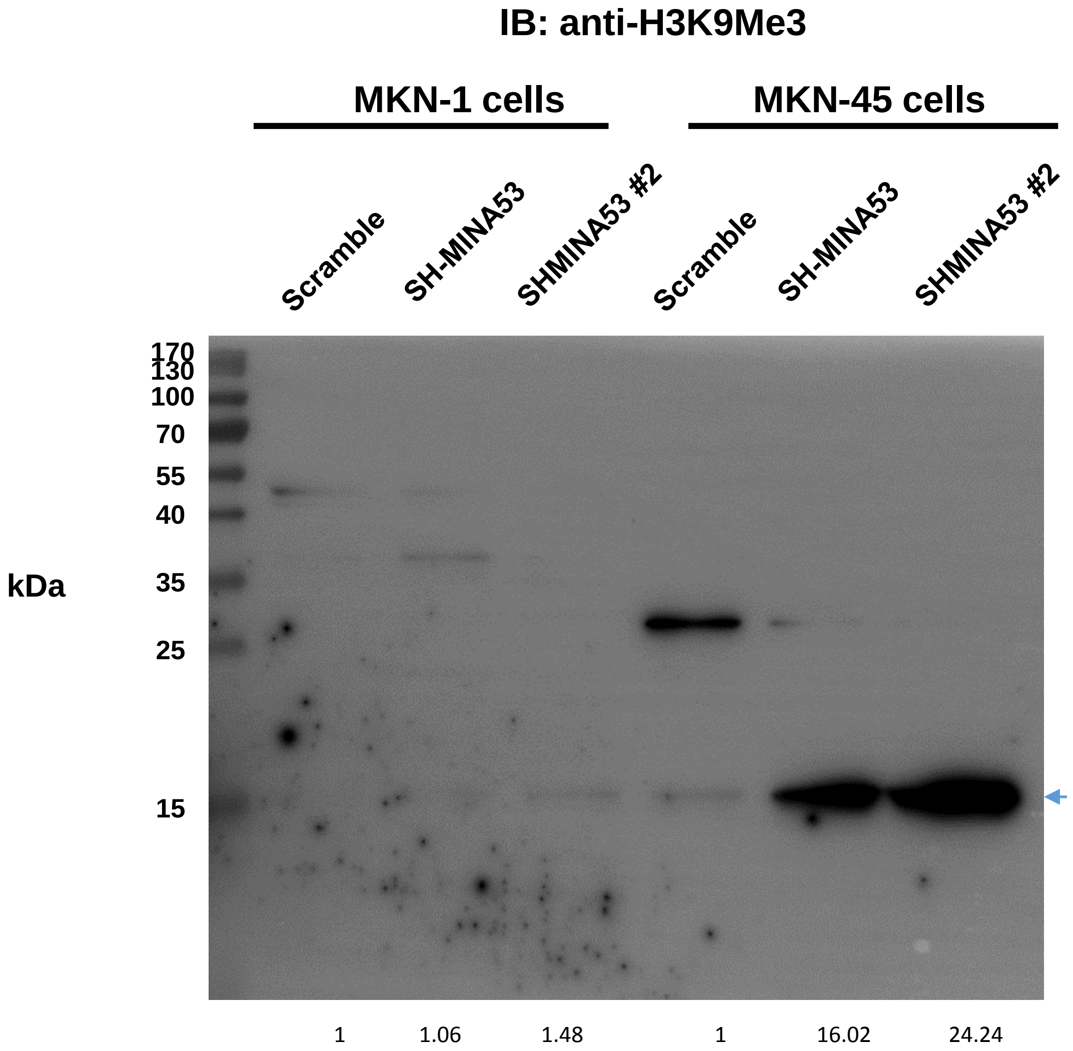
IB: anti-H3K9Me3
MKN-1 cells
MKN-45 cells
Scramble
SH-MINA53
SHMINA53 #2
Scramble
SH-MINA53
SHMINA53 #2
170
130
100
70
55
40
kDa
35
25
15
1
1.06
1.48
1
16.02
24.24

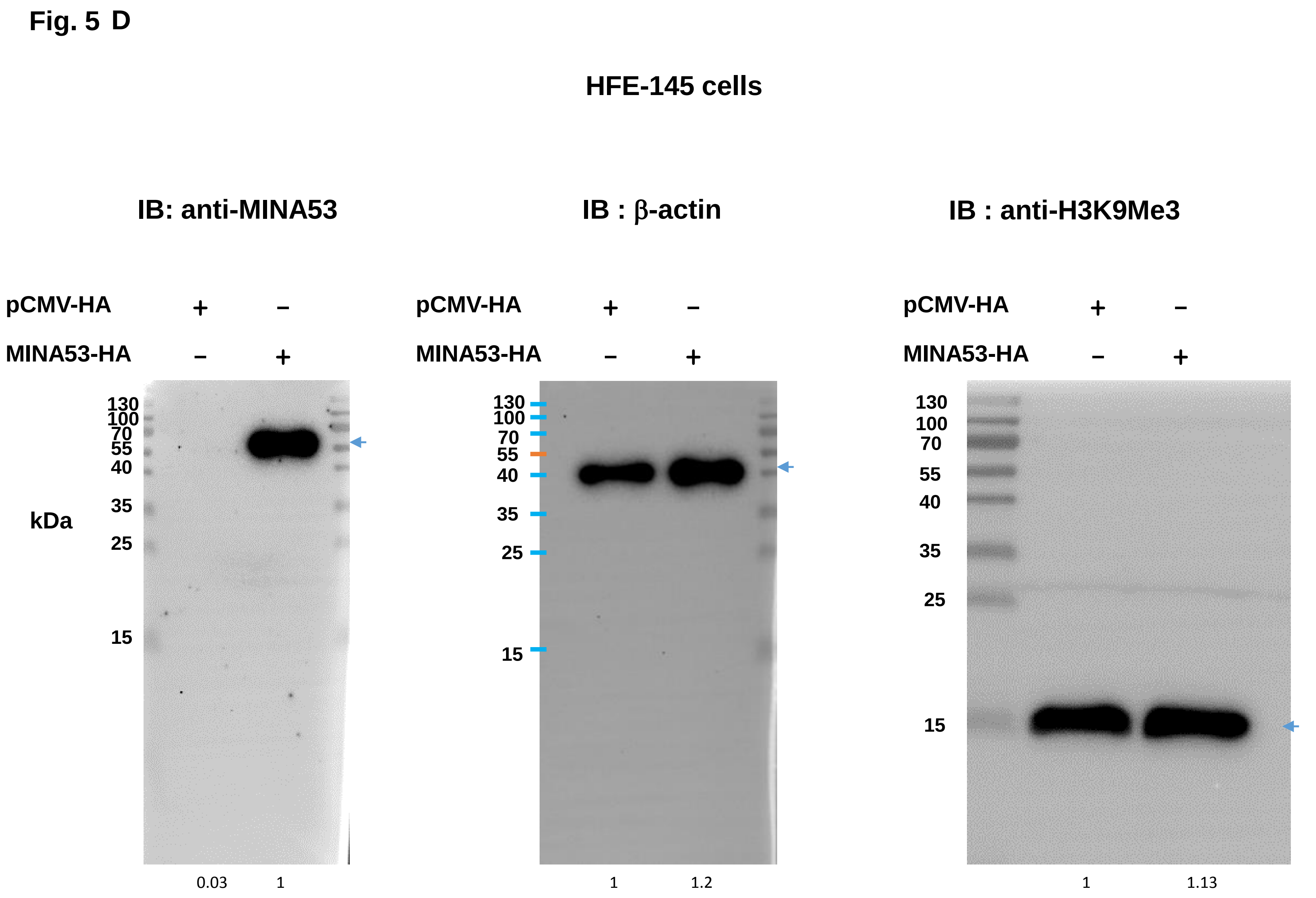
Fig. 5 D
HFE-145 cells
IB: anti-MINA53
IB : β-actin
IB : anti-H3K9Me3
pCMV-HA + –
MINA53-HA – +
130
100
70
55
40
35
25
15
kDa
0.03 1
1 1.2
1 1.13

Fig. 7 B

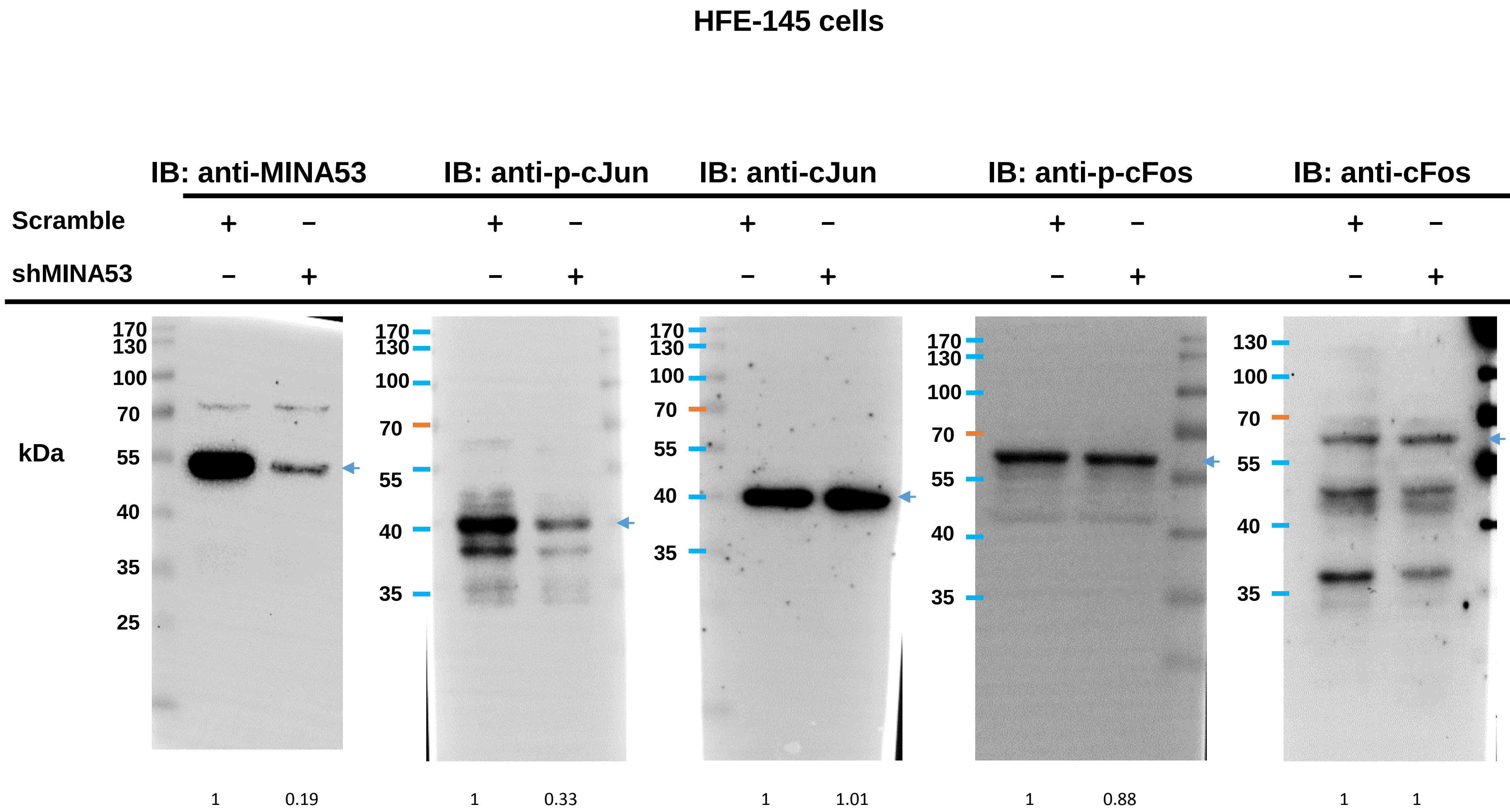
HFE-145 cells
IB: anti-MINA53
IB: anti-p-cJun
IB: anti-cJun
IB: anti-p-cFos
IB: anti-cFos
Scramble + − + − + − + − + −
shMINA53 − + − + − + − + − +
kDa
170
130
100
70
55
40
35
25
1 0.19
1 0.33
1 1.01
1 0.88
1 1

Fig. 7 B

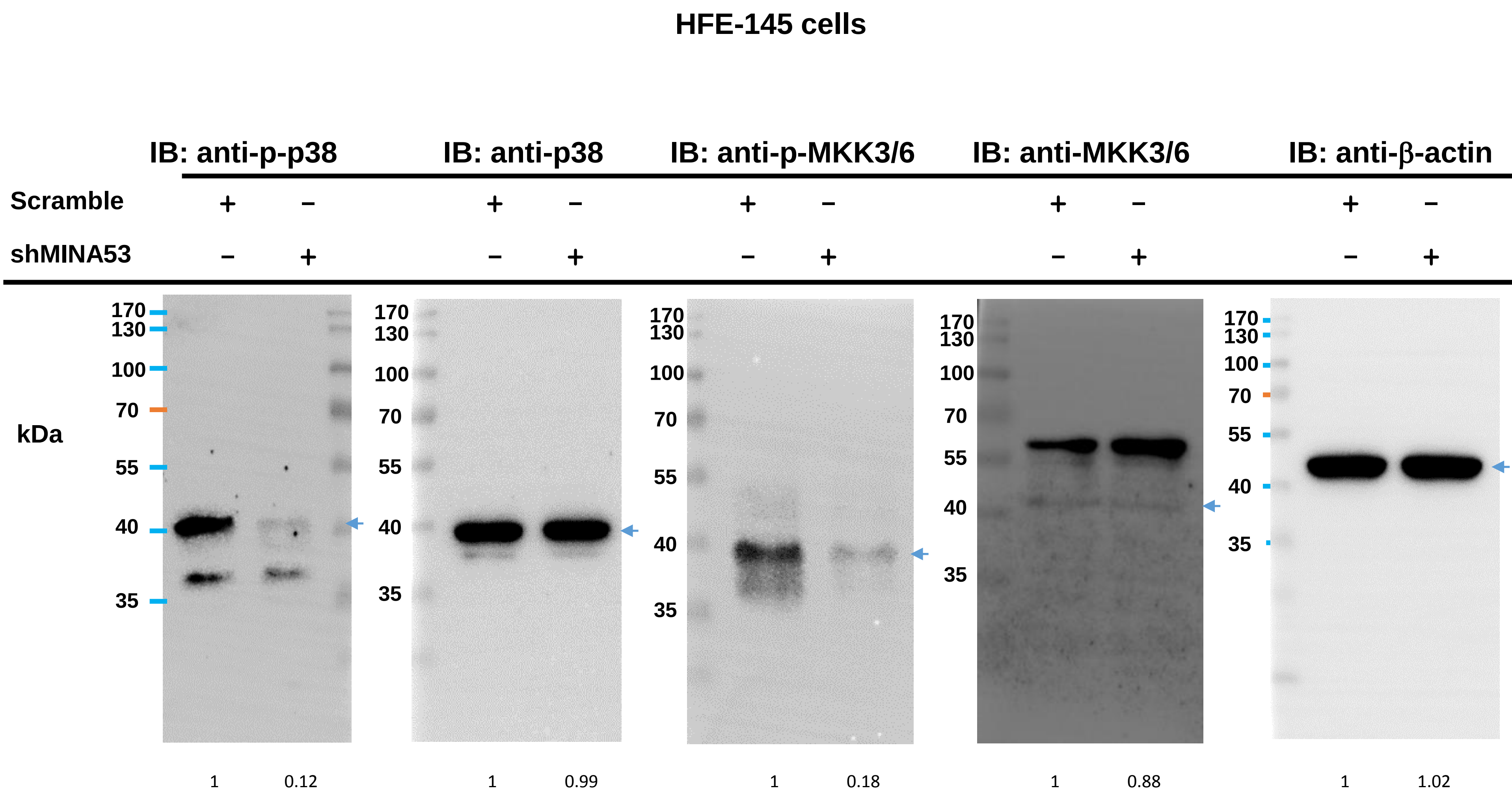
HFE-145 cells
IB: anti-p-p38
IB: anti-p38
IB: anti-p-MKK3/6
IB: anti-MKK3/6
IB: anti-β-actin
Scramble + − + − + − + − + −
shMINA53 − + − + − + − + − +
kDa
170
130
100
70
55
40
35
1
0.12
1
0.99
1
0.18
1
0.88
1
1.02
