# Supplementary material for: Molecular Signatures of JMJD10/MINA53 in Gastric Cancer
Source: Cancers (Basel). 2020 May 2;12(5):1141. doi: 10.3390/cancers12051141 (PMC7281541; doi:10.3390/cancers12051141)
Supplement: Supplementary file 1 [file cancers-12-01141-s001.zip › cancers-773947 supplementary/Supplementary file 2 gene alteration frequency.pdf]

Study ID	Sample ID	Patient ID	Altered	RIOX2
stad_tcga_pan_can_atlas_2018	TCGA-BR-4368-01	TCGA-BR-4368	1	HIGH
stad_tcga_pan_can_atlas_2018	TCGA-BR-6452-01	TCGA-BR-6452	1	I150F
stad_tcga_pan_can_atlas_2018	TCGA-BR-6707-01	TCGA-BR-6707	1	LOW
stad_tcga_pan_can_atlas_2018	TCGA-BR-8078-01	TCGA-BR-8078	1	S91G
stad_tcga_pan_can_atlas_2018	TCGA-BR-8295-01	TCGA-BR-8295	1	HIGH
stad_tcga_pan_can_atlas_2018	TCGA-BR-8296-01	TCGA-BR-8296	1	HIGH
stad_tcga_pan_can_atlas_2018	TCGA-BR-8361-01	TCGA-BR-8361	1	HIGH
stad_tcga_pan_can_atlas_2018	TCGA-BR-8363-01	TCGA-BR-8363	1	C105R
stad_tcga_pan_can_atlas_2018	TCGA-BR-8369-01	TCGA-BR-8369	1	HIGH
stad_tcga_pan_can_atlas_2018	TCGA-BR-8687-01	TCGA-BR-8687	1	HIGH
stad_tcga_pan_can_atlas_2018	TCGA-BR-A4IV-01	TCGA-BR-A4IV	1	HIGH
stad_tcga_pan_can_atlas_2018	TCGA-BR-A4J4-01	TCGA-BR-A4J4	1	LOW
stad_tcga_pan_can_atlas_2018	TCGA-BR-A4QL-01	TCGA-BR-A4QL	1	HIGH
stad_tcga_pan_can_atlas_2018	TCGA-CD-8527-01	TCGA-CD-8527	1	HIGH
stad_tcga_pan_can_atlas_2018	TCGA-CD-8528-01	TCGA-CD-8528	1	HIGH
stad_tcga_pan_can_atlas_2018	TCGA-CD-A487-01	TCGA-CD-A487	1	HIGH
stad_tcga_pan_can_atlas_2018	TCGA-CG-4305-01	TCGA-CG-4305	1	HIGH
stad_tcga_pan_can_atlas_2018	TCGA-CG-4440-01	TCGA-CG-4440	1	HIGH
stad_tcga_pan_can_atlas_2018	TCGA-CG-4442-01	TCGA-CG-4442	1	HIGH
stad_tcga_pan_can_atlas_2018	TCGA-CG-4465-01	TCGA-CG-4465	1	S210N
stad_tcga_pan_can_atlas_2018	TCGA-CG-5734-01	TCGA-CG-5734	1	LOW
stad_tcga_pan_can_atlas_2018	TCGA-D7-6818-01	TCGA-D7-6818	1	LOW
stad_tcga_pan_can_atlas_2018	TCGA-D7-A4YX-01	TCGA-D7-A4YX	1	LOW
stad_tcga_pan_can_atlas_2018	TCGA-D7-A6EV-01	TCGA-D7-A6EV	1	HIGH
stad_tcga_pan_can_atlas_2018	TCGA-HU-A4G9-01	TCGA-HU-A4G9	1	HIGH
stad_tcga_pan_can_atlas_2018	TCGA-HU-A4GD-01	TCGA-HU-A4GD	1	HIGH
stad_tcga_pan_can_atlas_2018	TCGA-HU-A4GF-01	TCGA-HU-A4GF	1	HIGH
stad_tcga_pan_can_atlas_2018	TCGA-HU-A4H2-01	TCGA-HU-A4H2	1	HIGH
stad_tcga_pan_can_atlas_2018	TCGA-HU-A4HD-01	TCGA-HU-A4HD	1	HIGH
stad_tcga_pan_can_atlas_2018	TCGA-KB-A93J-01	TCGA-KB-A93J	1	LOW
stad_tcga_pan_can_atlas_2018	TCGA-MX-A5UJ-01	TCGA-MX-A5UJ	1	P16Rfs*7
stad_tcga_pan_can_atlas_2018	TCGA-RD-A8MW-01	TCGA-RD-A8MW	1	HIGH
stad_tcga_pan_can_atlas_2018	TCGA-RD-A8N1-01	TCGA-RD-A8N1	1	HIGH
stad_tcga_pan_can_atlas_2018	TCGA-RD-A8N2-01	TCGA-RD-A8N2	1	HIGH
stad_tcga_pan_can_atlas_2018	TCGA-VQ-A8DT-01	TCGA-VQ-A8DT	1	HIGH
stad_tcga_pan_can_atlas_2018	TCGA-VQ-A8E3-01	TCGA-VQ-A8E3	1	HIGH
stad_tcga_pan_can_atlas_2018	TCGA-VQ-A8P2-01	TCGA-VQ-A8P2	1	HIGH, E413K
stad_tcga_pan_can_atlas_2018	TCGA-VQ-A8P5-01	TCGA-VQ-A8P5	1	HIGH
stad_tcga_pan_can_atlas_2018	TCGA-VQ-A8PJ-01	TCGA-VQ-A8PJ	1	HIGH
stad_tcga_pan_can_atlas_2018	TCGA-VQ-A8PK-01	TCGA-VQ-A8PK	1	HIGH
stad_tcga_pan_can_atlas_2018	TCGA-VQ-A91Z-01	TCGA-VQ-A91Z	1	HIGH
stad_tcga_pan_can_atlas_2018	TCGA-VQ-A922-01	TCGA-VQ-A922	1	AMP

stad_tcga_pan_can_atlas_2018	TCGA-VQ-A94P-01	TCGA-VQ-A94P	1 HIGH
stad_tcga_pan_can_atlas_2018	TCGA-VQ-A94T-01	TCGA-VQ-A94T	1 HIGH
stad_tcga_pan_can_atlas_2018	TCGA-VQ-AA68-01	TCGA-VQ-AA68	1 HIGH
stad_tcga_pan_can_atlas_2018	TCGA-3M-AB46-01	TCGA-3M-AB46	0 no alteration
stad_tcga_pan_can_atlas_2018	TCGA-3M-AB47-01	TCGA-3M-AB47	0 no alteration
stad_tcga_pan_can_atlas_2018	TCGA-B7-5816-01	TCGA-B7-5816	0 no alteration
stad_tcga_pan_can_atlas_2018	TCGA-B7-5818-01	TCGA-B7-5818	0 no alteration
stad_tcga_pan_can_atlas_2018	TCGA-B7-A5TI-01	TCGA-B7-A5TI	0 no alteration
stad_tcga_pan_can_atlas_2018	TCGA-B7-A5TJ-01	TCGA-B7-A5TJ	0 no alteration
stad_tcga_pan_can_atlas_2018	TCGA-B7-A5TK-01	TCGA-B7-A5TK	0 no alteration
stad_tcga_pan_can_atlas_2018	TCGA-B7-A5TN-01	TCGA-B7-A5TN	0 no alteration
stad_tcga_pan_can_atlas_2018	TCGA-BR-4187-01	TCGA-BR-4187	0 no alteration
stad_tcga_pan_can_atlas_2018	TCGA-BR-4191-01	TCGA-BR-4191	0 no alteration
stad_tcga_pan_can_atlas_2018	TCGA-BR-4201-01	TCGA-BR-4201	0 no alteration
stad_tcga_pan_can_atlas_2018	TCGA-BR-4253-01	TCGA-BR-4253	0 no alteration
stad_tcga_pan_can_atlas_2018	TCGA-BR-4255-01	TCGA-BR-4255	0 no alteration
stad_tcga_pan_can_atlas_2018	TCGA-BR-4256-01	TCGA-BR-4256	0 no alteration
stad_tcga_pan_can_atlas_2018	TCGA-BR-4257-01	TCGA-BR-4257	0 no alteration
stad_tcga_pan_can_atlas_2018	TCGA-BR-4267-01	TCGA-BR-4267	0 no alteration
stad_tcga_pan_can_atlas_2018	TCGA-BR-4279-01	TCGA-BR-4279	0 no alteration
stad_tcga_pan_can_atlas_2018	TCGA-BR-4280-01	TCGA-BR-4280	0 no alteration
stad_tcga_pan_can_atlas_2018	TCGA-BR-4292-01	TCGA-BR-4292	0 no alteration
stad_tcga_pan_can_atlas_2018	TCGA-BR-4294-01	TCGA-BR-4294	0 no alteration
stad_tcga_pan_can_atlas_2018	TCGA-BR-4357-01	TCGA-BR-4357	0 no alteration
stad_tcga_pan_can_atlas_2018	TCGA-BR-4361-01	TCGA-BR-4361	0 no alteration
stad_tcga_pan_can_atlas_2018	TCGA-BR-4362-01	TCGA-BR-4362	0 no alteration
stad_tcga_pan_can_atlas_2018	TCGA-BR-4363-01	TCGA-BR-4363	0 no alteration
stad_tcga_pan_can_atlas_2018	TCGA-BR-4366-01	TCGA-BR-4366	0 no alteration
stad_tcga_pan_can_atlas_2018	TCGA-BR-4369-01	TCGA-BR-4369	0 no alteration
stad_tcga_pan_can_atlas_2018	TCGA-BR-4370-01	TCGA-BR-4370	0 no alteration
stad_tcga_pan_can_atlas_2018	TCGA-BR-4371-01	TCGA-BR-4371	0 no alteration
stad_tcga_pan_can_atlas_2018	TCGA-BR-6453-01	TCGA-BR-6453	0 no alteration
stad_tcga_pan_can_atlas_2018	TCGA-BR-6454-01	TCGA-BR-6454	0 no alteration
stad_tcga_pan_can_atlas_2018	TCGA-BR-6455-01	TCGA-BR-6455	0 no alteration
stad_tcga_pan_can_atlas_2018	TCGA-BR-6456-01	TCGA-BR-6456	0 no alteration
stad_tcga_pan_can_atlas_2018	TCGA-BR-6457-01	TCGA-BR-6457	0 no alteration
stad_tcga_pan_can_atlas_2018	TCGA-BR-6458-01	TCGA-BR-6458	0 no alteration
stad_tcga_pan_can_atlas_2018	TCGA-BR-6563-01	TCGA-BR-6563	0 no alteration
stad_tcga_pan_can_atlas_2018	TCGA-BR-6564-01	TCGA-BR-6564	0 no alteration
stad_tcga_pan_can_atlas_2018	TCGA-BR-6565-01	TCGA-BR-6565	0 no alteration
stad_tcga_pan_can_atlas_2018	TCGA-BR-6566-01	TCGA-BR-6566	0 no alteration
stad_tcga_pan_can_atlas_2018	TCGA-BR-6705-01	TCGA-BR-6705	0 no alteration
stad_tcga_pan_can_atlas_2018	TCGA-BR-6706-01	TCGA-BR-6706	0 no alteration

stad_tcga_pan_can_atlas_2018	TCGA-BR-6709-01	TCGA-BR-6709	0 no alteration
stad_tcga_pan_can_atlas_2018	TCGA-BR-6801-01	TCGA-BR-6801	0 no alteration
stad_tcga_pan_can_atlas_2018	TCGA-BR-6802-01	TCGA-BR-6802	0 no alteration
stad_tcga_pan_can_atlas_2018	TCGA-BR-6803-01	TCGA-BR-6803	0 no alteration
stad_tcga_pan_can_atlas_2018	TCGA-BR-6852-01	TCGA-BR-6852	0 no alteration
stad_tcga_pan_can_atlas_2018	TCGA-BR-7196-01	TCGA-BR-7196	0 no alteration
stad_tcga_pan_can_atlas_2018	TCGA-BR-7197-01	TCGA-BR-7197	0 no alteration
stad_tcga_pan_can_atlas_2018	TCGA-BR-7703-01	TCGA-BR-7703	0 no alteration
stad_tcga_pan_can_atlas_2018	TCGA-BR-7704-01	TCGA-BR-7704	0 no alteration
stad_tcga_pan_can_atlas_2018	TCGA-BR-7707-01	TCGA-BR-7707	0 no alteration
stad_tcga_pan_can_atlas_2018	TCGA-BR-7715-01	TCGA-BR-7715	0 no alteration
stad_tcga_pan_can_atlas_2018	TCGA-BR-7716-01	TCGA-BR-7716	0 no alteration
stad_tcga_pan_can_atlas_2018	TCGA-BR-7717-01	TCGA-BR-7717	0 no alteration
stad_tcga_pan_can_atlas_2018	TCGA-BR-7722-01	TCGA-BR-7722	0 no alteration
stad_tcga_pan_can_atlas_2018	TCGA-BR-7723-01	TCGA-BR-7723	0 no alteration
stad_tcga_pan_can_atlas_2018	TCGA-BR-7851-01	TCGA-BR-7851	0 no alteration
stad_tcga_pan_can_atlas_2018	TCGA-BR-7901-01	TCGA-BR-7901	0 no alteration
stad_tcga_pan_can_atlas_2018	TCGA-BR-7957-01	TCGA-BR-7957	0 no alteration
stad_tcga_pan_can_atlas_2018	TCGA-BR-7958-01	TCGA-BR-7958	0 no alteration
stad_tcga_pan_can_atlas_2018	TCGA-BR-7959-01	TCGA-BR-7959	0 no alteration
stad_tcga_pan_can_atlas_2018	TCGA-BR-8058-01	TCGA-BR-8058	0 no alteration
stad_tcga_pan_can_atlas_2018	TCGA-BR-8059-01	TCGA-BR-8059	0 no alteration
stad_tcga_pan_can_atlas_2018	TCGA-BR-8060-01	TCGA-BR-8060	0 no alteration
stad_tcga_pan_can_atlas_2018	TCGA-BR-8077-01	TCGA-BR-8077	0 no alteration
stad_tcga_pan_can_atlas_2018	TCGA-BR-8080-01	TCGA-BR-8080	0 no alteration
stad_tcga_pan_can_atlas_2018	TCGA-BR-8081-01	TCGA-BR-8081	0 no alteration
stad_tcga_pan_can_atlas_2018	TCGA-BR-8284-01	TCGA-BR-8284	0 no alteration
stad_tcga_pan_can_atlas_2018	TCGA-BR-8286-01	TCGA-BR-8286	0 no alteration
stad_tcga_pan_can_atlas_2018	TCGA-BR-8289-01	TCGA-BR-8289	0 no alteration
stad_tcga_pan_can_atlas_2018	TCGA-BR-8291-01	TCGA-BR-8291	0 no alteration
stad_tcga_pan_can_atlas_2018	TCGA-BR-8297-01	TCGA-BR-8297	0 no alteration
stad_tcga_pan_can_atlas_2018	TCGA-BR-8364-01	TCGA-BR-8364	0 no alteration
stad_tcga_pan_can_atlas_2018	TCGA-BR-8365-01	TCGA-BR-8365	0 no alteration
stad_tcga_pan_can_atlas_2018	TCGA-BR-8366-01	TCGA-BR-8366	0 no alteration
stad_tcga_pan_can_atlas_2018	TCGA-BR-8367-01	TCGA-BR-8367	0 no alteration
stad_tcga_pan_can_atlas_2018	TCGA-BR-8368-01	TCGA-BR-8368	0 no alteration
stad_tcga_pan_can_atlas_2018	TCGA-BR-8371-01	TCGA-BR-8371	0 no alteration
stad_tcga_pan_can_atlas_2018	TCGA-BR-8372-01	TCGA-BR-8372	0 no alteration
stad_tcga_pan_can_atlas_2018	TCGA-BR-8373-01	TCGA-BR-8373	0 no alteration
stad_tcga_pan_can_atlas_2018	TCGA-BR-8380-01	TCGA-BR-8380	0 no alteration
stad_tcga_pan_can_atlas_2018	TCGA-BR-8381-01	TCGA-BR-8381	0 no alteration
stad_tcga_pan_can_atlas_2018	TCGA-BR-8382-01	TCGA-BR-8382	0 no alteration
stad_tcga_pan_can_atlas_2018	TCGA-BR-8384-01	TCGA-BR-8384	0 no alteration

stad_tcga_pan_can_atlas_2018	TCGA-BR-8483-01	TCGA-BR-8483	0 no alteration
stad_tcga_pan_can_atlas_2018	TCGA-BR-8484-01	TCGA-BR-8484	0 no alteration
stad_tcga_pan_can_atlas_2018	TCGA-BR-8485-01	TCGA-BR-8485	0 no alteration
stad_tcga_pan_can_atlas_2018	TCGA-BR-8486-01	TCGA-BR-8486	0 no alteration
stad_tcga_pan_can_atlas_2018	TCGA-BR-8487-01	TCGA-BR-8487	0 no alteration
stad_tcga_pan_can_atlas_2018	TCGA-BR-8588-01	TCGA-BR-8588	0 no alteration
stad_tcga_pan_can_atlas_2018	TCGA-BR-8589-01	TCGA-BR-8589	0 no alteration
stad_tcga_pan_can_atlas_2018	TCGA-BR-8590-01	TCGA-BR-8590	0 no alteration
stad_tcga_pan_can_atlas_2018	TCGA-BR-8591-01	TCGA-BR-8591	0 no alteration
stad_tcga_pan_can_atlas_2018	TCGA-BR-8592-01	TCGA-BR-8592	0 no alteration
stad_tcga_pan_can_atlas_2018	TCGA-BR-8676-01	TCGA-BR-8676	0 no alteration
stad_tcga_pan_can_atlas_2018	TCGA-BR-8677-01	TCGA-BR-8677	0 no alteration
stad_tcga_pan_can_atlas_2018	TCGA-BR-8678-01	TCGA-BR-8678	0 no alteration
stad_tcga_pan_can_atlas_2018	TCGA-BR-8679-01	TCGA-BR-8679	0 no alteration
stad_tcga_pan_can_atlas_2018	TCGA-BR-8680-01	TCGA-BR-8680	0 no alteration
stad_tcga_pan_can_atlas_2018	TCGA-BR-8682-01	TCGA-BR-8682	0 no alteration
stad_tcga_pan_can_atlas_2018	TCGA-BR-8683-01	TCGA-BR-8683	0 no alteration
stad_tcga_pan_can_atlas_2018	TCGA-BR-8686-01	TCGA-BR-8686	0 no alteration
stad_tcga_pan_can_atlas_2018	TCGA-BR-8690-01	TCGA-BR-8690	0 no alteration
stad_tcga_pan_can_atlas_2018	TCGA-BR-A44T-01	TCGA-BR-A44T	0 no alteration
stad_tcga_pan_can_atlas_2018	TCGA-BR-A44U-01	TCGA-BR-A44U	0 no alteration
stad_tcga_pan_can_atlas_2018	TCGA-BR-A452-01	TCGA-BR-A452	0 no alteration
stad_tcga_pan_can_atlas_2018	TCGA-BR-A453-01	TCGA-BR-A453	0 no alteration
stad_tcga_pan_can_atlas_2018	TCGA-BR-A4CR-01	TCGA-BR-A4CR	0 no alteration
stad_tcga_pan_can_atlas_2018	TCGA-BR-A4CS-01	TCGA-BR-A4CS	0 no alteration
stad_tcga_pan_can_atlas_2018	TCGA-BR-A4IU-01	TCGA-BR-A4IU	0 no alteration
stad_tcga_pan_can_atlas_2018	TCGA-BR-A4IY-01	TCGA-BR-A4IY	0 no alteration
stad_tcga_pan_can_atlas_2018	TCGA-BR-A4IZ-01	TCGA-BR-A4IZ	0 no alteration
stad_tcga_pan_can_atlas_2018	TCGA-BR-A4J1-01	TCGA-BR-A4J1	0 no alteration
stad_tcga_pan_can_atlas_2018	TCGA-BR-A4J2-01	TCGA-BR-A4J2	0 no alteration
stad_tcga_pan_can_atlas_2018	TCGA-BR-A4J5-01	TCGA-BR-A4J5	0 no alteration
stad_tcga_pan_can_atlas_2018	TCGA-BR-A4J6-01	TCGA-BR-A4J6	0 no alteration
stad_tcga_pan_can_atlas_2018	TCGA-BR-A4J7-01	TCGA-BR-A4J7	0 no alteration
stad_tcga_pan_can_atlas_2018	TCGA-BR-A4J8-01	TCGA-BR-A4J8	0 no alteration
stad_tcga_pan_can_atlas_2018	TCGA-BR-A4J9-01	TCGA-BR-A4J9	0 no alteration
stad_tcga_pan_can_atlas_2018	TCGA-BR-A4PD-01	TCGA-BR-A4PD	0 no alteration
stad_tcga_pan_can_atlas_2018	TCGA-BR-A4PE-01	TCGA-BR-A4PE	0 no alteration
stad_tcga_pan_can_atlas_2018	TCGA-BR-A4PF-01	TCGA-BR-A4PF	0 no alteration
stad_tcga_pan_can_atlas_2018	TCGA-BR-A4QI-01	TCGA-BR-A4QI	0 no alteration
stad_tcga_pan_can_atlas_2018	TCGA-BR-A4QM-01	TCGA-BR-A4QM	0 no alteration
stad_tcga_pan_can_atlas_2018	TCGA-CD-5798-01	TCGA-CD-5798	0 no alteration
stad_tcga_pan_can_atlas_2018	TCGA-CD-5799-01	TCGA-CD-5799	0 no alteration
stad_tcga_pan_can_atlas_2018	TCGA-CD-5800-01	TCGA-CD-5800	0 no alteration

stad_tcga_pan_can_atlas_2018	TCGA-CD-5801-01	TCGA-CD-5801	0 no alteration
stad_tcga_pan_can_atlas_2018	TCGA-CD-5803-01	TCGA-CD-5803	0 no alteration
stad_tcga_pan_can_atlas_2018	TCGA-CD-5804-01	TCGA-CD-5804	0 no alteration
stad_tcga_pan_can_atlas_2018	TCGA-CD-8524-01	TCGA-CD-8524	0 no alteration
stad_tcga_pan_can_atlas_2018	TCGA-CD-8525-01	TCGA-CD-8525	0 no alteration
stad_tcga_pan_can_atlas_2018	TCGA-CD-8526-01	TCGA-CD-8526	0 no alteration
stad_tcga_pan_can_atlas_2018	TCGA-CD-8529-01	TCGA-CD-8529	0 no alteration
stad_tcga_pan_can_atlas_2018	TCGA-CD-8530-01	TCGA-CD-8530	0 no alteration
stad_tcga_pan_can_atlas_2018	TCGA-CD-8531-01	TCGA-CD-8531	0 no alteration
stad_tcga_pan_can_atlas_2018	TCGA-CD-8532-01	TCGA-CD-8532	0 no alteration
stad_tcga_pan_can_atlas_2018	TCGA-CD-8533-01	TCGA-CD-8533	0 no alteration
stad_tcga_pan_can_atlas_2018	TCGA-CD-8534-01	TCGA-CD-8534	0 no alteration
stad_tcga_pan_can_atlas_2018	TCGA-CD-8535-01	TCGA-CD-8535	0 no alteration
stad_tcga_pan_can_atlas_2018	TCGA-CD-8536-01	TCGA-CD-8536	0 no alteration
stad_tcga_pan_can_atlas_2018	TCGA-CD-A486-01	TCGA-CD-A486	0 no alteration
stad_tcga_pan_can_atlas_2018	TCGA-CD-A489-01	TCGA-CD-A489	0 no alteration
stad_tcga_pan_can_atlas_2018	TCGA-CD-A48A-01	TCGA-CD-A48A	0 no alteration
stad_tcga_pan_can_atlas_2018	TCGA-CD-A48C-01	TCGA-CD-A48C	0 no alteration
stad_tcga_pan_can_atlas_2018	TCGA-CD-A4MG-01	TCGA-CD-A4MG	0 no alteration
stad_tcga_pan_can_atlas_2018	TCGA-CD-A4MH-01	TCGA-CD-A4MH	0 no alteration
stad_tcga_pan_can_atlas_2018	TCGA-CD-A4MI-01	TCGA-CD-A4MI	0 no alteration
stad_tcga_pan_can_atlas_2018	TCGA-CD-A4MJ-01	TCGA-CD-A4MJ	0 no alteration
stad_tcga_pan_can_atlas_2018	TCGA-CG-4301-01	TCGA-CG-4301	0 no alteration
stad_tcga_pan_can_atlas_2018	TCGA-CG-4304-01	TCGA-CG-4304	0 no alteration
stad_tcga_pan_can_atlas_2018	TCGA-CG-4306-01	TCGA-CG-4306	0 no alteration
stad_tcga_pan_can_atlas_2018	TCGA-CG-4436-01	TCGA-CG-4436	0 no alteration
stad_tcga_pan_can_atlas_2018	TCGA-CG-4437-01	TCGA-CG-4437	0 no alteration
stad_tcga_pan_can_atlas_2018	TCGA-CG-4438-01	TCGA-CG-4438	0 no alteration
stad_tcga_pan_can_atlas_2018	TCGA-CG-4441-01	TCGA-CG-4441	0 no alteration
stad_tcga_pan_can_atlas_2018	TCGA-CG-4443-01	TCGA-CG-4443	0 no alteration
stad_tcga_pan_can_atlas_2018	TCGA-CG-4444-01	TCGA-CG-4444	0 no alteration
stad_tcga_pan_can_atlas_2018	TCGA-CG-4449-01	TCGA-CG-4449	0 no alteration
stad_tcga_pan_can_atlas_2018	TCGA-CG-4462-01	TCGA-CG-4462	0 no alteration
stad_tcga_pan_can_atlas_2018	TCGA-CG-4466-01	TCGA-CG-4466	0 no alteration
stad_tcga_pan_can_atlas_2018	TCGA-CG-4469-01	TCGA-CG-4469	0 no alteration
stad_tcga_pan_can_atlas_2018	TCGA-CG-4474-01	TCGA-CG-4474	0 no alteration
stad_tcga_pan_can_atlas_2018	TCGA-CG-4475-01	TCGA-CG-4475	0 no alteration
stad_tcga_pan_can_atlas_2018	TCGA-CG-4476-01	TCGA-CG-4476	0 no alteration
stad_tcga_pan_can_atlas_2018	TCGA-CG-4477-01	TCGA-CG-4477	0 no alteration
stad_tcga_pan_can_atlas_2018	TCGA-CG-5717-01	TCGA-CG-5717	0 no alteration
stad_tcga_pan_can_atlas_2018	TCGA-CG-5718-01	TCGA-CG-5718	0 no alteration
stad_tcga_pan_can_atlas_2018	TCGA-CG-5719-01	TCGA-CG-5719	0 no alteration
stad_tcga_pan_can_atlas_2018	TCGA-CG-5720-01	TCGA-CG-5720	0 no alteration

stad_tcga_pan_can_atlas_2018	TCGA-CG-5721-01	TCGA-CG-5721	0	no alteration
stad_tcga_pan_can_atlas_2018	TCGA-CG-5722-01	TCGA-CG-5722	0	no alteration
stad_tcga_pan_can_atlas_2018	TCGA-CG-5723-01	TCGA-CG-5723	0	no alteration
stad_tcga_pan_can_atlas_2018	TCGA-CG-5724-01	TCGA-CG-5724	0	no alteration
stad_tcga_pan_can_atlas_2018	TCGA-CG-5725-01	TCGA-CG-5725	0	no alteration
stad_tcga_pan_can_atlas_2018	TCGA-CG-5726-01	TCGA-CG-5726	0	no alteration
stad_tcga_pan_can_atlas_2018	TCGA-CG-5732-01	TCGA-CG-5732	0	no alteration
stad_tcga_pan_can_atlas_2018	TCGA-D7-5577-01	TCGA-D7-5577	0	no alteration
stad_tcga_pan_can_atlas_2018	TCGA-D7-5578-01	TCGA-D7-5578	0	no alteration
stad_tcga_pan_can_atlas_2018	TCGA-D7-6518-01	TCGA-D7-6518	0	no alteration
stad_tcga_pan_can_atlas_2018	TCGA-D7-6519-01	TCGA-D7-6519	0	no alteration
stad_tcga_pan_can_atlas_2018	TCGA-D7-6520-01	TCGA-D7-6520	0	no alteration
stad_tcga_pan_can_atlas_2018	TCGA-D7-6521-01	TCGA-D7-6521	0	no alteration
stad_tcga_pan_can_atlas_2018	TCGA-D7-6522-01	TCGA-D7-6522	0	no alteration
stad_tcga_pan_can_atlas_2018	TCGA-D7-6524-01	TCGA-D7-6524	0	no alteration
stad_tcga_pan_can_atlas_2018	TCGA-D7-6525-01	TCGA-D7-6525	0	no alteration
stad_tcga_pan_can_atlas_2018	TCGA-D7-6526-01	TCGA-D7-6526	0	no alteration
stad_tcga_pan_can_atlas_2018	TCGA-D7-6527-01	TCGA-D7-6527	0	no alteration
stad_tcga_pan_can_atlas_2018	TCGA-D7-6528-01	TCGA-D7-6528	0	no alteration
stad_tcga_pan_can_atlas_2018	TCGA-D7-6815-01	TCGA-D7-6815	0	no alteration
stad_tcga_pan_can_atlas_2018	TCGA-D7-6817-01	TCGA-D7-6817	0	no alteration
stad_tcga_pan_can_atlas_2018	TCGA-D7-6820-01	TCGA-D7-6820	0	no alteration
stad_tcga_pan_can_atlas_2018	TCGA-D7-6822-01	TCGA-D7-6822	0	no alteration
stad_tcga_pan_can_atlas_2018	TCGA-D7-8570-01	TCGA-D7-8570	0	no alteration
stad_tcga_pan_can_atlas_2018	TCGA-D7-8572-01	TCGA-D7-8572	0	no alteration
stad_tcga_pan_can_atlas_2018	TCGA-D7-8573-01	TCGA-D7-8573	0	no alteration
stad_tcga_pan_can_atlas_2018	TCGA-D7-8574-01	TCGA-D7-8574	0	no alteration
stad_tcga_pan_can_atlas_2018	TCGA-D7-8575-01	TCGA-D7-8575	0	no alteration
stad_tcga_pan_can_atlas_2018	TCGA-D7-8576-01	TCGA-D7-8576	0	no alteration
stad_tcga_pan_can_atlas_2018	TCGA-D7-8578-01	TCGA-D7-8578	0	no alteration
stad_tcga_pan_can_atlas_2018	TCGA-D7-8579-01	TCGA-D7-8579	0	no alteration
stad_tcga_pan_can_atlas_2018	TCGA-D7-A4YT-01	TCGA-D7-A4YT	0	no alteration
stad_tcga_pan_can_atlas_2018	TCGA-D7-A4YU-01	TCGA-D7-A4YU	0	no alteration
stad_tcga_pan_can_atlas_2018	TCGA-D7-A4YV-01	TCGA-D7-A4YV	0	no alteration
stad_tcga_pan_can_atlas_2018	TCGA-D7-A4YY-01	TCGA-D7-A4YY	0	no alteration
stad_tcga_pan_can_atlas_2018	TCGA-D7-A4Z0-01	TCGA-D7-A4Z0	0	no alteration
stad_tcga_pan_can_atlas_2018	TCGA-D7-A6EX-01	TCGA-D7-A6EX	0	no alteration
stad_tcga_pan_can_atlas_2018	TCGA-D7-A6EY-01	TCGA-D7-A6EY	0	no alteration
stad_tcga_pan_can_atlas_2018	TCGA-D7-A6EZ-01	TCGA-D7-A6EZ	0	no alteration
stad_tcga_pan_can_atlas_2018	TCGA-D7-A6F0-01	TCGA-D7-A6F0	0	no alteration
stad_tcga_pan_can_atlas_2018	TCGA-D7-A6F2-01	TCGA-D7-A6F2	0	no alteration
stad_tcga_pan_can_atlas_2018	TCGA-D7-A747-01	TCGA-D7-A747	0	no alteration
stad_tcga_pan_can_atlas_2018	TCGA-D7-A748-01	TCGA-D7-A748	0	no alteration

stad_tcga_pan_can_atlas_2018	TCGA-D7-A74A-01	TCGA-D7-A74A	0 no alteration
stad_tcga_pan_can_atlas_2018	TCGA-EQ-8122-01	TCGA-EQ-8122	0 no alteration
stad_tcga_pan_can_atlas_2018	TCGA-EQ-A4SO-01	TCGA-EQ-A4SO	0 no alteration
stad_tcga_pan_can_atlas_2018	TCGA-F1-6177-01	TCGA-F1-6177	0 no alteration
stad_tcga_pan_can_atlas_2018	TCGA-F1-6874-01	TCGA-F1-6874	0 no alteration
stad_tcga_pan_can_atlas_2018	TCGA-F1-6875-01	TCGA-F1-6875	0 no alteration
stad_tcga_pan_can_atlas_2018	TCGA-F1-A448-01	TCGA-F1-A448	0 no alteration
stad_tcga_pan_can_atlas_2018	TCGA-F1-A72C-01	TCGA-F1-A72C	0 no alteration
stad_tcga_pan_can_atlas_2018	TCGA-FP-7735-01	TCGA-FP-7735	0 no alteration
stad_tcga_pan_can_atlas_2018	TCGA-FP-7829-01	TCGA-FP-7829	0 no alteration
stad_tcga_pan_can_atlas_2018	TCGA-FP-7916-01	TCGA-FP-7916	0 no alteration
stad_tcga_pan_can_atlas_2018	TCGA-FP-7998-01	TCGA-FP-7998	0 no alteration
stad_tcga_pan_can_atlas_2018	TCGA-FP-8099-01	TCGA-FP-8099	0 no alteration
stad_tcga_pan_can_atlas_2018	TCGA-FP-8209-01	TCGA-FP-8209	0 no alteration
stad_tcga_pan_can_atlas_2018	TCGA-FP-8210-01	TCGA-FP-8210	0 no alteration
stad_tcga_pan_can_atlas_2018	TCGA-FP-8211-01	TCGA-FP-8211	0 no alteration
stad_tcga_pan_can_atlas_2018	TCGA-FP-8631-01	TCGA-FP-8631	0 no alteration
stad_tcga_pan_can_atlas_2018	TCGA-FP-A4BF-01	TCGA-FP-A4BF	0 no alteration
stad_tcga_pan_can_atlas_2018	TCGA-FP-A8CX-01	TCGA-FP-A8CX	0 no alteration
stad_tcga_pan_can_atlas_2018	TCGA-FP-A9TM-01	TCGA-FP-A9TM	0 no alteration
stad_tcga_pan_can_atlas_2018	TCGA-HF-7132-01	TCGA-HF-7132	0 no alteration
stad_tcga_pan_can_atlas_2018	TCGA-HF-7133-01	TCGA-HF-7133	0 no alteration
stad_tcga_pan_can_atlas_2018	TCGA-HF-7134-01	TCGA-HF-7134	0 no alteration
stad_tcga_pan_can_atlas_2018	TCGA-HF-7136-01	TCGA-HF-7136	0 no alteration
stad_tcga_pan_can_atlas_2018	TCGA-HF-A5NB-01	TCGA-HF-A5NB	0 no alteration
stad_tcga_pan_can_atlas_2018	TCGA-HJ-7597-01	TCGA-HJ-7597	0 no alteration
stad_tcga_pan_can_atlas_2018	TCGA-HU-8238-01	TCGA-HU-8238	0 no alteration
stad_tcga_pan_can_atlas_2018	TCGA-HU-8243-01	TCGA-HU-8243	0 no alteration
stad_tcga_pan_can_atlas_2018	TCGA-HU-8244-01	TCGA-HU-8244	0 no alteration
stad_tcga_pan_can_atlas_2018	TCGA-HU-8249-01	TCGA-HU-8249	0 no alteration
stad_tcga_pan_can_atlas_2018	TCGA-HU-8602-01	TCGA-HU-8602	0 no alteration
stad_tcga_pan_can_atlas_2018	TCGA-HU-8604-01	TCGA-HU-8604	0 no alteration
stad_tcga_pan_can_atlas_2018	TCGA-HU-8608-01	TCGA-HU-8608	0 no alteration
stad_tcga_pan_can_atlas_2018	TCGA-HU-8610-01	TCGA-HU-8610	0 no alteration
stad_tcga_pan_can_atlas_2018	TCGA-HU-A4G2-01	TCGA-HU-A4G2	0 no alteration
stad_tcga_pan_can_atlas_2018	TCGA-HU-A4G3-01	TCGA-HU-A4G3	0 no alteration
stad_tcga_pan_can_atlas_2018	TCGA-HU-A4G6-01	TCGA-HU-A4G6	0 no alteration
stad_tcga_pan_can_atlas_2018	TCGA-HU-A4G8-01	TCGA-HU-A4G8	0 no alteration
stad_tcga_pan_can_atlas_2018	TCGA-HU-A4GC-01	TCGA-HU-A4GC	0 no alteration
stad_tcga_pan_can_atlas_2018	TCGA-HU-A4GH-01	TCGA-HU-A4GH	0 no alteration
stad_tcga_pan_can_atlas_2018	TCGA-HU-A4GJ-01	TCGA-HU-A4GJ	0 no alteration
stad_tcga_pan_can_atlas_2018	TCGA-HU-A4GN-01	TCGA-HU-A4GN	0 no alteration
stad_tcga_pan_can_atlas_2018	TCGA-HU-A4GP-01	TCGA-HU-A4GP	0 no alteration

stad_tcga_pan_can_atlas_2018	TCGA-HU-A4GQ-01	TCGA-HU-A4GQ	0	no alteration
stad_tcga_pan_can_atlas_2018	TCGA-HU-A4GT-01	TCGA-HU-A4GT	0	no alteration
stad_tcga_pan_can_atlas_2018	TCGA-HU-A4GU-01	TCGA-HU-A4GU	0	no alteration
stad_tcga_pan_can_atlas_2018	TCGA-HU-A4GX-01	TCGA-HU-A4GX	0	no alteration
stad_tcga_pan_can_atlas_2018	TCGA-HU-A4GY-01	TCGA-HU-A4GY	0	no alteration
stad_tcga_pan_can_atlas_2018	TCGA-HU-A4H0-01	TCGA-HU-A4H0	0	no alteration
stad_tcga_pan_can_atlas_2018	TCGA-HU-A4H3-01	TCGA-HU-A4H3	0	no alteration
stad_tcga_pan_can_atlas_2018	TCGA-HU-A4H4-01	TCGA-HU-A4H4	0	no alteration
stad_tcga_pan_can_atlas_2018	TCGA-HU-A4H5-01	TCGA-HU-A4H5	0	no alteration
stad_tcga_pan_can_atlas_2018	TCGA-HU-A4H6-01	TCGA-HU-A4H6	0	no alteration
stad_tcga_pan_can_atlas_2018	TCGA-HU-A4H8-01	TCGA-HU-A4H8	0	no alteration
stad_tcga_pan_can_atlas_2018	TCGA-HU-A4HB-01	TCGA-HU-A4HB	0	no alteration
stad_tcga_pan_can_atlas_2018	TCGA-IN-7806-01	TCGA-IN-7806	0	no alteration
stad_tcga_pan_can_atlas_2018	TCGA-IN-7808-01	TCGA-IN-7808	0	no alteration
stad_tcga_pan_can_atlas_2018	TCGA-IN-8462-01	TCGA-IN-8462	0	no alteration
stad_tcga_pan_can_atlas_2018	TCGA-IN-8663-01	TCGA-IN-8663	0	no alteration
stad_tcga_pan_can_atlas_2018	TCGA-IN-A6RI-01	TCGA-IN-A6RI	0	no alteration
stad_tcga_pan_can_atlas_2018	TCGA-IN-A6RJ-01	TCGA-IN-A6RJ	0	no alteration
stad_tcga_pan_can_atlas_2018	TCGA-IN-A6RL-01	TCGA-IN-A6RL	0	no alteration
stad_tcga_pan_can_atlas_2018	TCGA-IN-A6RN-01	TCGA-IN-A6RN	0	no alteration
stad_tcga_pan_can_atlas_2018	TCGA-IN-A6RO-01	TCGA-IN-A6RO	0	no alteration
stad_tcga_pan_can_atlas_2018	TCGA-IN-A6RR-01	TCGA-IN-A6RR	0	no alteration
stad_tcga_pan_can_atlas_2018	TCGA-IN-A6RS-01	TCGA-IN-A6RS	0	no alteration
stad_tcga_pan_can_atlas_2018	TCGA-IN-A7NR-01	TCGA-IN-A7NR	0	no alteration
stad_tcga_pan_can_atlas_2018	TCGA-IN-A7NT-01	TCGA-IN-A7NT	0	no alteration
stad_tcga_pan_can_atlas_2018	TCGA-IN-A7NU-01	TCGA-IN-A7NU	0	no alteration
stad_tcga_pan_can_atlas_2018	TCGA-IN-AB1V-01	TCGA-IN-AB1V	0	no alteration
stad_tcga_pan_can_atlas_2018	TCGA-IN-AB1X-01	TCGA-IN-AB1X	0	no alteration
stad_tcga_pan_can_atlas_2018	TCGA-IP-7968-01	TCGA-IP-7968	0	no alteration
stad_tcga_pan_can_atlas_2018	TCGA-KB-A6F7-01	TCGA-KB-A6F7	0	no alteration
stad_tcga_pan_can_atlas_2018	TCGA-KB-A93G-01	TCGA-KB-A93G	0	no alteration
stad_tcga_pan_can_atlas_2018	TCGA-KB-A93H-01	TCGA-KB-A93H	0	no alteration
stad_tcga_pan_can_atlas_2018	TCGA-MX-A5UG-01	TCGA-MX-A5UG	0	no alteration
stad_tcga_pan_can_atlas_2018	TCGA-MX-A663-01	TCGA-MX-A663	0	no alteration
stad_tcga_pan_can_atlas_2018	TCGA-MX-A666-01	TCGA-MX-A666	0	no alteration
stad_tcga_pan_can_atlas_2018	TCGA-R5-A7O7-01	TCGA-R5-A7O7	0	no alteration
stad_tcga_pan_can_atlas_2018	TCGA-R5-A7ZE-01	TCGA-R5-A7ZE	0	no alteration
stad_tcga_pan_can_atlas_2018	TCGA-R5-A7ZF-01	TCGA-R5-A7ZF	0	no alteration
stad_tcga_pan_can_atlas_2018	TCGA-R5-A7ZI-01	TCGA-R5-A7ZI	0	no alteration
stad_tcga_pan_can_atlas_2018	TCGA-R5-A7ZR-01	TCGA-R5-A7ZR	0	no alteration
stad_tcga_pan_can_atlas_2018	TCGA-R5-A805-01	TCGA-R5-A805	0	no alteration
stad_tcga_pan_can_atlas_2018	TCGA-RD-A7BS-01	TCGA-RD-A7BS	0	no alteration
stad_tcga_pan_can_atlas_2018	TCGA-RD-A7BT-01	TCGA-RD-A7BT	0	no alteration

stad_tcga_pan_can_atlas_2018	TCGA-RD-A7BW-01	TCGA-RD-A7BW	0	no alteration
stad_tcga_pan_can_atlas_2018	TCGA-RD-A7C1-01	TCGA-RD-A7C1	0	no alteration
stad_tcga_pan_can_atlas_2018	TCGA-RD-A8MV-01	TCGA-RD-A8MV	0	no alteration
stad_tcga_pan_can_atlas_2018	TCGA-RD-A8N0-01	TCGA-RD-A8N0	0	no alteration
stad_tcga_pan_can_atlas_2018	TCGA-RD-A8N4-01	TCGA-RD-A8N4	0	no alteration
stad_tcga_pan_can_atlas_2018	TCGA-RD-A8N5-01	TCGA-RD-A8N5	0	no alteration
stad_tcga_pan_can_atlas_2018	TCGA-RD-A8N6-01	TCGA-RD-A8N6	0	no alteration
stad_tcga_pan_can_atlas_2018	TCGA-RD-A8N9-01	TCGA-RD-A8N9	0	no alteration
stad_tcga_pan_can_atlas_2018	TCGA-RD-A8NB-01	TCGA-RD-A8NB	0	no alteration
stad_tcga_pan_can_atlas_2018	TCGA-SW-A7EA-01	TCGA-SW-A7EA	0	no alteration
stad_tcga_pan_can_atlas_2018	TCGA-SW-A7EB-01	TCGA-SW-A7EB	0	no alteration
stad_tcga_pan_can_atlas_2018	TCGA-VQ-A8DU-01	TCGA-VQ-A8DU	0	no alteration
stad_tcga_pan_can_atlas_2018	TCGA-VQ-A8DV-01	TCGA-VQ-A8DV	0	no alteration
stad_tcga_pan_can_atlas_2018	TCGA-VQ-A8DZ-01	TCGA-VQ-A8DZ	0	no alteration
stad_tcga_pan_can_atlas_2018	TCGA-VQ-A8E0-01	TCGA-VQ-A8E0	0	no alteration
stad_tcga_pan_can_atlas_2018	TCGA-VQ-A8E2-01	TCGA-VQ-A8E2	0	no alteration
stad_tcga_pan_can_atlas_2018	TCGA-VQ-A8E7-01	TCGA-VQ-A8E7	0	no alteration
stad_tcga_pan_can_atlas_2018	TCGA-VQ-A8P3-01	TCGA-VQ-A8P3	0	no alteration
stad_tcga_pan_can_atlas_2018	TCGA-VQ-A8P8-01	TCGA-VQ-A8P8	0	no alteration
stad_tcga_pan_can_atlas_2018	TCGA-VQ-A8PB-01	TCGA-VQ-A8PB	0	no alteration
stad_tcga_pan_can_atlas_2018	TCGA-VQ-A8PC-01	TCGA-VQ-A8PC	0	no alteration
stad_tcga_pan_can_atlas_2018	TCGA-VQ-A8PD-01	TCGA-VQ-A8PD	0	no alteration
stad_tcga_pan_can_atlas_2018	TCGA-VQ-A8PE-01	TCGA-VQ-A8PE	0	no alteration
stad_tcga_pan_can_atlas_2018	TCGA-VQ-A8PF-01	TCGA-VQ-A8PF	0	no alteration
stad_tcga_pan_can_atlas_2018	TCGA-VQ-A8PH-01	TCGA-VQ-A8PH	0	no alteration
stad_tcga_pan_can_atlas_2018	TCGA-VQ-A8PM-01	TCGA-VQ-A8PM	0	no alteration
stad_tcga_pan_can_atlas_2018	TCGA-VQ-A8PO-01	TCGA-VQ-A8PO	0	no alteration
stad_tcga_pan_can_atlas_2018	TCGA-VQ-A8PP-01	TCGA-VQ-A8PP	0	no alteration
stad_tcga_pan_can_atlas_2018	TCGA-VQ-A8PQ-01	TCGA-VQ-A8PQ	0	no alteration
stad_tcga_pan_can_atlas_2018	TCGA-VQ-A8PU-01	TCGA-VQ-A8PU	0	no alteration
stad_tcga_pan_can_atlas_2018	TCGA-VQ-A8PX-01	TCGA-VQ-A8PX	0	no alteration
stad_tcga_pan_can_atlas_2018	TCGA-VQ-A91A-01	TCGA-VQ-A91A	0	no alteration
stad_tcga_pan_can_atlas_2018	TCGA-VQ-A91D-01	TCGA-VQ-A91D	0	no alteration
stad_tcga_pan_can_atlas_2018	TCGA-VQ-A91E-01	TCGA-VQ-A91E	0	no alteration
stad_tcga_pan_can_atlas_2018	TCGA-VQ-A91K-01	TCGA-VQ-A91K	0	no alteration
stad_tcga_pan_can_atlas_2018	TCGA-VQ-A91N-01	TCGA-VQ-A91N	0	no alteration
stad_tcga_pan_can_atlas_2018	TCGA-VQ-A91Q-01	TCGA-VQ-A91Q	0	no alteration
stad_tcga_pan_can_atlas_2018	TCGA-VQ-A91S-01	TCGA-VQ-A91S	0	no alteration
stad_tcga_pan_can_atlas_2018	TCGA-VQ-A91U-01	TCGA-VQ-A91U	0	no alteration
stad_tcga_pan_can_atlas_2018	TCGA-VQ-A91V-01	TCGA-VQ-A91V	0	no alteration
stad_tcga_pan_can_atlas_2018	TCGA-VQ-A91W-01	TCGA-VQ-A91W	0	no alteration
stad_tcga_pan_can_atlas_2018	TCGA-VQ-A91X-01	TCGA-VQ-A91X	0	no alteration
stad_tcga_pan_can_atlas_2018	TCGA-VQ-A91Y-01	TCGA-VQ-A91Y	0	no alteration

stad_tcga_pan_can_atlas_2018	TCGA-VQ-A923-01	TCGA-VQ-A923	0	no alteration
stad_tcga_pan_can_atlas_2018	TCGA-VQ-A924-01	TCGA-VQ-A924	0	no alteration
stad_tcga_pan_can_atlas_2018	TCGA-VQ-A925-01	TCGA-VQ-A925	0	no alteration
stad_tcga_pan_can_atlas_2018	TCGA-VQ-A927-01	TCGA-VQ-A927	0	no alteration
stad_tcga_pan_can_atlas_2018	TCGA-VQ-A928-01	TCGA-VQ-A928	0	no alteration
stad_tcga_pan_can_atlas_2018	TCGA-VQ-A92D-01	TCGA-VQ-A92D	0	no alteration
stad_tcga_pan_can_atlas_2018	TCGA-VQ-A94O-01	TCGA-VQ-A94O	0	no alteration
stad_tcga_pan_can_atlas_2018	TCGA-VQ-A94R-01	TCGA-VQ-A94R	0	no alteration
stad_tcga_pan_can_atlas_2018	TCGA-VQ-A94U-01	TCGA-VQ-A94U	0	no alteration
stad_tcga_pan_can_atlas_2018	TCGA-VQ-AA64-01	TCGA-VQ-AA64	0	no alteration
stad_tcga_pan_can_atlas_2018	TCGA-VQ-AA69-01	TCGA-VQ-AA69	0	no alteration
stad_tcga_pan_can_atlas_2018	TCGA-VQ-AA6A-01	TCGA-VQ-AA6A	0	no alteration
stad_tcga_pan_can_atlas_2018	TCGA-VQ-AA6B-01	TCGA-VQ-AA6B	0	no alteration
stad_tcga_pan_can_atlas_2018	TCGA-VQ-AA6D-01	TCGA-VQ-AA6D	0	no alteration
stad_tcga_pan_can_atlas_2018	TCGA-VQ-AA6F-01	TCGA-VQ-AA6F	0	no alteration
stad_tcga_pan_can_atlas_2018	TCGA-VQ-AA6G-01	TCGA-VQ-AA6G	0	no alteration
stad_tcga_pan_can_atlas_2018	TCGA-VQ-AA6I-01	TCGA-VQ-AA6I	0	no alteration
stad_tcga_pan_can_atlas_2018	TCGA-VQ-AA6J-01	TCGA-VQ-AA6J	0	no alteration
stad_tcga_pan_can_atlas_2018	TCGA-VQ-AA6K-01	TCGA-VQ-AA6K	0	no alteration
stad_tcga_pan_can_atlas_2018	TCGA-ZA-A8F6-01	TCGA-ZA-A8F6	0	no alteration
stad_tcga_pan_can_atlas_2018	TCGA-ZQ-A9CR-01	TCGA-ZQ-A9CR	0	no alteration

RIOX2: AMP	RIOX2: HOMDEL	RIOX2: EXP>=2	RIOX2: EXP<=-2	RIOX2: MUT	RIOX2: FUSION
no alteration	no alteration	HIGH	HIGH	no alteration	no alteration
no alteration	no alteration	no alteration	no alteration	I150F	no alteration
no alteration	no alteration	LOW	LOW	no alteration	no alteration
no alteration	no alteration	no alteration	no alteration	S91G	no alteration
no alteration	no alteration	HIGH	HIGH	no alteration	no alteration
no alteration	no alteration	HIGH	HIGH	no alteration	no alteration
no alteration	no alteration	HIGH	HIGH	no alteration	no alteration
no alteration	no alteration	no alteration	no alteration	C105R	no alteration
no alteration	no alteration	HIGH	HIGH	no alteration	no alteration
no alteration	no alteration	HIGH	HIGH	no alteration	no alteration
no alteration	no alteration	HIGH	HIGH	no alteration	no alteration
no alteration	no alteration	LOW	LOW	no alteration	no alteration
no alteration	no alteration	HIGH	HIGH	no alteration	no alteration
no alteration	no alteration	HIGH	HIGH	no alteration	no alteration
no alteration	no alteration	HIGH	HIGH	no alteration	no alteration
no alteration	no alteration	HIGH	HIGH	no alteration	no alteration
no alteration	no alteration	HIGH	HIGH	no alteration	no alteration
no alteration	no alteration	HIGH	HIGH	no alteration	no alteration
no alteration	no alteration	HIGH	HIGH	no alteration	no alteration
no alteration	no alteration	no alteration	no alteration	S210N	no alteration
no alteration	no alteration	LOW	LOW	no alteration	no alteration
no alteration	no alteration	LOW	LOW	no alteration	no alteration
no alteration	no alteration	LOW	LOW	no alteration	no alteration
no alteration	no alteration	HIGH	HIGH	no alteration	no alteration
no alteration	no alteration	HIGH	HIGH	no alteration	no alteration
no alteration	no alteration	HIGH	HIGH	no alteration	no alteration
no alteration	no alteration	HIGH	HIGH	no alteration	no alteration
no alteration	no alteration	HIGH	HIGH	no alteration	no alteration
no alteration	no alteration	HIGH	HIGH	no alteration	no alteration
no alteration	no alteration	LOW	LOW	no alteration	no alteration
no alteration	no alteration	no alteration	no alteration	P16Rfs*7	no alteration
no alteration	no alteration	HIGH	HIGH	no alteration	no alteration
no alteration	no alteration	HIGH	HIGH	no alteration	no alteration
no alteration	no alteration	HIGH	HIGH	no alteration	no alteration
no alteration	no alteration	HIGH	HIGH	no alteration	no alteration
no alteration	no alteration	HIGH	HIGH	no alteration	no alteration
no alteration	no alteration	HIGH	HIGH	E413K	no alteration
no alteration	no alteration	HIGH	HIGH	no alteration	no alteration
no alteration	no alteration	HIGH	HIGH	no alteration	no alteration
no alteration	no alteration	HIGH	HIGH	no alteration	no alteration
no alteration	no alteration	HIGH	HIGH	no alteration	no alteration
AMP	AMP	no alteration	no alteration	no alteration	no alteration

no alteration	no alteration	HIGH	HIGH	no alteration	no alteration
no alteration	no alteration	HIGH	HIGH	no alteration	no alteration
no alteration	no alteration	HIGH	HIGH	no alteration	no alteration
no alteration	no alteration	no alteration	no alteration	no alteration	no alteration
no alteration	no alteration	no alteration	no alteration	no alteration	no alteration
no alteration	no alteration	no alteration	no alteration	no alteration	no alteration
no alteration	no alteration	no alteration	no alteration	no alteration	no alteration
no alteration	no alteration	no alteration	no alteration	no alteration	no alteration
no alteration	no alteration	no alteration	no alteration	no alteration	no alteration
no alteration	no alteration	no alteration	no alteration	no alteration	no alteration
no alteration	no alteration	no alteration	no alteration	no alteration	no alteration
no alteration	no alteration	no alteration	no alteration	no alteration	no alteration
no alteration	no alteration	no alteration	no alteration	no alteration	no alteration
no alteration	no alteration	no alteration	no alteration	no alteration	no alteration
no alteration	no alteration	no alteration	no alteration	no alteration	no alteration
no alteration	no alteration	no alteration	no alteration	no alteration	no alteration
no alteration	no alteration	no alteration	no alteration	no alteration	no alteration
no alteration	no alteration	no alteration	no alteration	no alteration	no alteration
no alteration	no alteration	no alteration	no alteration	no alteration	no alteration
no alteration	no alteration	no alteration	no alteration	no alteration	no alteration
no alteration	no alteration	no alteration	no alteration	no alteration	no alteration
no alteration	no alteration	no alteration	no alteration	no alteration	no alteration
no alteration	no alteration	no alteration	no alteration	no alteration	no alteration
no alteration	no alteration	no alteration	no alteration	no alteration	no alteration
no alteration	no alteration	no alteration	no alteration	no alteration	no alteration
no alteration	no alteration	no alteration	no alteration	no alteration	no alteration
no alteration	no alteration	no alteration	no alteration	no alteration	no alteration
no alteration	no alteration	no alteration	no alteration	no alteration	no alteration
no alteration	no alteration	no alteration	no alteration	no alteration	no alteration
no alteration	no alteration	no alteration	no alteration	no alteration	no alteration
no alteration	no alteration	no alteration	no alteration	no alteration	no alteration
no alteration	no alteration	no alteration	no alteration	no alteration	no alteration
no alteration	no alteration	no alteration	no alteration	no alteration	no alteration
no alteration	no alteration	no alteration	no alteration	no alteration	no alteration
no alteration	no alteration	no alteration	no alteration	no alteration	no alteration
no alteration	no alteration	no alteration	no alteration	no alteration	no alteration
no alteration	no alteration	no alteration	no alteration	no alteration	no alteration
no alteration	no alteration	no alteration	no alteration	no alteration	no alteration
no alteration	no alteration	no alteration	no alteration	no alteration	no alteration
no alteration	no alteration	no alteration	no alteration	no alteration	no alteration
no alteration	no alteration	no alteration	no alteration	no alteration	no alteration
no alteration	no alteration	no alteration	no alteration	no alteration	no alteration
no alteration	no alteration	no alteration	no alteration	no alteration	no alteration

no alteration	no alteration	no alteration	no alteration	no alteration	no alteration
no alteration	no alteration	no alteration	no alteration	no alteration	no alteration
no alteration	no alteration	no alteration	no alteration	no alteration	no alteration
no alteration	no alteration	no alteration	no alteration	no alteration	no alteration
no alteration	no alteration	no alteration	no alteration	no alteration	no alteration
no alteration	no alteration	no alteration	no alteration	no alteration	no alteration
no alteration	no alteration	no alteration	no alteration	no alteration	no alteration
no alteration	no alteration	no alteration	no alteration	no alteration	no alteration
no alteration	no alteration	no alteration	no alteration	no alteration	no alteration
no alteration	no alteration	no alteration	no alteration	no alteration	no alteration
no alteration	no alteration	no alteration	no alteration	no alteration	no alteration
no alteration	no alteration	no alteration	no alteration	no alteration	no alteration
no alteration	no alteration	no alteration	no alteration	no alteration	no alteration
no alteration	no alteration	no alteration	no alteration	no alteration	no alteration
no alteration	no alteration	no alteration	no alteration	no alteration	no alteration
no alteration	no alteration	no alteration	no alteration	no alteration	no alteration
no alteration	no alteration	no alteration	no alteration	no alteration	no alteration
no alteration	no alteration	no alteration	no alteration	no alteration	no alteration
no alteration	no alteration	no alteration	no alteration	no alteration	no alteration
no alteration	no alteration	no alteration	no alteration	no alteration	no alteration
no alteration	no alteration	no alteration	no alteration	no alteration	no alteration
no alteration	no alteration	no alteration	no alteration	no alteration	no alteration
no alteration	no alteration	no alteration	no alteration	no alteration	no alteration
no alteration	no alteration	no alteration	no alteration	no alteration	no alteration
no alteration	no alteration	no alteration	no alteration	no alteration	no alteration
no alteration	no alteration	no alteration	no alteration	no alteration	no alteration
no alteration	no alteration	no alteration	no alteration	no alteration	no alteration
no alteration	no alteration	no alteration	no alteration	no alteration	no alteration
no alteration	no alteration	no alteration	no alteration	no alteration	no alteration
no alteration	no alteration	no alteration	no alteration	no alteration	no alteration
no alteration	no alteration	no alteration	no alteration	no alteration	no alteration
no alteration	no alteration	no alteration	no alteration	no alteration	no alteration
no alteration	no alteration	no alteration	no alteration	no alteration	no alteration
no alteration	no alteration	no alteration	no alteration	no alteration	no alteration
no alteration	no alteration	no alteration	no alteration	no alteration	no alteration
no alteration	no alteration	no alteration	no alteration	no alteration	no alteration
no alteration	no alteration	no alteration	no alteration	no alteration	no alteration
no alteration	no alteration	no alteration	no alteration	no alteration	no alteration
no alteration	no alteration	no alteration	no alteration	no alteration	no alteration
no alteration	no alteration	no alteration	no alteration	no alteration	no alteration
no alteration	no alteration	no alteration	no alteration	no alteration	no alteration
no alteration	no alteration	no alteration	no alteration	no alteration	no alteration
no alteration	no alteration	no alteration	no alteration	no alteration	no alteration

no alteration	no alteration	no alteration	no alteration	no alteration	no alteration
no alteration	no alteration	no alteration	no alteration	no alteration	no alteration
no alteration	no alteration	no alteration	no alteration	no alteration	no alteration
no alteration	no alteration	no alteration	no alteration	no alteration	no alteration
no alteration	no alteration	no alteration	no alteration	no alteration	no alteration
no alteration	no alteration	no alteration	no alteration	no alteration	no alteration
no alteration	no alteration	no alteration	no alteration	no alteration	no alteration
no alteration	no alteration	no alteration	no alteration	no alteration	no alteration
no alteration	no alteration	no alteration	no alteration	no alteration	no alteration
no alteration	no alteration	no alteration	no alteration	no alteration	no alteration
no alteration	no alteration	no alteration	no alteration	no alteration	no alteration
no alteration	no alteration	no alteration	no alteration	no alteration	no alteration
no alteration	no alteration	no alteration	no alteration	no alteration	no alteration
no alteration	no alteration	no alteration	no alteration	no alteration	no alteration
no alteration	no alteration	no alteration	no alteration	no alteration	no alteration
no alteration	no alteration	no alteration	no alteration	no alteration	no alteration
no alteration	no alteration	no alteration	no alteration	no alteration	no alteration
no alteration	no alteration	no alteration	no alteration	no alteration	no alteration
no alteration	no alteration	no alteration	no alteration	no alteration	no alteration
no alteration	no alteration	no alteration	no alteration	no alteration	no alteration
no alteration	no alteration	no alteration	no alteration	no alteration	no alteration
no alteration	no alteration	no alteration	no alteration	no alteration	no alteration
no alteration	no alteration	no alteration	no alteration	no alteration	no alteration
no alteration	no alteration	no alteration	no alteration	no alteration	no alteration
no alteration	no alteration	no alteration	no alteration	no alteration	no alteration
no alteration	no alteration	no alteration	no alteration	no alteration	no alteration
no alteration	no alteration	no alteration	no alteration	no alteration	no alteration
no alteration	no alteration	no alteration	no alteration	no alteration	no alteration
no alteration	no alteration	no alteration	no alteration	no alteration	no alteration
no alteration	no alteration	no alteration	no alteration	no alteration	no alteration
no alteration	no alteration	no alteration	no alteration	no alteration	no alteration
no alteration	no alteration	no alteration	no alteration	no alteration	no alteration
no alteration	no alteration	no alteration	no alteration	no alteration	no alteration
no alteration	no alteration	no alteration	no alteration	no alteration	no alteration
no alteration	no alteration	no alteration	no alteration	no alteration	no alteration
no alteration	no alteration	no alteration	no alteration	no alteration	no alteration
no alteration	no alteration	no alteration	no alteration	no alteration	no alteration
no alteration	no alteration	no alteration	no alteration	no alteration	no alteration
no alteration	no alteration	no alteration	no alteration	no alteration	no alteration
no alteration	no alteration	no alteration	no alteration	no alteration	no alteration
no alteration	no alteration	no alteration	no alteration	no alteration	no alteration
no alteration	no alteration	no alteration	no alteration	no alteration	no alteration
no alteration	no alteration	no alteration	no alteration	no alteration	no alteration

no alteration	no alteration	no alteration	no alteration	no alteration	no alteration
no alteration	no alteration	no alteration	no alteration	no alteration	no alteration
no alteration	no alteration	no alteration	no alteration	no alteration	no alteration
no alteration	no alteration	no alteration	no alteration	no alteration	no alteration
no alteration	no alteration	no alteration	no alteration	no alteration	no alteration
no alteration	no alteration	no alteration	no alteration	no alteration	no alteration
no alteration	no alteration	no alteration	no alteration	no alteration	no alteration
no alteration	no alteration	no alteration	no alteration	no alteration	no alteration
no alteration	no alteration	no alteration	no alteration	no alteration	no alteration
no alteration	no alteration	no alteration	no alteration	no alteration	no alteration
no alteration	no alteration	no alteration	no alteration	no alteration	no alteration
no alteration	no alteration	no alteration	no alteration	no alteration	no alteration
no alteration	no alteration	no alteration	no alteration	no alteration	no alteration
no alteration	no alteration	no alteration	no alteration	no alteration	no alteration
no alteration	no alteration	no alteration	no alteration	no alteration	no alteration
no alteration	no alteration	no alteration	no alteration	no alteration	no alteration
no alteration	no alteration	no alteration	no alteration	no alteration	no alteration
no alteration	no alteration	no alteration	no alteration	no alteration	no alteration
no alteration	no alteration	no alteration	no alteration	no alteration	no alteration
no alteration	no alteration	no alteration	no alteration	no alteration	no alteration
no alteration	no alteration	no alteration	no alteration	no alteration	no alteration
no alteration	no alteration	no alteration	no alteration	no alteration	no alteration
no alteration	no alteration	no alteration	no alteration	no alteration	no alteration
no alteration	no alteration	no alteration	no alteration	no alteration	no alteration
no alteration	no alteration	no alteration	no alteration	no alteration	no alteration
no alteration	no alteration	no alteration	no alteration	no alteration	no alteration
no alteration	no alteration	no alteration	no alteration	no alteration	no alteration
no alteration	no alteration	no alteration	no alteration	no alteration	no alteration
no alteration	no alteration	no alteration	no alteration	no alteration	no alteration
no alteration	no alteration	no alteration	no alteration	no alteration	no alteration
no alteration	no alteration	no alteration	no alteration	no alteration	no alteration
no alteration	no alteration	no alteration	no alteration	no alteration	no alteration
no alteration	no alteration	no alteration	no alteration	no alteration	no alteration
no alteration	no alteration	no alteration	no alteration	no alteration	no alteration
no alteration	no alteration	no alteration	no alteration	no alteration	no alteration
no alteration	no alteration	no alteration	no alteration	no alteration	no alteration
no alteration	no alteration	no alteration	no alteration	no alteration	no alteration
no alteration	no alteration	no alteration	no alteration	no alteration	no alteration
no alteration	no alteration	no alteration	no alteration	no alteration	no alteration
no alteration	no alteration	no alteration	no alteration	no alteration	no alteration
no alteration	no alteration	no alteration	no alteration	no alteration	no alteration
no alteration	no alteration	no alteration	no alteration	no alteration	no alteration
no alteration	no alteration	no alteration	no alteration	no alteration	no alteration

no alteration	no alteration	no alteration	no alteration	no alteration	no alteration
no alteration	no alteration	no alteration	no alteration	no alteration	no alteration
no alteration	no alteration	no alteration	no alteration	no alteration	no alteration
no alteration	no alteration	no alteration	no alteration	no alteration	no alteration
no alteration	no alteration	no alteration	no alteration	no alteration	no alteration
no alteration	no alteration	no alteration	no alteration	no alteration	no alteration
no alteration	no alteration	no alteration	no alteration	no alteration	no alteration
no alteration	no alteration	no alteration	no alteration	no alteration	no alteration
no alteration	no alteration	no alteration	no alteration	no alteration	no alteration
no alteration	no alteration	no alteration	no alteration	no alteration	no alteration
no alteration	no alteration	no alteration	no alteration	no alteration	no alteration
no alteration	no alteration	no alteration	no alteration	no alteration	no alteration
no alteration	no alteration	no alteration	no alteration	no alteration	no alteration
no alteration	no alteration	no alteration	no alteration	no alteration	no alteration
no alteration	no alteration	no alteration	no alteration	no alteration	no alteration
no alteration	no alteration	no alteration	no alteration	no alteration	no alteration
no alteration	no alteration	no alteration	no alteration	no alteration	no alteration
no alteration	no alteration	no alteration	no alteration	no alteration	no alteration
no alteration	no alteration	no alteration	no alteration	no alteration	no alteration
no alteration	no alteration	no alteration	no alteration	no alteration	no alteration
no alteration	no alteration	no alteration	no alteration	no alteration	no alteration
no alteration	no alteration	no alteration	no alteration	no alteration	no alteration
no alteration	no alteration	no alteration	no alteration	no alteration	no alteration
no alteration	no alteration	no alteration	no alteration	no alteration	no alteration
no alteration	no alteration	no alteration	no alteration	no alteration	no alteration
no alteration	no alteration	no alteration	no alteration	no alteration	no alteration
no alteration	no alteration	no alteration	no alteration	no alteration	no alteration
no alteration	no alteration	no alteration	no alteration	no alteration	no alteration
no alteration	no alteration	no alteration	no alteration	no alteration	no alteration
no alteration	no alteration	no alteration	no alteration	no alteration	no alteration
no alteration	no alteration	no alteration	no alteration	no alteration	no alteration
no alteration	no alteration	no alteration	no alteration	no alteration	no alteration
no alteration	no alteration	no alteration	no alteration	no alteration	no alteration
no alteration	no alteration	no alteration	no alteration	no alteration	no alteration
no alteration	no alteration	no alteration	no alteration	no alteration	no alteration
no alteration	no alteration	no alteration	no alteration	no alteration	no alteration
no alteration	no alteration	no alteration	no alteration	no alteration	no alteration
no alteration	no alteration	no alteration	no alteration	no alteration	no alteration
no alteration	no alteration	no alteration	no alteration	no alteration	no alteration
no alteration	no alteration	no alteration	no alteration	no alteration	no alteration
no alteration	no alteration	no alteration	no alteration	no alteration	no alteration
no alteration	no alteration	no alteration	no alteration	no alteration	no alteration
no alteration	no alteration	no alteration	no alteration	no alteration	no alteration

no alteration	no alteration	no alteration	no alteration	no alteration	no alteration
no alteration	no alteration	no alteration	no alteration	no alteration	no alteration
no alteration	no alteration	no alteration	no alteration	no alteration	no alteration
no alteration	no alteration	no alteration	no alteration	no alteration	no alteration
no alteration	no alteration	no alteration	no alteration	no alteration	no alteration
no alteration	no alteration	no alteration	no alteration	no alteration	no alteration
no alteration	no alteration	no alteration	no alteration	no alteration	no alteration
no alteration	no alteration	no alteration	no alteration	no alteration	no alteration
no alteration	no alteration	no alteration	no alteration	no alteration	no alteration
no alteration	no alteration	no alteration	no alteration	no alteration	no alteration
no alteration	no alteration	no alteration	no alteration	no alteration	no alteration
no alteration	no alteration	no alteration	no alteration	no alteration	no alteration
no alteration	no alteration	no alteration	no alteration	no alteration	no alteration
no alteration	no alteration	no alteration	no alteration	no alteration	no alteration
no alteration	no alteration	no alteration	no alteration	no alteration	no alteration
no alteration	no alteration	no alteration	no alteration	no alteration	no alteration
no alteration	no alteration	no alteration	no alteration	no alteration	no alteration
no alteration	no alteration	no alteration	no alteration	no alteration	no alteration
no alteration	no alteration	no alteration	no alteration	no alteration	no alteration
no alteration	no alteration	no alteration	no alteration	no alteration	no alteration
no alteration	no alteration	no alteration	no alteration	no alteration	no alteration
no alteration	no alteration	no alteration	no alteration	no alteration	no alteration
no alteration	no alteration	no alteration	no alteration	no alteration	no alteration
no alteration	no alteration	no alteration	no alteration	no alteration	no alteration
no alteration	no alteration	no alteration	no alteration	no alteration	no alteration
no alteration	no alteration	no alteration	no alteration	no alteration	no alteration
no alteration	no alteration	no alteration	no alteration	no alteration	no alteration
no alteration	no alteration	no alteration	no alteration	no alteration	no alteration
no alteration	no alteration	no alteration	no alteration	no alteration	no alteration
no alteration	no alteration	no alteration	no alteration	no alteration	no alteration
no alteration	no alteration	no alteration	no alteration	no alteration	no alteration
no alteration	no alteration	no alteration	no alteration	no alteration	no alteration
no alteration	no alteration	no alteration	no alteration	no alteration	no alteration
no alteration	no alteration	no alteration	no alteration	no alteration	no alteration
no alteration	no alteration	no alteration	no alteration	no alteration	no alteration
no alteration	no alteration	no alteration	no alteration	no alteration	no alteration
no alteration	no alteration	no alteration	no alteration	no alteration	no alteration
no alteration	no alteration	no alteration	no alteration	no alteration	no alteration
no alteration	no alteration	no alteration	no alteration	no alteration	no alteration
no alteration	no alteration	no alteration	no alteration	no alteration	no alteration
no alteration	no alteration	no alteration	no alteration	no alteration	no alteration
no alteration	no alteration	no alteration	no alteration	no alteration	no alteration
no alteration	no alteration	no alteration	no alteration	no alteration	no alteration

no alteration	no alteration	no alteration	no alteration	no alteration	no alteration
no alteration	no alteration	no alteration	no alteration	no alteration	no alteration
no alteration	no alteration	no alteration	no alteration	no alteration	no alteration
no alteration	no alteration	no alteration	no alteration	no alteration	no alteration
no alteration	no alteration	no alteration	no alteration	no alteration	no alteration
no alteration	no alteration	no alteration	no alteration	no alteration	no alteration
no alteration	no alteration	no alteration	no alteration	no alteration	no alteration
no alteration	no alteration	no alteration	no alteration	no alteration	no alteration
no alteration	no alteration	no alteration	no alteration	no alteration	no alteration
no alteration	no alteration	no alteration	no alteration	no alteration	no alteration
no alteration	no alteration	no alteration	no alteration	no alteration	no alteration
no alteration	no alteration	no alteration	no alteration	no alteration	no alteration
no alteration	no alteration	no alteration	no alteration	no alteration	no alteration
no alteration	no alteration	no alteration	no alteration	no alteration	no alteration
no alteration	no alteration	no alteration	no alteration	no alteration	no alteration
no alteration	no alteration	no alteration	no alteration	no alteration	no alteration
no alteration	no alteration	no alteration	no alteration	no alteration	no alteration
no alteration	no alteration	no alteration	no alteration	no alteration	no alteration
no alteration	no alteration	no alteration	no alteration	no alteration	no alteration
no alteration	no alteration	no alteration	no alteration	no alteration	no alteration
no alteration	no alteration	no alteration	no alteration	no alteration	no alteration
no alteration	no alteration	no alteration	no alteration	no alteration	no alteration
no alteration	no alteration	no alteration	no alteration	no alteration	no alteration
no alteration	no alteration	no alteration	no alteration	no alteration	no alteration
no alteration	no alteration	no alteration	no alteration	no alteration	no alteration
no alteration	no alteration	no alteration	no alteration	no alteration	no alteration
no alteration	no alteration	no alteration	no alteration	no alteration	no alteration
no alteration	no alteration	no alteration	no alteration	no alteration	no alteration
no alteration	no alteration	no alteration	no alteration	no alteration	no alteration
no alteration	no alteration	no alteration	no alteration	no alteration	no alteration
no alteration	no alteration	no alteration	no alteration	no alteration	no alteration
no alteration	no alteration	no alteration	no alteration	no alteration	no alteration
no alteration	no alteration	no alteration	no alteration	no alteration	no alteration
no alteration	no alteration	no alteration	no alteration	no alteration	no alteration
no alteration	no alteration	no alteration	no alteration	no alteration	no alteration
no alteration	no alteration	no alteration	no alteration	no alteration	no alteration
no alteration	no alteration	no alteration	no alteration	no alteration	no alteration
no alteration	no alteration	no alteration	no alteration	no alteration	no alteration
no alteration	no alteration	no alteration	no alteration	no alteration	no alteration
no alteration	no alteration	no alteration	no alteration	no alteration	no alteration
no alteration	no alteration	no alteration	no alteration	no alteration	no alteration
no alteration	no alteration	no alteration	no alteration	no alteration	no alteration
no alteration	no alteration	no alteration	no alteration	no alteration	no alteration

no alteration	no alteration	no alteration	no alteration	no alteration	no alteration
no alteration	no alteration	no alteration	no alteration	no alteration	no alteration
no alteration	no alteration	no alteration	no alteration	no alteration	no alteration
no alteration	no alteration	no alteration	no alteration	no alteration	no alteration
no alteration	no alteration	no alteration	no alteration	no alteration	no alteration
no alteration	no alteration	no alteration	no alteration	no alteration	no alteration
no alteration	no alteration	no alteration	no alteration	no alteration	no alteration
no alteration	no alteration	no alteration	no alteration	no alteration	no alteration
no alteration	no alteration	no alteration	no alteration	no alteration	no alteration
no alteration	no alteration	no alteration	no alteration	no alteration	no alteration
no alteration	no alteration	no alteration	no alteration	no alteration	no alteration
no alteration	no alteration	no alteration	no alteration	no alteration	no alteration
no alteration	no alteration	no alteration	no alteration	no alteration	no alteration
no alteration	no alteration	no alteration	no alteration	no alteration	no alteration
no alteration	no alteration	no alteration	no alteration	no alteration	no alteration
no alteration	no alteration	no alteration	no alteration	no alteration	no alteration
no alteration	no alteration	no alteration	no alteration	no alteration	no alteration
no alteration	no alteration	no alteration	no alteration	no alteration	no alteration
no alteration	no alteration	no alteration	no alteration	no alteration	no alteration
no alteration	no alteration	no alteration	no alteration	no alteration	no alteration
no alteration	no alteration	no alteration	no alteration	no alteration	no alteration
no alteration	no alteration	no alteration	no alteration	no alteration	no alteration
no alteration	no alteration	no alteration	no alteration	no alteration	no alteration
no alteration	no alteration	no alteration	no alteration	no alteration	no alteration
no alteration	no alteration	no alteration	no alteration	no alteration	no alteration
no alteration	no alteration	no alteration	no alteration	no alteration	no alteration
no alteration	no alteration	no alteration	no alteration	no alteration	no alteration
no alteration	no alteration	no alteration	no alteration	no alteration	no alteration
no alteration	no alteration	no alteration	no alteration	no alteration	no alteration
no alteration	no alteration	no alteration	no alteration	no alteration	no alteration
no alteration	no alteration	no alteration	no alteration	no alteration	no alteration
no alteration	no alteration	no alteration	no alteration	no alteration	no alteration
no alteration	no alteration	no alteration	no alteration	no alteration	no alteration
no alteration	no alteration	no alteration	no alteration	no alteration	no alteration
no alteration	no alteration	no alteration	no alteration	no alteration	no alteration
no alteration	no alteration	no alteration	no alteration	no alteration	no alteration
no alteration	no alteration	no alteration	no alteration	no alteration	no alteration
no alteration	no alteration	no alteration	no alteration	no alteration	no alteration
no alteration	no alteration	no alteration	no alteration	no alteration	no alteration
no alteration	no alteration	no alteration	no alteration	no alteration	no alteration
no alteration	no alteration	no alteration	no alteration	no alteration	no alteration
no alteration	no alteration	no alteration	no alteration	no alteration	no alteration
no alteration	no alteration	no alteration	no alteration	no alteration	no alteration

no alteration	no alteration	no alteration	no alteration	no alteration	no alteration
no alteration	no alteration	no alteration	no alteration	no alteration	no alteration
no alteration	no alteration	no alteration	no alteration	no alteration	no alteration
no alteration	no alteration	no alteration	no alteration	no alteration	no alteration
no alteration	no alteration	no alteration	no alteration	no alteration	no alteration
no alteration	no alteration	no alteration	no alteration	no alteration	no alteration
no alteration	no alteration	no alteration	no alteration	no alteration	no alteration
no alteration	no alteration	no alteration	no alteration	no alteration	no alteration
no alteration	no alteration	no alteration	no alteration	no alteration	no alteration
no alteration	no alteration	no alteration	no alteration	no alteration	no alteration
no alteration	no alteration	no alteration	no alteration	no alteration	no alteration
no alteration	no alteration	no alteration	no alteration	no alteration	no alteration
no alteration	no alteration	no alteration	no alteration	no alteration	no alteration
no alteration	no alteration	no alteration	no alteration	no alteration	no alteration
no alteration	no alteration	no alteration	no alteration	no alteration	no alteration
no alteration	no alteration	no alteration	no alteration	no alteration	no alteration
no alteration	no alteration	no alteration	no alteration	no alteration	no alteration
no alteration	no alteration	no alteration	no alteration	no alteration	no alteration
no alteration	no alteration	no alteration	no alteration	no alteration	no alteration
no alteration	no alteration	no alteration	no alteration	no alteration	no alteration
no alteration	no alteration	no alteration	no alteration	no alteration	no alteration
